# Hypoblast from human pluripotent stem cells regulates epiblast development

**DOI:** 10.1038/s41586-023-06871-2

**Published:** 2023-12-05

**Authors:** Takumi Okubo, Nicolas Rivron, Mio Kabata, Hideki Masaki, Keiko Kishimoto, Katsunori Semi, May Nakajima-Koyama, Haruko Kunitomi, Belinda Kaswandy, Hideyuki Sato, Hiromitsu Nakauchi, Knut Woltjen, Mitinori Saitou, Erika Sasaki, Takuya Yamamoto, Yasuhiro Takashima

**Affiliations:** 1 Center for iPS Cell Research and Application, Kyoto University, Kyoto, Japan; 2 Institute of Molecular Biotechnology of the Austrian Academy of Sciences (IMBA), Vienna BioCenter (VBC), Vienna, Austria; 3 Institute of Medical Science, University of Tokyo, Tokyo, Japan; 4 Advanced Research Institute, Tokyo Medical and Dental University, Tokyo, Japan; 5 Central Institute for Experimental Animals, Kawasaki, Japan; 6 Institute for Stem Cell Biology and Regenerative Medicine, Stanford University School of Medicine, Stanford, CA USA; 7 Institute for the Advanced Study of Human Biology (WPI-ASHBi), Kyoto University, Kyoto, Japan; 8 Department of Anatomy and Cell Biology, Graduate School of Medicine, Kyoto University, Kyoto, Japan; 9 Medical-risk Avoidance Based on iPS Cells Team, RIKEN Center for Advanced Intelligence Project (AIP), Kyoto, Japan

**Keywords:** Embryonic germ cells, Embryonic induction

## Abstract

Recently, several studies using cultures of human embryos together with single-cell RNA-seq analyses have revealed differences between humans and mice, necessitating the study of human embryos^1–8^. Despite the importance of human embryology, ethical and legal restrictions have limited post-implantation-stage studies. Thus, recent efforts have focused on developing in vitro self-organizing models using human stem cells^9–17^. Here, we report genetic and non-genetic approaches to generate authentic hypoblast cells (naive hPSC-derived hypoblast-like cells (nHyCs))—known to give rise to one of the two extraembryonic tissues essential for embryonic development—from naive human pluripotent stem cells (hPSCs). Our nHyCs spontaneously assemble with naive hPSCs to form a three-dimensional bilaminar structure (bilaminoids) with a pro-amniotic-like cavity. In the presence of additional naive hPSC-derived analogues of the second extraembryonic tissue, the trophectoderm, the efficiency of bilaminoid formation increases from 20% to 40%, and the epiblast within the bilaminoids continues to develop in response to trophectoderm-secreted IL-6. Furthermore, we show that bilaminoids robustly recapitulate the patterning of the anterior–posterior axis and the formation of cells reflecting the pregastrula stage, the emergence of which can be shaped by genetically manipulating the DKK1/OTX2 hypoblast-like domain. We have therefore successfully modelled and identified the mechanisms by which the two extraembryonic tissues efficiently guide the stage-specific growth and progression of the epiblast as it establishes the post-implantation landmarks of human embryogenesis.

## Abstract (web summary)

Authentic hypoblast cells created from naive human pluripotent stem cells (hPSCs) spontaneously assemble with naive hPSCs to form a three-dimensional bilaminar structure (bilaminoids) with a pro-amniotic-like cavity.

## Main

Early blastocysts of the pre-implantation human embryos are composed of trophectoderm and inner cell mass (ICM). The ICM generates the epiblast (that is, future fetus) and hypoblast (that is, primitive endoderm, future yolk sac), a process completed in the late blastocyst stage. During implantation, these two tissues form a bilaminar disc that functions as a developmental template for the embryo. Despite the importance of early human development, our knowledge of human peri-implantation development is limited owing to ethical and legal restrictions. Thus, alternative approaches for analysing this developmentally critical period are necessary.

To model human pre-implantation development, it is important to establish cells that correspond to pre-implantation embryos in vitro. In contrast to their mouse counterpart, naive human pluripotent stem cells (hPSCs), corresponding to the pre-implantation epiblast^18–20^, can generate blastocyst-like structures (blastoids)^16,17^ and differentiate into the trophectoderm of blastocysts^21,22^. Although hypoblast differentiation from naive hPSCs has been reported^23^, the molecular details remain unclear, and the capture of in vitro pre-implantation hypoblast has not been achieved. Thus, it remains unclear whether extraembryonic tissues support the development of pre-implantation epiblast.

Here we induced human pre-implantation hypoblast from naive hPSCs by either transgene overexpression or chemical induction, which guides the epiblast to form the first embryonic cavity, establishes the anterior–posterior axis and, together with the second extraembryonic tissue, the trophectoderm/trophoblast (TB), supports the establishment of the post-implantation embryonic state.

## Naive hPSC-induced hypoblast by *GATA6*

**Fig. 1:**
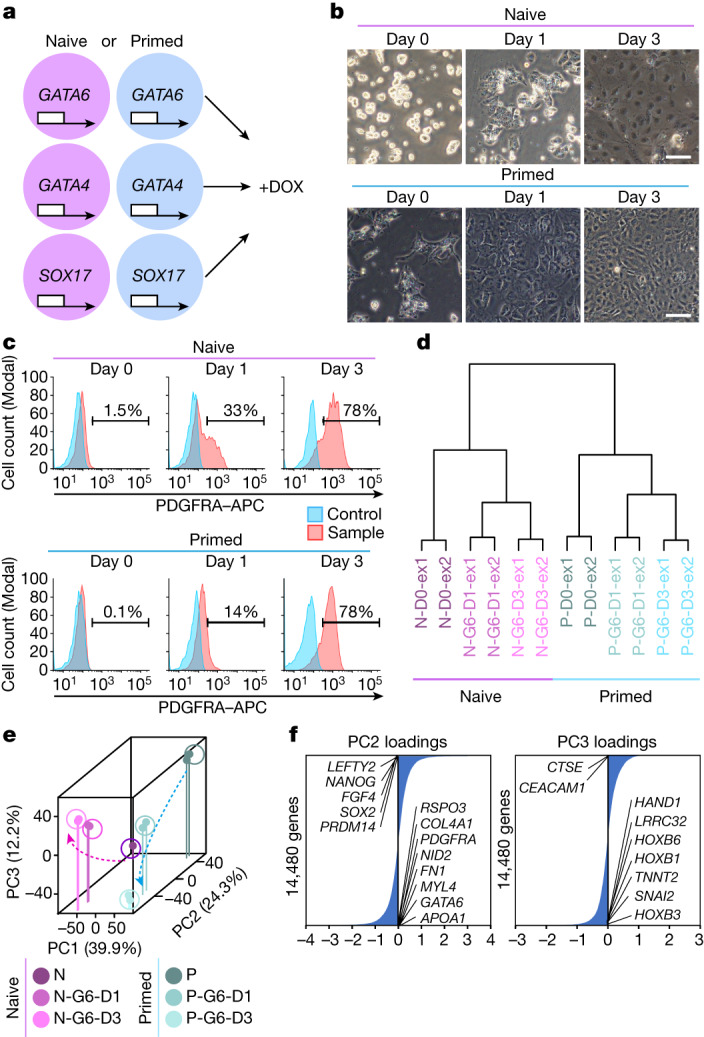
Naive hPSC differentiation into the PDGFRA^+^ hypoblast by *GATA6* overexpression. **a**, Schematic of the DOX-dependent induction of the *GATA6*, *GATA4* or *SOX17* transgene in hPSCs. **b**, Bright-field images of naive and primed H9 hPSCs (day 0 (D0)) and hPSC-derived cells with *GATA6* overexpression at D1 and D3 under serum-containing conditions (Extended Data Fig. 1o). *n* = 10. **c**, Flow cytometry analysis of PDGFRA expression in naive and primed hPSCs after *GATA6* induction under serum-free conditions (Extended Data Fig. 1o). *n* = 3. **d**, UHC analysis of the transcriptomes of naive hPSCs (N-D0), naive hPSC-derived GATA6-PDGFRA^+^ cells (N-G6-D1 and N-G6-D3), primed hPSCs (P-D0) and primed hPSC-derived G6-PDGFRA^+^ cells (P-G6-D1 and P-G6-D3) from two independent experiments (ex1 and ex2). PDGFRA^+^ cells were sorted on D1 and D3. **e**, PCA of naive and primed cells. **f**, PC2 and PC3 loadings of **e**. In total, 14,481 genes were ordered by their PC2 or PC3 loading scores (Supplementary Table 2). Representative genes among the top 50 are shown. *n* values show biologically independent experiments. Scale bars, 100 µm (**b**). Reproducibility is shown in the Methods.

**Extended Data Fig. 1:**
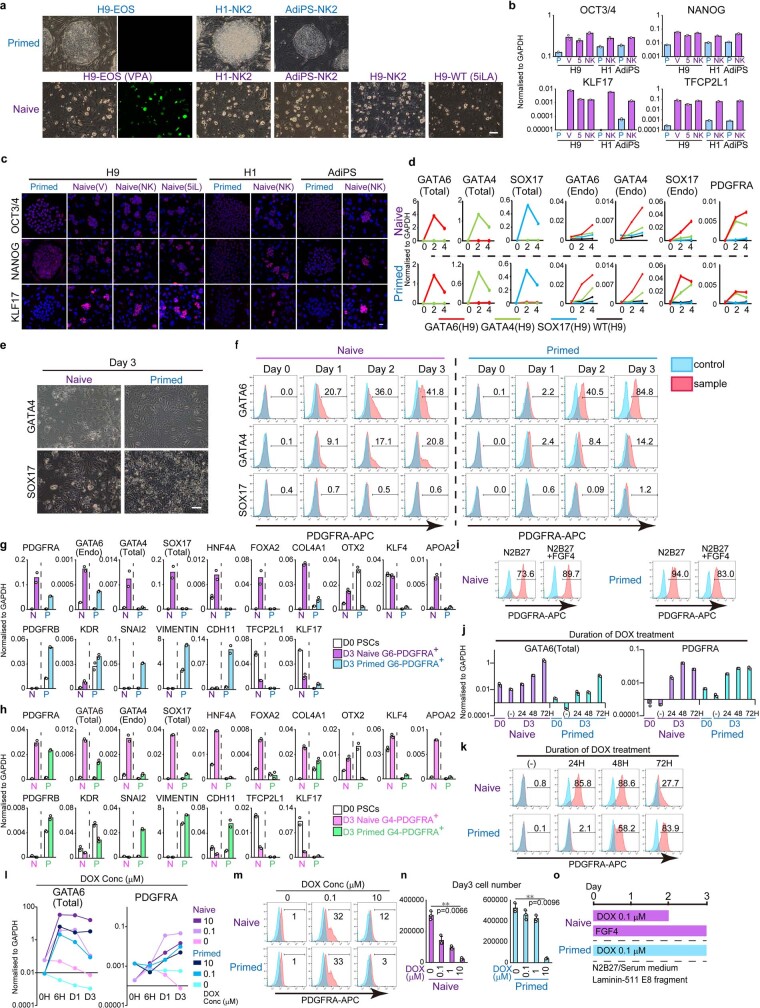
Naïve and Primed hPSCs and *GATA6* overexpression. a. Morphology of naïve and primed hPSCs. Upper panels show primed H9 ESCs, H1 ESCs, and AdiPSCs. Lower panels show naïve H9 ESCs, H1 ESCs, and AdiPSCs. EOS-GFP was expressed in naïve but not primed H9-EOS hPSCs. Primed hPSCs were cultured in KSR/FGF2. Naïve (VPA) are hPSCs reset by the method reported by Guo et al. (2017)^20^. Naïve (NK2) are hPSCs reset by the method reported by Takashima et al. (2014)^18^. Naïve (5iLA) are hPSCs reset by the method reported by Theunissen et al. (2014)^19^. After resetting, naïve hPSCs were cultured in t2iLGo. N = 2. b. Gene expression patterns of naïve and primed hPSCs. Pluripotent genes were upregulated in naïve (purple) compared with primed hPSCs (blue). *KLF17* and *TFCP2L1* were strongly upregulated in naïve hPSCs. P, primed hPSCs; V, naïve hPSCs (VPA); 5, naïve hPSCs (5iLA); NK, naïve hPSCs (NK2). N = 2. *Y*-axis shows log scale. c. Immunofluorescence of OCT3/4, NANOG, and KLF17 in naïve and primed hPSCs. Magenta, indicated proteins; Blue, DAPI. N = 2. d. Gene expression patterns of *GATA6*, *GATA4*, *SOX17*, and *PDGFRA* in H9 naïve and primed hPSCs on days (D) 0, 2, and 4 after transgene overexpression. Naïve and primed hPSCs were seeded on MEF under serum medium. DOX (0.1 μM) was added from day 0 to 4. Expression levels were normalized to *GAPDH*. GATA6(H9), hPSCs carrying a doxycycline (DOX)-inducible *GATA6* transgene, GATA4(H9), hPSCs carrying a doxycycline (DOX)-inducible *GATA4* transgene, SOX17(H9), hPSCs carrying a doxycycline (DOX)-inducible *SOX17* transgene, WT, wild type. N = 2. e. Bright-field images of naïve and primed H9 hPSC-derived cells induced with *GATA4* or *SOX17* overexpression under serum-containing conditions as depicted in (o) on D3. N = 3. f. Kinetics of PDGFRA changes after the overexpression of *GATA6*, *GATA4*, or *SOX17* transgene in naïve and primed H9 hPSCs by DOX under serum-containing conditions as in (o). PDGFRA expression was measured by flow cytometry. Blue, unstained control; Red, samples. N = 2. g. Expression levels of hypoblast and mesoderm genes in H9 naïve and primed hPSCs on D0 and corresponding PDGFRA^+^ cells after *GATA6* overexpression (G6-PDGFRA^+^) under serum-containing conditions as in (o) on D3 (see f). N, naïve; P, primed; White, D0 control; Purple, naïve G6-PDGFRA^+^ cells; Blue, primed G6-PDGFRA^+^ cells. Expression levels were normalized to *GAPDH*. N = 2. h. Expression levels of hypoblast and mesoderm genes in H9 naïve and primed hPSCs on D0 and corresponding PDGFRA^+^ cells after *GATA4* overexpression (G4-PDGFRA^+^) under serum-containing conditions as in (o) on D3. N, naïve; P, primed; White, D0 control; Purple, naïve G4-PDGFRA^+^ cells; Green, primed G4-PDGFRA^+^ cells. Expression levels were normalized to *GAPDH*. N = 2. i. PDGFRA expression of naïve and primed hPSC-derived cells with *GATA6* overexpression under N2B27 plus FGF4 on D3. Flow cytometry was performed on D3. N = 2. j. *GATA6* (Total) and *PDGFRA* expression and DOX treatment period. Naïve and primed hPSCs were cultured under N2B27 medium as in (o), except for DOX. DOX was added from D0 to D1 (24 h), D2 (48 h), or D3 (72 h). qPCR was performed on D3. In naïve hPSCs, DOX treatment for 72 h induced more *GATA6* (Total) but less *PDGFRA* expression than for 48 h. In primed hPSCs, DOX treatment for 72 h induced the highest *GATA6* (Total) and *PDGFRA* expression. N = 2. *Y*-axis shows log scale. N = 2. k. PDGFRA expression and DOX treatment period. PDGFRA expression was measured by flow cytometry in naïve and primed hPSC-derived cells with *GATA6* overexpression on D3. The culture condition was the same as in (j). In naïve hPSCs, DOX treatment for 48 h induced PDGFRA expression most efficiently. In primed hPSCs, DOX treatment for 72 h induced PDGFRA expression most efficiently. N = 3. l. *GATA6* (Total) and *PDGFRA* expression, as measured by qPCR, in naïve and primed hPSC-derived cells at 6, 24, and 72 h after DOX induction. Naïve and primed hPSCs were cultured under N2B27 as in (o) below, except for DOX. DOX (0, 0.1, or 10 μM) was added to the N2B27 medium. Ten micromolar DOX induced more *GATA6* but less *PDGFRA* expression. N = 2. *Y*-axis shows log scale. m. PDGFRA expression after DOX induction. PDGFRA expression was measured by flow cytometry in naïve and primed hPSC-derived cells with *GATA6* overexpression on D3. The culture condition was the same as in (l). One hundred nanomolar DOX induced PDGFRA most efficiently in naïve and primed hPSCs. N = 3. n. Cell number on D3 after DOX induction. The culture condition was the same as in (l). 4 x 10^5^ naïve hPSCs and 3.5 x 10^5^ primed hPSCs were seeded and counted on D3. Ten micromolar DOX treatment resulted in the lowest number of cells. N = 3. Kruskal-Wallis and Dunn’s multiple comparisons test. N = 3. o. Scheme for the PDGFRA induction by *GATA6* overexpression. Naïve hPSCs were seeded on Laminin511-E8 under N2B27 medium plus FGF4 or serum-containing medium (see Fig. 1b, Extended Data Fig. 1d–h, 2a). DOX (0.1 μM) was added from D0 to D2. Primed hPSCs were seeded on Laminin511-E8 under N2B27 or serum-containing medium. DOX (0.1 μM) was added from D0 to D3. (N=) shows biologically independent experiments. Bar charts: (n) mean ± SEM, (b), (g), (h), (j) mean. Line charts: (d), (l) mean. Scale bars: (a), 100 µm; (c), 25 µm; (e), 50 µm. Source Data

To induce the pre-implantation hypoblast, we compared the potential of naive and primed hPSCs^18–20^ to differentiate into this tissue (Extended Data Fig. 1a–c). *Gata6*, *Gata4* and *Sox17* are expressed in the mouse hypoblast^24^, and their overexpression was shown to induce embryonic stem (ES) cells to hypoblasts^25,26^. As the human hypoblast also expresses *GATA6*, *GATA4* and *SOX17*^2,3^, we introduced doxycycline (DOX)-inducible *GATA6*, *GATA4* or *SOX17* transgenes into both naive and primed H9 ES cells by piggyBac (PB) (Fig. 1a). *GATA6* overexpression induced the endogenous hypoblast genes *GATA6*, *GATA4*, *SOX17* and *PDGFRA*. *GATA4* overexpression induced these genes only moderately, but *SOX17* overexpression failed (Extended Data Fig. 1d). This suggests a hierarchy in propagating the human hypoblast program, like in mice. After 3 days of overexpression, characteristic naive hPSC morphologies disappeared (Fig. 1b and Extended Data Fig. 1e). Flow cytometry analysis confirmed that PDGFRA was expressed after *GATA6* overexpression in naive and primed hPSC-derived cells (Extended Data Fig. 1f). PDGFRA^+^ cells from naive *GATA6*-induced hPSCs (naive G6-PDGFRA^+^) expressed hypoblast marker genes, whereas primed G6-PDGFRA^+^ cells expressed mesoderm marker genes (Extended Data Fig. 1g). *GATA4* overexpression also induced PDGFRA^+^ cells, but SOX17 did not (Extended Data Fig. 1f). Naive and primed G4-PDGFRA^+^ cells expressed hypoblast and mesoderm genes, respectively (Extended Data Fig. 1h). To characterize hypoblast specification from naive hPSCs further, we developed and optimized a serum-free induction system using N2B27 chemically defined medium (NDiff 227) as a basal medium. First, we observed that *GATA6* overexpression in naive hPSCs induces PDGFRA^+^ cells under N2B27, and FGF4 addition further enhanced this induction (Extended Data Fig. 1i). *GATA6* overexpression most efficiently induced *PDGFRA* expression and PDGFRA^+^ cells in naive and primed hPSCs after 48 and 72 h, respectively (Extended Data Fig. 1j,k). We observed that 0.1 μM DOX induced *PDGFRA* expression and PDGFRA^+^ cells more effectively than 10 μM DOX (Extended Data Fig. 1l–n). On the basis of these data, we defined a hypoblast induction protocol based on *GATA6* overexpression (Extended Data Fig. 1o).

**Extended Data Fig. 2:**
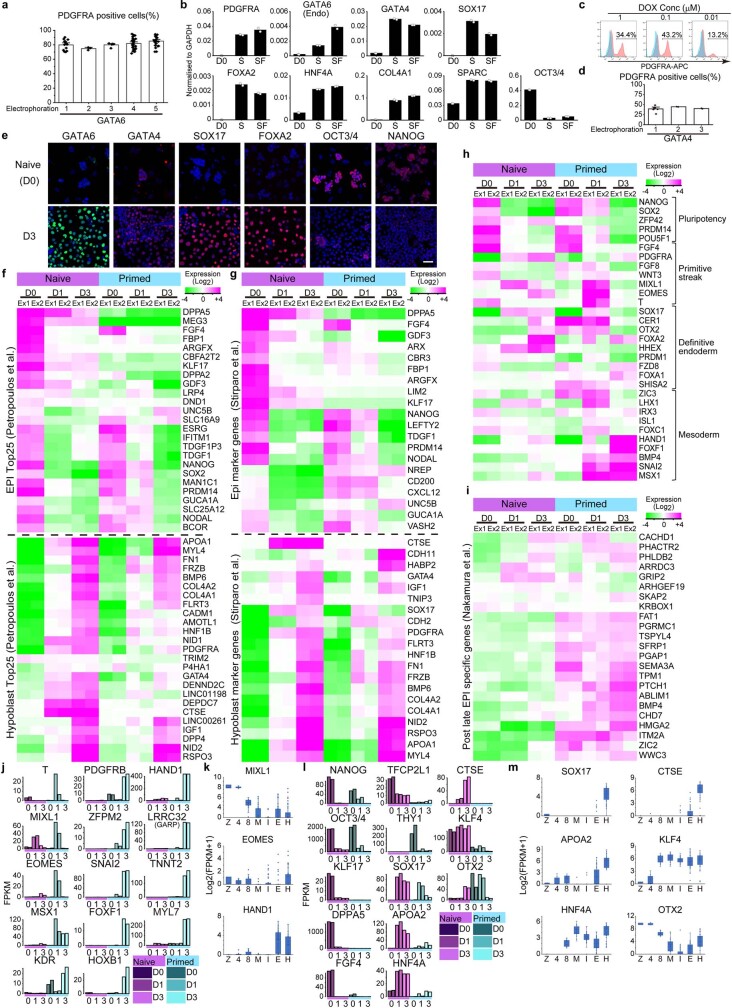
Transcriptome analysis after *GATA6* overexpression in naïve and primed G6-PDGFRA^+^ cells. a. PDGFRA expression measured by flow cytometry 3 days after *GATA6* induction in naïve H9 hPSCs. Five DOX-inducible hPSC lines were independently established. Line1 (N = 13), Line2 (N = 3), Line3 (N = 5), Line4 (N = 28), Line5 (N = 22). b. Gene expression patterns in naïve hPSCs on D0 and PGFRA^+^ cells on D3 in serum (S) or serum-free (SF) medium conditions. Expression levels were normalized to *GAPDH*. N = 2. c. PDGFRA expression after DOX induction. PDGFRA expression was measured by flow cytometry in naïve hPSC-derived cells with *GATA4* overexpression on D3. The culture condition was the same as in Extended Data Fig. 1o. One hundred nanomolar DOX induced PDGFRA expression most efficiently in naïve hPSCs. N = 2. d. PDGFRA expression measured by flow cytometry 3 days after *GATA4* induction in naïve H9 hPSCs. Three DOX-inducible hPSC lines were independently established. Line1 (N = 6), Line2 (N = 2), Line3 (N = 2). e. Immunofluorescence for hypoblast and pluripotency markers in naïve hPSCs on D0 and D3. GATA6, GATA4, SOX17, and FOXA2 were upregulated, but OCT3/4 and NANOG were downregulated in D3 naïve-derived cells after the induction of *GATA6* overexpression in SF. Red/Green, indicated proteins; Blue, DAPI. Scale bar, 50 µm. N = 2. f. Relative gene expression dynamics after *GATA6* overexpression. The top 25 Epi and hypoblast marker genes were measured by RNA-seq. Epi and hypoblast marker genes were identified by Petropoulos et al. (2016)^2^ in human embryos. All Epi marker genes were expressed in naïve hPSCs. After *GATA6* induction, most Epi genes were downregulated in D1 and D3 PDGFRA^+^ cells. However, the top 25 hypoblast marker genes were upregulated in naïve D3 PDGFRA^+^ cells. Half of the hypoblast genes were also upregulated in primed D3 PDGFRA^+^ cells. g. Relative gene expression dynamics after *GATA6* overexpression. Epi and hypoblast marker genes were identified by Stirparo et al. (2018)^7^ in human embryos. The top 20 Epi and hypoblast marker genes were measured by RNA-seq. h. Relative expression dynamics of pluripotency, primitive streak, definitive endoderm, and mesoderm genes after DOX-induced *GATA6* overexpression. The gene sets were defined by Loh et al. (2014)^71^. Primitive streak, definitive endoderm, and mesoderm genes were upregulated in primed G6-PDGFRA^+^ cells. i. Relative gene expression profiles of post-implantation Epi (Post late Epi) marker genes after DOX-induced *GATA6* overexpression. The post-implantation Epi gene set was identified by Nakamura et al. (2016)^29^ using cynomolgus pre- and post-implantation embryos. These genes were upregulated in primed hPSCs and primed hPSC-derived PDGFRA^+^ cells. j. Expression dynamics calculated by the RNA-seq analysis of primitive streak and mesoderm genes. Primed G6-PDGFRA^+^ cells displayed higher levels of primitive streak and mesoderm markers than naïve G6-PDGFRA^+^ cells. k. Gene expression profiles from the scRNA-seq analysis of human embryos reported by Stirparo et al. (2018)^7^. *MIXL1*, *EOMES*, and *HAND1* were expressed in a fraction of hypoblast cells in embryos. Expression patterns are represented as box-and-whisker plots. *Y*-axis shows Log_2_(FPKM + 1). Z, zygote (3 cells); 4, four-cell stage (10 cells); 8, eight-cell stage (16 cells); M, morula (28 cells); I, ICM (43 cells); E, Epi (54 cells); H, hypoblast (38 cells). l. Expression dynamics calculated by the RNA-seq analysis of Epi and hypoblast genes. Naïve G6-PDGFRA^+^ cells displayed an upregulation of hypoblast markers and a downregulation of pluripotency markers. m. Gene expression profiles extracted from the single-cell RNA-seq analysis of human embryos reported by Stirparo et al.^7^ confirmed the hypoblast gene expression profiles in (e). Expression patterns are shown as box-and-whisker plots. *Y*-axis shows Log_2_(FPKM + 1). Z, zygote (3 cells); 4, four-cell stage (10 cells); 8, eight-cell stage (16 cells); M, morula (28 cells); I, ICM (43 cells); E, Epi (54 cells); H, hypoblast (38 cells). (N=) shows biologically independent experiments. Box plots: (k), (m) centre line, median; box, the 25th and 75th percentiles range; whiskers, 1.5 × IQR. Bar charts: (a), (d) mean ± SEM, (b) mean. Scale bars: (e) 50 µm. Source Data

With optimized induction, *GATA6* overexpression reproducibly converted around 80% of naive hPSCs into PDGFRA^+^ cells on day 3 expressing hypoblast genes (five lines, *n* = 71; Fig. 1c, Extended Data Fig. 2a,b and Supplementary Fig. 1). *GATA4* overexpression under the same induction protocol also induced PDGFRA^+^ cells, but less efficiently than *GATA6* (Extended Data Fig. 2c,d and Supplementary Fig. 1). Hypoblast protein markers were observed after *GATA6* overexpression, whereas pluripotency markers were downregulated (Extended Data Fig. 2e).

We performed RNA-sequencing (RNA-seq) analysis during differentiation (Supplementary Table 1). Unsupervised hierarchical clustering (UHC) classified the samples on the basis of their origin (Fig. 1d). Principal component analysis (PCA) revealed that PC1 separated naive hPSCs and primed hPSCs even after differentiation (Fig. 1e). However, the similar directional transition along PC2 suggested that a common subset of genes was similarly up- or downregulated in both naive and primed G6-PDGFRA^+^ cells. During differentiation, naive hPSCs lost the expression of pre-implantation epiblast marker genes^2,7^ but upregulated hypoblast marker genes (Extended Data Fig. 2f,g). A subset of epiblast and hypoblast marker genes in primed cells also showed a similar expression pattern and strongly affected PC2 (Fig. 1f and Supplementary Table 2).

Finally, PC3 revealed a directional, progressive, but opposite transition of cellular properties in naive and primed G6-PDGFRA^+^ cells. Specifically, mesoderm and body plan genes were enriched for negative PC3 loading values (primed G6-PDGFRA^+^) (Fig. 1f and Supplementary Table 2). Previous studies reported that *PDGFRA* is expressed in mesoderm progenitors^27,28^, and *GATA6* is expressed in primitive streak/gastrulating cells and the mesoderm^8,29^. Indeed, primed G6-PDGFRA^+^ cells expressed primitive streak, definitive endoderm and mesoderm genes (Extended Data Fig. 2h) and post-implantation late epiblast marker genes in cynomolgus monkey embryos^29^ (Extended Data Fig. 2i). Moreover, primed G6-PDGFRA^+^ cells expressed early primitive streak genes on day 1 and several gastrulation- and mesoderm-related genes on day 3 (Extended Data Fig. 2j). By contrast, naive G6-PDGFRA^+^ cells did not express these mesoderm genes aside from *MIXL1*, *EOMES* and *HAND1* (Extended Data Fig. 2j), which were also detected in embryonic hypoblast cells (Extended Data Fig. 2k). Similarly, the hypoblast genes *SOX17*, *APOA2*, *HNF4A* and *CTSE* were strongly expressed only in naive G6-PDGFRA^+^ cells along with *KLF4* and *OTX2* (Extended Data Fig. 2l), which are also expressed in the hypoblast of human blastocysts (Extended Data Fig. 2m). Together, we concluded that GATA6 promotes naive hPSC differentiation into the hypoblast lineage, while primed hPSCs adopt a post-implantation embryonic fate.

## Hypoblast induced by signalling molecules

**Fig. 2:**
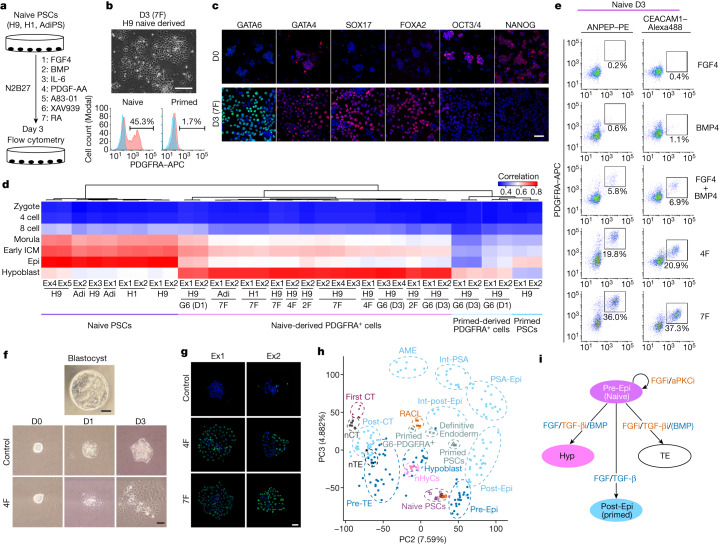
Essential signalling for human hypoblast specification. **a**, Schematic of the 7F induction of PDGFRA^+^ cells. **b**, Bright-field image and flow cytometry 3 days after 7F induction. *n* = 44. **c**, Immunofluorescence analysis of naive hPSCs at day 0 and day 3 in 7F medium. The indicated proteins are shown in red and green. Blue, DAPI. *n* = 2. **d**, Correlation coefficients of human pre-implantation embryos and naive hPSCs, primed hPSCs and PDGFRA^+^ cells in 7F, 4F or 2F, or with *GATA6* overexpression. Adi, AdiPS cells; 4F, FGF4 and BMP4 with A83-01 and XAV939; 2F, FGF4 and BMP4. **e**, Minimum essential factors for hypoblast specification. *n* = 3. **f**, Bright-field images of marmoset ICM-derived cells. ICM cells were cultured in 4F or with MEK and BMP pathway inhibitors (PD0325901 and LDN-193189) and A83 + XAV (control). *n* = 2. **g**, Immunofluorescence images of the marmoset ICM at day 3. Green, SOX17; blue, DAPI. *n* = 2. **h**, PCA of bulk RNA-seq data from this study and published reports, and of scRNA-seq data from human embryos. The circles indicate cell types^5^: blue, pre-implantation; light blue, post-implantation; pre-Epi, pre-implantation epiblast; post-Epi, post-implantation epiblast; PSA-Epi, primitive-streak anlage epiblast; int-PSA and int-post-Epi, intermediate state cells of primitive-streak anlage epiblast and post-implantation epiblast; AME, amnion; pre-TE, pre-implantation trophectoderm; post-CT, post-implantation cytotrophoblast. Bulk RNA-seq data: purple squares, naive hPSCs and nHyCs from this study; black squares, naive hPSC-derived trophectoderm (nTE) and CT (nCT)^21^; vermillion squares, naive hPSCs and RACL cells^23^; triangles, primed hPSCs and primed hPSC-derived G6 PDGFRA^+^ cells; crosses, primed hPSCs and definitive endoderm^35^; and diamonds, first-trimester primary CT^21^. **i**, Signalling pathways to specify the three cell types of blastocyst. Hyp, hypoblast; aPKCi, aPKC inhibitor; FGFi, FGF inhibitor; TGF-βi, TGFβ inhibitor. *n* values show biologically independent experiments. Scale bars, 100 µm (**b**) and 50 µm (**c**, **f** and **g**). Source Data

**Extended Data Fig. 3:**
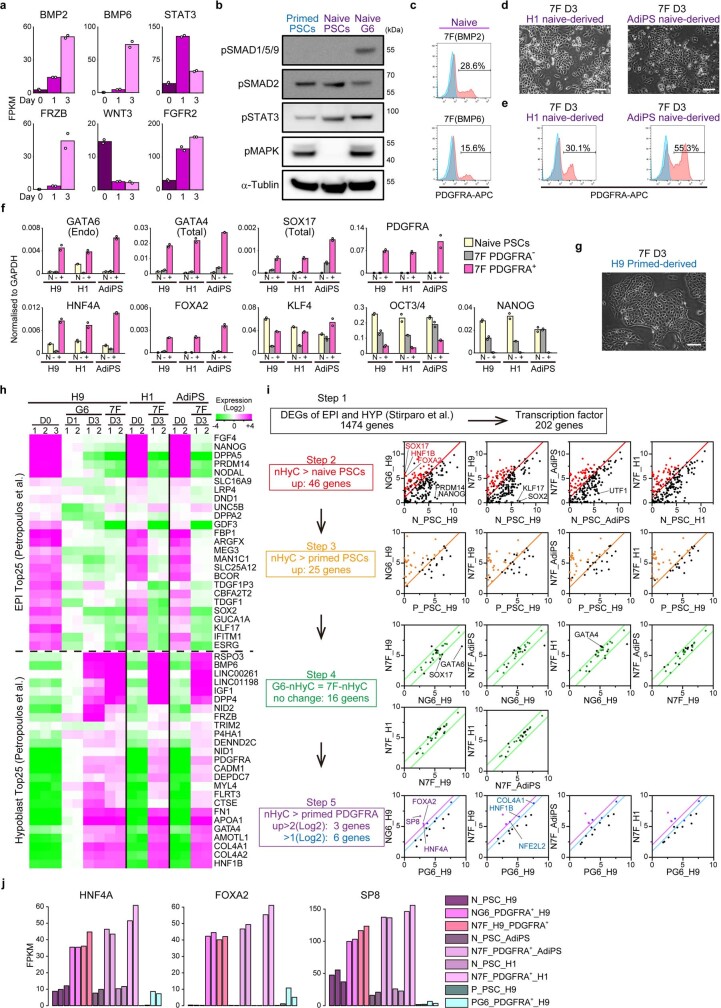
Naïve hPSCs differentiate into hypoblast lineage with 7F without *GATA6* overexpression. a. RNA expression of secreted factors and receptors after *GATA6* overexpression in naïve hPSCs on D0, D1, and D3. N = 2. b. Western blot showing SMAD1/5/9, STAT3, and MAPK phosphorylation and pSMAD2 repression after *GATA6* induction on D3. Naïve G6, naïve hPSCs after *GATA6* overexpression. N = 2. c. PDGFRA expression measured by flow cytometry 3 days after 7F induction (BMP2 or BMP6) in naïve H9 hPSCs. Blue indicates the unstained control. N = 2. d. Bright-field images of naïve H1 ESCs and naïve AdiPSCs 3 days after 7F induction. N = 2. e. PDGFRA expression was measured by flow cytometry in naïve H1 ESCs and AdiPSCs 3 days after 7F treatment. Blue indicates negative control. N = 2. f. Gene expression in naïve 7F-PDGFRA^+^ cells on D3. Naïve H9 ESCs (H9), H1 ESCs (H1), and AdiPSCs (AdiPS) were cultured in 7F medium for 3 days, with PDGFRA^+^ (+) and PDGFRA^-^ (−) cells sorted and analysed by qPCR. N = 2. g. Bright-field image of primed hPSCs 3 days after 7F induction. N = 3. h. Relative gene expression dynamics after 7F induction. The gene sets are the same as those in Extended Data Fig. 2f. H9 ESCs, H1 ESCs, and AdiPSCs were cultured in 7F or *GATA6*-overexpression conditions. PDGFRA^+^ cells were sorted on D1 (*GATA6* overexpression) or D3 (7F or *GATA6* overexpression) and analysed by RNA-seq. i. Common transcriptional factors of 7F- and G6-nHyC. (Step 1) To identify the transcriptional factors of nHyC, 202 transcriptional factors were extracted (Supplementary Table 3) from the previously reported 1474 DEGs of Epi and hypoblasts (Stirparo et al. 2018)^7^ using the Riken transcriptional database (http://genome.gsc.riken.jp/TFdb/htdocs/) and the BaseSpace correlation engine (Illumina) (Terms: Transcription, DNA-dependent). (Steps 2 and 3) Twenty-five transcriptional factors were identified as upregulated genes in nHyC (H9, H1, AdiPS) compared with naïve hPSCs and primed hPSCs (Log_2_Fold change > 2). (Step 4) To identify stably expressed transcriptional factors, 7F-nHyC and G6-nHyC were compared, and 16 genes were extracted (-2<Log_2_Fold change < 2). (Step 5) nHyC-marker transcription factors were identified by comparing nHyC and primed PDGFRA^+^ cells. According to the plots, three genes, *HNF4A, FOXA2*, and *SP8*, were expressed more than 4-fold higher in nHyC; *HNF1B*, *NFE2L2*, and *COL4A1* were expressed 2-fold higher. j. Expression patterns of *HNF4A, FOXA2*, and *SP8* calculated from the RNA-seq data. (N=) shows biologically independent experiments. Bar charts: (a), (f) mean, (j) FPKM. Scale bars: (d), (g), 50 µm. Source Data

As GATA6 and FGF4 efficiently induced hypoblast formation, we investigated the signalling pathways affected by *GATA6* overexpression that are vital for hypoblast induction. RNA-seq data showed the upregulation of *BMP2/6*, *STAT3*, *FRZB* and *FGFR2* and the downregulation of *WNT3* (Extended Data Fig. 3a). We therefore examined these signalling pathways using western blotting. While phosphorylated (p) SMAD1/5/9, pSTAT3 and pMAPK were upregulated, pSMAD2 was downregulated (Extended Data Fig. 3b). We therefore selected seven factors (7F) as candidates for chemical hypoblast specification: BMPs (a pSMAD1/5/9 activator), IL-6 (a pSTAT3 activator), FGF4, A83-01 (a pSMAD2 inhibitor and ALK4/5/7 inhibitor) and XAV939 (a WNT/β-catenin inhibitor and tankyrase inhibitor) along with PDGF-AA and retinoic acid, which work in mice for hypoblast specification^30–32^ (Fig. 2a). 7F induced the expression of PDGFRA and hypoblast genes in multiple naive hPSC cell lines (H9, H1, induced pluripotent stem cells (iPSCs)) but not in primed hPSCs (Fig. 2b,c, Extended Data Fig. 3c–g and Supplementary Fig. 1).

The transcriptome of naive 7F-PDGFRA^+^ cells was consistent with naive G6-PDGFRA^+^ cells (Extended Data Fig. 3h). A correlation analysis with human pre-implantation embryos^7^ revealed that they correlated most prominently (Fig. 2d). We concluded that naive hPSC-derived PDGFRA^+^ cells overexpressing *GATA6* or manipulated chemically to activate relevant signalling pathways progress into a hypoblast-like state, and we refer to these cells as nHyCs.

**Extended Data Fig. 4:**
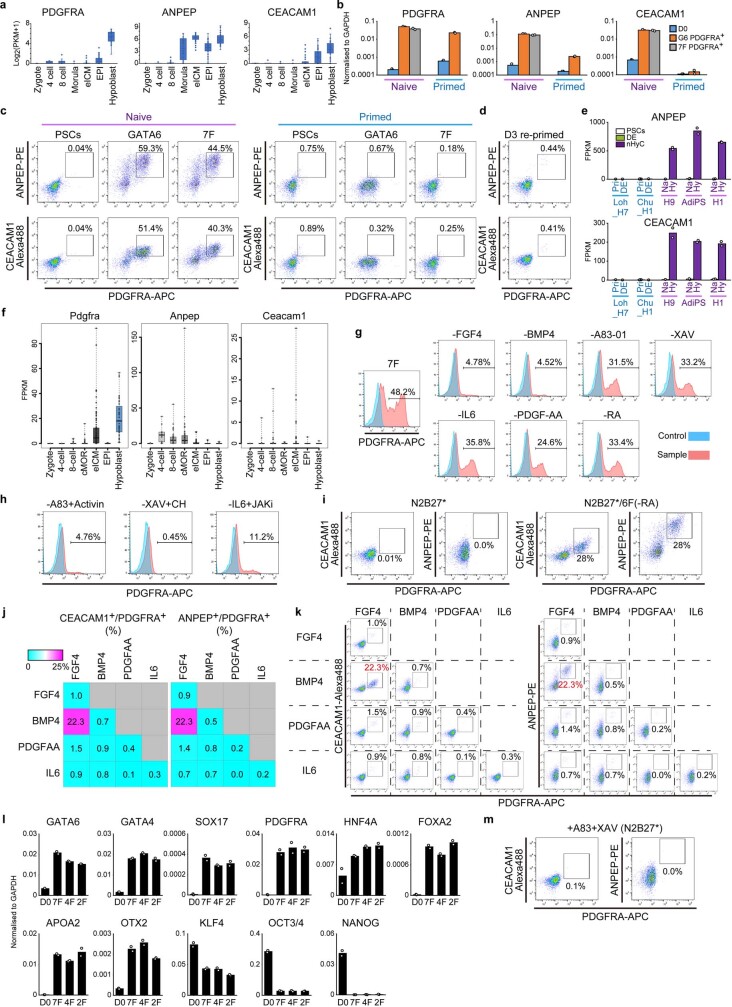
Surface markers and signalling molecules of human hypoblast cells. a. Gene expression patterns of *PDGFRA* and new hypoblast cell surface markers *ANPEP* and *CEACAM1* in pre-implantation human embryos. RNA-seq data from Stirparo et al. (2018)^7^ are shown as box-and-whisker plots. *Y*-axis shows Log_2_(FPKM + 1). zygote (3 cells), 4 cell (10 cells), eight cell (16 cells), morula (28 cells), ICM (43 cells), Epi (54 cells), hypoblast (38 cells). b. Expression patterns of *PDGFRA*, *ANPEP*, and *CEACAM1* in day D0 hPSCs and D3 PDGFRA^+^ cells measured by qPCR. *PDGFRA* was expressed in cells derived from naïve and primed hPSCs, *ANPEP* expression was 100-fold higher in naïve G6- and 7F-PDGFRA^+^ cells (nHyC) than in primed G6-PDGFRA^+^ cells, and *CEACAM1* was expressed only in G6- and 7F- PDGFRA^+^ cells (nHyC). Primed hPSCs and primed G6-PDGFRA^+^ cells did not express *CEACAM1*. N = 2. *Y*-axis shows log scale. c. Flow cytometry of ANPEP and CEACAM1 expression in PDGFRA^+^ cells from naïve hPSCs on D3. N = 2. d. Flow cytometry for PDGFRA, ANPEP, and CEACAM1 during the re-priming process. Naïve hPSCs were cultured in primed hPSC medium (Essential 8: E8^TM^) for 3 days. N = 2. e. ANPEP and CEACAM1 expression in definitive endoderm (DE) cells. DE cells and hPSCs do not express ANPEP or CEACAM1. RNA-seq data of primed hPSC-derived DE cells were reported by Loh et al. (2014)^71^ and Chu et al. (2016)^35^. Pri: primed hPSCs, Na: naïve hPSCs, Hy: nHyC. N = 2. f. *Pdgfra, Anpep*, and *Ceacam1* expression in mouse embryos. Dot plot images were obtained from the Genome-wide Rodent and Primate Preimplantation Atlas^7^. zygote (3 cells), 4 cell (12 cells), eight cell (36 cells), morula (49 cells), ICM (90 cells), Epi (19 cells), hypoblast (44 cells). g. PDGFRA expression in 7F minus one factor. Naïve hPSCs were cultured in 7F or 7F minus one factor. PDGFRA expression was measured on D3 by flow cytometry. Almost no PDGFRA expression was detected in cells cultured in 7F minus FGF4 or BMP4. 7F minus A83-01 (A83), XAV939 (XAV), IL6, PDGF-AA, or RA reduced the PDGFRA expression. N = 2. h. PDGFRA expression in 7F minus one factor plus one opposing factor. PDGFRA expression was measured on D3 by flow cytometry. 7F minus A83 or XAV but with Activin A or CHIR (CH) reduced the PDGFRA expression to around 0%, while 7F minus IL6 plus JAKi killed half of the cells during differentiation. Of the cells that survived, 11% expressed PDGFRA. N = 2. i. Flow cytometry of PDGFRA and CEACAM1 or ANPEP in cells cultured in 6F (-RA) medium. N2B27 medium without vitamin A (retinyl acetate) was used as the basal medium (N2B27*). N = 3. j. Minimum essential growth factors with A83 and XAV for hypoblast induction. Naïve hPSCs were cultured in growth factor(s) with A83 and XAV. A total of 22% of cells expressed CEACAM1, ANPEP, and PDGFRA when grown in FGF4 and BMP4 (4F). Colour blocks indicate the percentage of the protein expression. N = 2. k. Flow cytometry related to (j). l. qPCR for hypoblast markers in PDGFRA^+^ cells on D3 after 7F, 4F, or 2F treatment. N = 2. m. Flow cytometry for PDGFRA and CEACAM1 or ANPEP in cells treated with A83 and XAV. Signalling inhibition did not induce nHyC. N2B27*: N2B27 medium without vitamin A (retinyl acetate). N = 2. (N=) shows biologically independent experiments. Box plots: centre line, median; box, the 25th and 75th percentiles range; whiskers, (a) 1.5 × IQR; (f) the minimum and maximum values. Bar charts: (b), (e), (l) mean. Source Data

We identified that the transcription factors *FOXA2*, *HNF4A*, and *SP8* (Supplementary Table 3 and Extended Data Fig. 3i,j) and cell surface markers *ANPEP* (also known as CD13) and *CEACAM1* (Extended Data Fig. 4a,b) mark nHyCs. Flow cytometry confirmed that ANPEP and CEACAM1 were highly expressed in G6-nHyCs and 7F-nHyCs but not in naive hPSCs, primed cells, naive hPSCs in a primed medium (FGF2/TGFβ), definitive endoderm cells or mouse hypoblast (Extended Data Fig. 4c–f).

## FGF/BMP for hypoblast specification

During embryonic development, signalling pathways act in concert to promote specification. Accordingly, removing FGF4 or BMP4 from 7F medium substantially decreased PDGFRA expression (Extended Data Fig. 4g) and adding activin A or CHIR99021 abolished PDGFRA^+^ cells (Extended Data Fig. 4h). nHyCs were induced even when we removed vitamin A and retinoic acid (Extended Data Fig. 4i), suggesting that, contrary to the mouse hypoblast, the human hypoblast does not require retinoic acid for its specification. FGF4/BMP4 complemented with A83/XAV (4F) or without A83/XAV (2F), albeit at a low efficiency, successfully induced hypoblast gene expression and nHyCs (Fig. 2e and Extended Data Fig. 4j–m), which had strong correlations with hypoblasts of the blastocyst stage, similar to G6-nHyCs and 7F-nHyCs (Fig. 2d).

**Extended Data Fig. 5:**
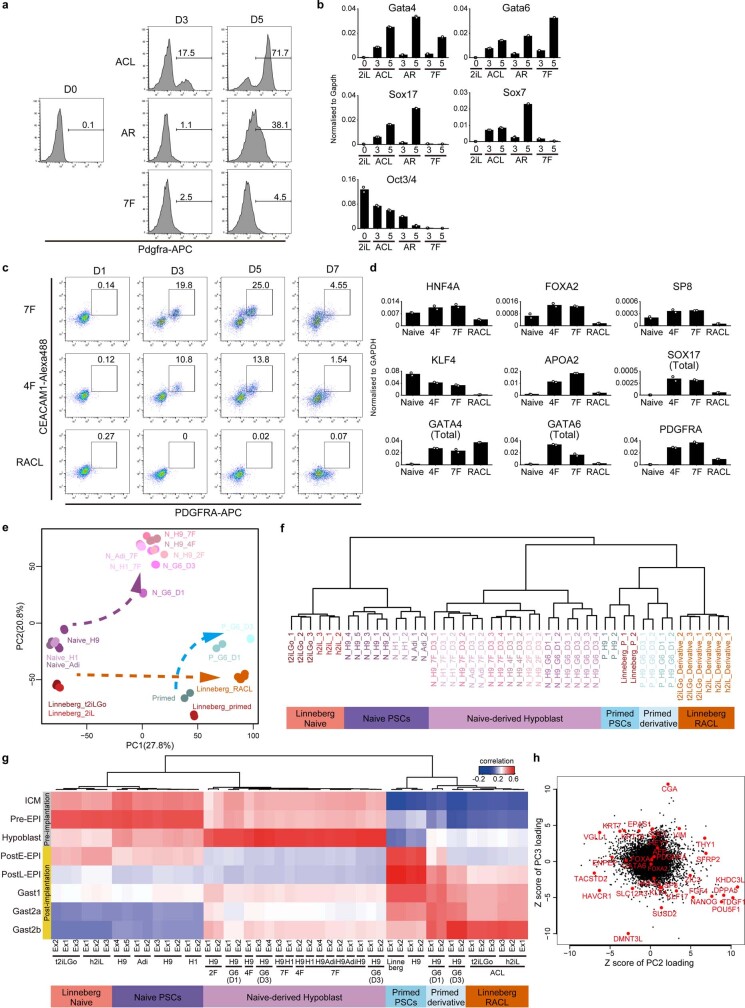
Signalling for hypoblast specification differs between humans and mice. a. PDGFRA expression in mouse ESCs with chemical induction was measured by flow cytometry. Mouse ESCs cultured in 2iL were treated with Activin A + CHIR99021 + LIF (ACL)^34^, Activin A + RA (AR)^30^, or 7F. N = 2. b. Hypoblast marker gene expression patterns in differentiated cells from mouse ESCs. Mouse ESCs cultured in 2iL at D0 were treated with ACL, AR, or 7F, and RNA was collected on D0, D3, and D5. *Sox7*, a mouse hypoblast marker, was upregulated on D5 by ACL or AR treatment but not by 7F. N = 2. c. Kinetics of PDGFRA and CEACAM1 expression in naïve hPSCs induced by 7F/4F or RACL. Hypoblast markers PDGFRA and CEACAM1 were expressed on D3 and D5 in 7F/4F-induced cells. Only PDGFRA was expressed on D7 in RACL-induced cells. N = 2. d. Gene expression patterns of PDGFRA^+^ cells in 7F/4F nHyC on D3 and RACL cells on D7. PDGFRA^+^ cells were sorted on D3 (7F/4F) or D7 (RACL). Gene expression levels were measured by qPCR. N = 2. e. Principal component analysis (PCA) of RNA-seq data from this study (7F/4F/2F- and G6-PDGFRA^+^ cells) and Linneberg et al. (2019)^23^. RNA-seq data (GSE138012)^23^ are indicated: naïve hPSCs, Linneberg_t2iLGo or Linneberg_2iL; hypoblast-like cells, Linneberg_RACL; primed hPSCs, Linneberg_primed. All other cell types are from this study. f. Unsupervised hierarchical clustering (UHC) of transcriptomes using RNA-seq data from this study and Linneberg et al.^23^. Cells cultured in RACL from Linneberg et al. clustered with primed hPSC-derived cells. g. Correlation coefficients for cynomolgus embryos and human cells from this study and Linneberg et al. (2019)^23^ based on 719 common ontogenic genes reported by Nakamura et al. (2016)^29^ (Supplementary Table 3). Cells from cynomolgus embryos were classified as ICM, pre-implantation Epi (Pre-EPI), hypoblast, post-implantation Epi (early stage: PostE-EPI, late-stage: PostL-EPI), or gastrulating cells (early: Gast1, middle: Gast2a, late: Gast2b). The colour code is the same as in (f). h. Scatter plot of the normalized loading scores of the PCA in Fig. 2h. Key genes are annotated. The distribution indicated that PC2 described the cell type (trophoblast and Epi), while PC3 described the development (from pre-implantation to post-implantation) (Fig. 2h). (N=) shows biologically independent experiments. Bar charts: (b), (d) mean. Source Data

To assess the effects of these molecules on hypoblast specification directly from the ICM of blastocysts, non-human-primate common marmoset ICM was cultured using 7F or 4F medium, or inhibitors of the FGF/BMP pathways (PD0325901/LDN-193189) and A83/XAV as a control (Fig. 2f). On day 3 of culture, the 4F colonies were flatter and contained larger cuboidal cells (Fig. 2f). SOX17^+^ hypoblast-like cells formed in 4F and 7F medium but not in the control medium (Fig. 2g). These observations suggest a crucial role for BMP/FGF signalling in hypoblast specification from the marmoset ICM while, in mouse ES cells, 7F did not induce PDGFRA or *Sox7*^33^, in contrast to activin A + CHIR99021/LIF (ACL)^34^ or activin A + retinoic acid^30^ (Extended Data Fig. 5a,b). These data indicate that, in contrast to transcription factors of which the hierarchy and functions appear to be conserved between humans and mice, signalling may be common between humans and marmosets but differs with mice.

Human hypoblast lineage cells are reported to be induced from naive hPSCs in RPMI with ACL (RACL)^23^. RACL induced PDGFRA^+^ cells by day 7 but not some other hypoblast markers (that is, *CEACAM1*, *HNF4A*, *FOXA2*, *SP8*, *SOX17* or *KLF4*), in contrast to 7F-nHyCs and 4F-nHyCs (Extended Data Fig. 5c,d). The transcriptome of RACL cells^23^ appeared to be more like post-implantation-stage cells, like primed-derived cells (Extended Data Fig. 5e–g). Furthermore, while PCA combined with single-cell RNA-seq (scRNA-seq) data of human embryos^5^ indicated that nHyCs and hypoblasts had similar gene expression profiles, RACL and primed-derived cells had closer expression profiles with post-implantation cells^35^ (Fig. 2h and Extended Data Fig. 5h), suggesting that nHyCs closely resemble the pre-implantation, blastocyst-stage hypoblast, a tissue that supports the epiblast development.

## Generation of bilaminoids

**Fig. 3:**
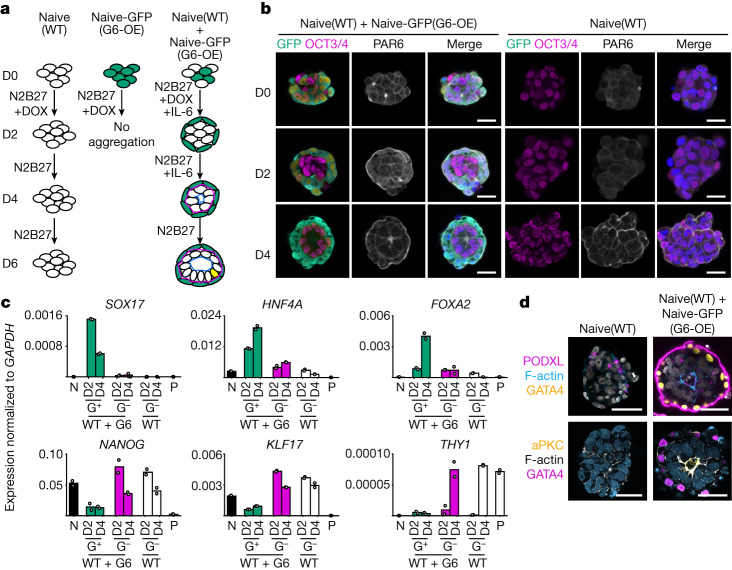
Naive hPSCs and nHyCs generate bilaminoids. **a**, Bilaminar embryo-like aggregates (bilaminoids) generated by the mixture of naive hPSCs and nHyCs. Aggregates were cultured without Matrigel. DOX was added for the first 2 days. IL-6 was added from day 0 to 4 where indicated. Naive(WT), WT naive hPSCs; Naive-GFP(G6-OE), GFP-expressing naive hPSCs expressing GATA6 under DOX treatment. **b**, Immunofluorescence images of cell aggregates. Green, Naive-GFP(G6-OE); purple, OCT3/4; white, PAR6; blue, DAPI. *n* = 3. **c**, qPCR analysis of aggregates on days 2 and 4. Cell aggregates were sorted by GFP on days 2 and 4. G^+^, GFP^+^; G^−^, GFP^−^; WT + G6-OE, mixed aggregates of Naive(WT) and Naive-GFP(G6-OE) cells; WT, aggregates of Naive(WT) cells only; N, naive hPSCs; P, primed hPSCs. *n* = 2. **d**, Immunofluorescence images of polarization markers in aggregates on day 4. Purple, PODXL; blue, F-actin; yellow, GATA4; white, DAPI (top); yellow, aPKC; white, F-actin; purple, GATA4; blue, DAPI (bottom). *n* = 2. *n* values show biologically independent experiments. Data are mean (**c**). Scale bars, 20 µm (**b**) and 50 µm (**d**). Source Data

During the peri-implantation period, non-polarized naive epiblast acquires apical–basal polarity, concomitantly loses naive pluripotency to create the pro-amniotic cavity and, finally, forms both the post-implantation epiblast and amnion cells. Meanwhile, the hypoblast differentiates into visceral endoderm and yolk sac endoderm cells. As the visceral endoderm and post-implantation epiblast, having lost the naive pluripotent state, generate the bilaminar disc together, we aimed to model their intertwined development by culturing naive hPSCs (naive, wild type (WT)) with naive hPSCs overexpressing *GATA6* under DOX treatment (Naive(G6-OE)) on a microwell array^36^ (Fig. 3a).

**Extended Data Fig. 6:**
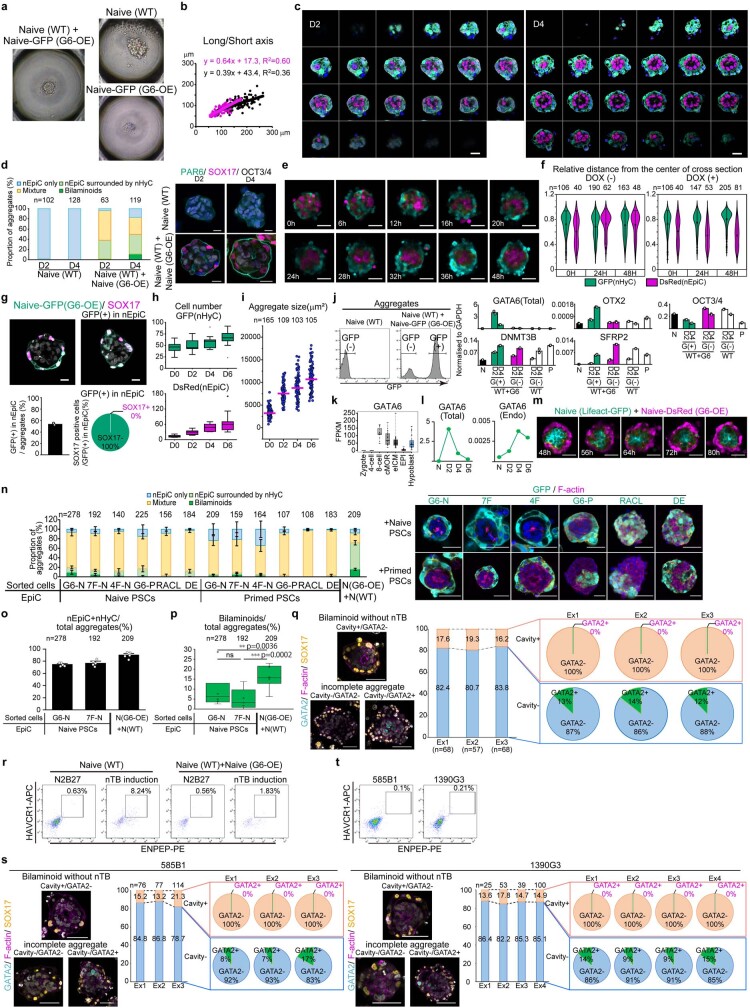
Bilaminoids generated by naïve hPSCs and nHyC. a. Representative images of cell aggregates consisting of Naïve (WT) (50 cells/well) only, Naïve-GFP (G6-OE) (50 cells/well) only, or Naïve (WT) (10 cells/well) and Naïve-GFP (G6-OE) (40 cells/well). N = 3. b. Ratios of the long axis to the short axis of cellular aggregates. The axes of aggregates generated by Naïve (WT) only or Naïve (WT) and Naïve-GFP (G6-OE) were measured on D4. The mixed aggregates of Naïve (WT) and Naïve-GFP (G6-OE) are more spherical than Naïve (WT) only. Purple, mixed aggregates; Black, Naïve (WT) only. N = 4. c. Representative series of Z-sections of Naïve (WT) and Naïve-GFP (G6-OE) aggregates on D2 and D4 stained for GFP (Green), OCT3/4 (Purple), and PAR6 (White). The interval is 3 μm. The number of images included is 23 (D2) and 24 slices (D4). N = 3. d. Efficiency of rosette formation on D2 and D4. Aggregates were composed of Naïve (WT) only or Naïve (WT) and Naïve (G6-OE). Green, PAR6; Purple, SOX17; White, OCT3/4; Blue, DAPI. N = 2. Representative images of aggregates are shown on the right. e. Time-lapse images of aggregates from 0 to 48 h after induction. nHyC(G6-OE) were in the periphery of the aggregates at 48 h. Green, nHyC(G6-OE); Purple, nEpiC. N = 3. f. Relative distance from the centre of aggregates. The distance of the nucleus of nHyC(G6-OE) and nEpiC from the centre of aggregates was measured at the level of the maximum cross-section for each aggregate. Ten aggregates were counted for each condition. N = 2. g. Distribution of Naïve-GFP (G6-OE) in bilaminoids on day 4. Although GFP-positive cells in nEpiC were observed, no GFP-positive cells in nEpiC expressed SOX17. Sixty-three aggregates on D4 were analysed. N = 2. h. Cell number of each aggregate on days 0, 2, 4, and 6. Ten aggregates on each day were counted. N = 2. i. Maximum cross-sectional area of each aggregate from D0 to D6. The size of each aggregate grew from D0 to D6. N = 2. j. Gene expression patterns in sorted cells from aggregates generated by Naïve (WT) only or Naïve (WT) and Naïve-GFP (G6-OE) on D2 and D4. G(+), GFP^+^ cells; G(−), GFP^-^ cells; WT + G6, mixed aggregates of Naïve (WT) and Naïve-GFP (G6-OE); WT, aggregates of Naïve (WT) only; N, naïve hPSCs; P, primed hPSCs. N = 2. k. *GATA6* expression in human pre-implantation embryos. Box plots were obtained from the Genome-wide Rodent and Primate Preimplantation Atlas^7^. zygote (3 Cells), 4 cell (10 cells), eight cell (16 cells), morula (28 cells), ICM (43 cells), Epi (54 cells), hypoblast (38 cells). l. *GATA6* expression in nHyC of the aggregates. Aggregates generated by Naïve (WT) and Naïve-GFP (G6-OE) were sorted on D2, D4, and D6. Total and endogenous expression (*GATA6* (Total) and *GATA6* (Endo), respectively) were measured by qPCR. N = 2. m. Time-lapse images of an aggregate from 48 h to 80 h after induction. Lifeact, a small peptide with an affinity for actin microfilaments (F-actin)^39^, was introduced into naïve hPSCs. Aggregates of Naïve-Lifeact (nEpiC) and Naïve-DsRed (G6-OE) (nHyC(G6-OE)) were cultured for 4 days. The time-lapse images show an accumulation of Lifeact in the centre starting at around 64 h. N = 2. n. Efficiency of bilaminoid generation. Aggregates were composed of naïve or primed hPSCs and naïve PDGFRA^+^ cells induced by *GATA6*, 7F, or 4F (7F/4F-, G6-nHyC), primed PDGFRA^+^ cells induced by *GATA6*, RACL cells, or ECAD^+^CXCR4^+^ definitive endoderm cells. For induction experiments, GFP-expressing cells were used. Aggregates were fixed and stained with F-actin (Purple) on D4. Although all sorted cell types surrounded Epi cells, aggregates with a pro-amniotic cavity were formed only by 7/4F- and G6-nHyC. Aggregates made by the mixture of Naïve (WT) and Naïve-GFP (G6-OE) formed pro-amniotic cavities most efficiently. Naïve and primed PDGFRA^+^ cells were sorted by PDGFRA on D3. RACL cells were sorted by PDGFRA on D7. Representative images of aggregates are shown on the right. G6-N, G6-nHyC; 7F-N, 7F-nHyC; 4F-N, 4F-nHyC; G6-P, primed G6-PDGFRA^+^ cells; RACL, PDGFRA^+^ RACL on D7; DE, definitive endoderm cells sorted by ECAD^+^CXCR4^+^ on D3. N = 6 (N + G6N), 5 (N + 7F), 4 (N + 4F), 5 (N + G6P), 4 (N + RACL), 4 (N + DE), 5 (P + G6N), 3 (P + 7F), 3 (P + 4F), 4 (P + G6P), 3 (P + RACL), 4 (P + DE), 6 (N(WT)+N(G6-OE)). o. Efficiency of bilaminoid formation on D4 extracted from (n). The efficiency of aggregates containing more than 10 nEpiC cells and 10 nHyC with spatial separation (inner-outer) is shown. N = 6(G6-N), 5 (7F-N), 6 (N(G6-OE)+N(WT)). p. Efficiency of bilaminoid formation on D4 extracted from (n). The efficiency of bilaminoids where nEpiC were surrounded by nHyC(G6-OE) and had an accumulation of F-actin on D4 is shown. N = 6(G6-N), 5 (7F-N), 6 (N(G6-OE)+N(WT)). Two-tailed Fisher’s exact test. q. Immunofluorescent images of aggregates on D6. Blue, GATA2; Purple, F-actin; Yellow, SOX17; White, DAPI. Epiblasts in incomplete aggregates without an amniotic cavity partially expressed GATA2, while there were no GATA2^+^ cells in bilaminoids. N = 3. r. Induction efficiency to HAVCR1^+^ENPEP^+^ trophoblast. Cell aggregates of H9 Naïve (WT) or Naïve (WT) + Naïve (G6-OE) were cultured under trophoblast induction medium for 3 days. N = 3. s. Immunofluorescent images of aggregates on D4. Blue, GATA2; Purple, F-actin; Yellow, SOX17; White, DAPI. Bilaminoids were induced from 558B1 or 1390G3 naïve hPSCs. Epiblasts in incomplete aggregates without an amniotic cavity partially expressed GATA2, while there were no GATA2^+^ cells in bilaminoids. N = 3 (585B1), N = 4 (1390G3). t. Induction efficiency to HAVCR1^+^ENPEP^+^ trophoblast. Cell aggregates of 585B1 or 1390G3 Naïve (WT) + Naïve (G6-OE) were cultured under trophoblast induction medium for 3 days. N = 3. (n=) at the top shows the number of aggregates analysed for each group. (N=) shows biologically independent experiments. Box plots: (h), (p) centre line, median; box, the 25th and 75th percentiles range; whiskers, 1.5 × IQR. (k) centre line, median; box, the 25th and 75th percentiles range; whiskers, the minimum and maximum values. Dot plot (i): purple bars, medians. Violin plots: (f) centre dot, mean; whiskers, 1.5 × IQR. Bar charts: (n), (o) mean ± SEM, (d), (g), (j), mean, (q), (s), percentage. Line charts: (l), mean. Scale bars: (c), (d), 20 µm; (e), (m), 40 µm; (n), (q), (s), 50 µm. Source Data

To mark aggregated cells, GFP or DsRed was introduced into naive hPSCs (Naive-GFP and Naive-DsRed, respectively). Aggregates generated by a mixture of Naive(WT) and Naive-GFP(G6-OE) cells were more spherical, consistent with the epithelial nature of hypoblast tissues (Extended Data Fig. 6a,b). While a mixture of Naive(WT) and Naive-GFP(G6-OE) cells was observed on day 0, Naive-GFP(G6-OE) after DOX treatment(called nHyCs(G6-OE)) relocated to the outer edge on day 2, as is typically observed in late blastocysts after maturation (Fig. 3b and Extended Data Fig. 6c), such that half of the aggregates were surrounded completely (Extended Data Fig. 6d). Time-lapse experiments confirmed the progressive segregation of GFP (nHyCs(G6-OE)) and DsRed (naive hPSC-derived epiblast-like cells (nEpiCs)) cells (Extended Data Fig. 6e,f). Only a few GFP^+^ cells were inside the aggregates on day 4 but were probably not hypoblast-like cells given their lack of SOX17 expression (Extended Data Fig. 6g). Previous reports of human embryos suggested that, between days 7 and 10, epiblast and hypoblast cell numbers increase from around 20–40 to 80–100 and from about 20–50 to 60–90, respectively^6,37,38^. Similarly, nHyC(G6-OE) and nEpiC cell numbers and aggregate size increased during differentiation (Extended Data Fig. 6h,i). *GATA6* total expression in nHyCs(G6-OE) on day 2 after DOX treatment, at a similar level to blastocysts^7^, was higher than in nEpiCs (Extended Data Fig. 6j,k). nHyCs(G6-OE) in day 2 aggregates upregulated hypoblast genes and downregulated pluripotency-related genes, whereas nEpiCs expressed naive or epiblast genes (Fig. 3c and Extended Data Fig. 6j,l). We therefore concluded that nHyCs and nEpiCs self-organize and express markers like the late human blastocysts.

We next analysed the apical–basal polarity of nEpiCs. Consistent with a blastocyst-like stage, PAR6 had not accumulated on day 2, (Fig. 3b). However, by day 4, around 20% of aggregates surrounded by nHyCs(G6-OE) accumulated PAR6 at the centre (Fig. 3b and Extended Data Fig. 6d). Polarized nEpiCs on day 4 gradually formed a rosette-like structure, which we refer to as bilaminoids, wherein PODXL and aPKC were localized together with F-actin (Fig. 3d). Lifeact—a small peptide with an affinity for actin microfilaments (F-actin)^39^—accumulated in the middle of the aggregates around 64 h after *GATA6* induction (Extended Data Fig. 6m). Consistent with a pre- to post-implantation transition, nEpiCs showed a gradual decrease in *KLF17* expression (naive pluripotency gene) and increases in *THY1*, *DNMT3B* and *SFRP2* expression (early post-implantation epiblast genes)^5,29,40^ (Fig. 3c and Extended Data Fig. 6j).

We also observed bilaminoids made by naive hPSCs and sorted naive PDGFRA^+^ cells induced by *GATA6*, 7F or 4F on laminin511-E8 (Extended Data Fig. 6n). Although primed G6-PDGFRA^+^ cells, RACL cells and definitive endoderm cells with either naive or primed hPSCs also surrounded epiblast cells, none generated a polarized cavity (Extended Data Fig. 6n). 7F-PDGFRA^+^ and G6-PDGFRA^+^ cells together with Naive(WT) cells generated bilaminoids with similar efficiency but less effectively compared with the mixture of Naive(WT) and Naive-GFP(G6-OE) cells, probably due to damages from flow cytometry (Extended Data Fig. 6p).

## Epiblast progression via TB-secreted IL-6

Naive hPSCs can differentiate into trophectoderm by blocking FGF and TGF-β/activin signalling pathways^21,22^ and can generate blastocyst-like structures (blastoids) under TB induction medium containing PD03 and A83^16,17^. Although we did not use PD03 and A83 for bilaminoid induction, we examined whether TBs appeared in the bilaminoids. Indeed, they were not found in bilaminoids, although a few GATA2^+^ cells were detected in incomplete aggregates without an amniotic cavity (Extended Data Fig. 6q). To quantify TB-like cells (nTBs), we performed flow cytometry and identified HAVCR1^+^ENPEP^+^ nTBs^4,21,22^. However, less than 1% were HAVCR1^+^ENPEP^+^ nTBs in bilaminoids, suggesting that they, in contrast to blastoids, do not contain TB-like cells (Extended Data Fig. 6r). These results were confirmed using two other independent iPSC lines (Extended Data Fig. 6s,t).

**Fig. 4:**
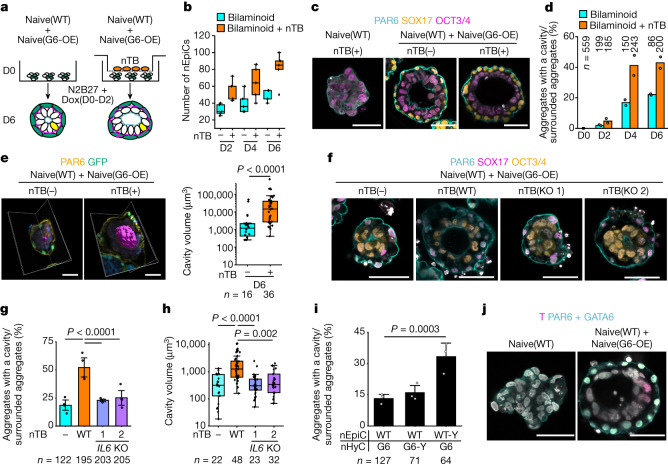
TB enhances epiblast progression through IL-6 paracrine signalling. **a**, Co-cultures of bilaminoids with naive hPSC-derived TBs (nTB) on Transwell plates. Co-cultures were performed from day 0 to day 4. **b**, The cell number of nEpiCs in each aggregate. Ten aggregates on each day were counted. *n* = 2 biologically independent experiments. **c**, Immunofluorescence images of aggregates on day 6. Blue, PAR6; yellow, SOX17; purple, OCT3/4; white, DAPI. *n* = 2 biologically independent experiments. **d**, The efficiency of cavity formation. *n* = 2 biologically independent experiments. **e**, Three-dimensional images and volume of the amniotic cavity in each aggregate on day 6. Purple, amniotic cavity; yellow, PAR6; green, naive-GFP(GATA6); blue, DAPI. *n* = 3 biologically independent experiments. Statistical analysis was performed using two-tailed Mann–Whitney’s *U*-tests. **f**, Immunofluorescence images of aggregates using *IL6-*KO naive hPSCs on day 6. Blue, PAR6; purple, SOX17; yellow, OCT3/4; white, DAPI. nTB(−), bilaminoid without nTB; nTB(WT), bilaminoid with nTB(WT); nTB(KO 1 or 2), bilaminoid with nTB (*IL6*-KO two clones (1 or 2)) (Extended Data Fig. 7j). *n* = 4 biologically independent experiments. **g**, The efficiency of cavity formation on day 6 as in **f**. *n* = 4 biologically independent experiments. Statistical analysis was performed using two-tailed Fisher’s exact tests. **h**, The volume of the amniotic cavity of each aggregate on day 6 as in **f**. *n* = 4 biologically independent experiments. Statistical analysis was performed using Kruskal–Wallis and Dunn’s multiple-comparisons test. **i**, The efficiency of cavity formation in bilaminoids after STAT3 activation on day 4. GP130/GCSFR chimeric gene (Y118F) activates STAT3 signalling by adding G-CSF. WT, Naive(WT); WT-Y, Naive(WT) with Y118F; G6, Naive(G6-OE); G6-Y, Naive(G6-OE) with Y118F. *n* = 3 biologically independent experiments. Two-tailed Fisher’s exact test. **j**, Immunofluorescence images of aggregates with nTB on day 6. Purple, T; blue, PAR6 and GATA6; white, DAPI. *n* = 5 biologically independent experiments. For **d**,**e** and **g**–**i**, the number of aggregates analysed for each group is shown. For the box plots in **b**,**e** and **h**, the centre line shows the median; the box limits show the 25th and 75th percentile range, and the whiskers show 1.5 × interquartile range (IQR). Data are mean ± s.e.m. (**g** and **i**) and mean (**d**). Scale bars, 50 µm (**c**,**e**,**f** and **j**). Source Data

**Extended Data Fig. 7:**
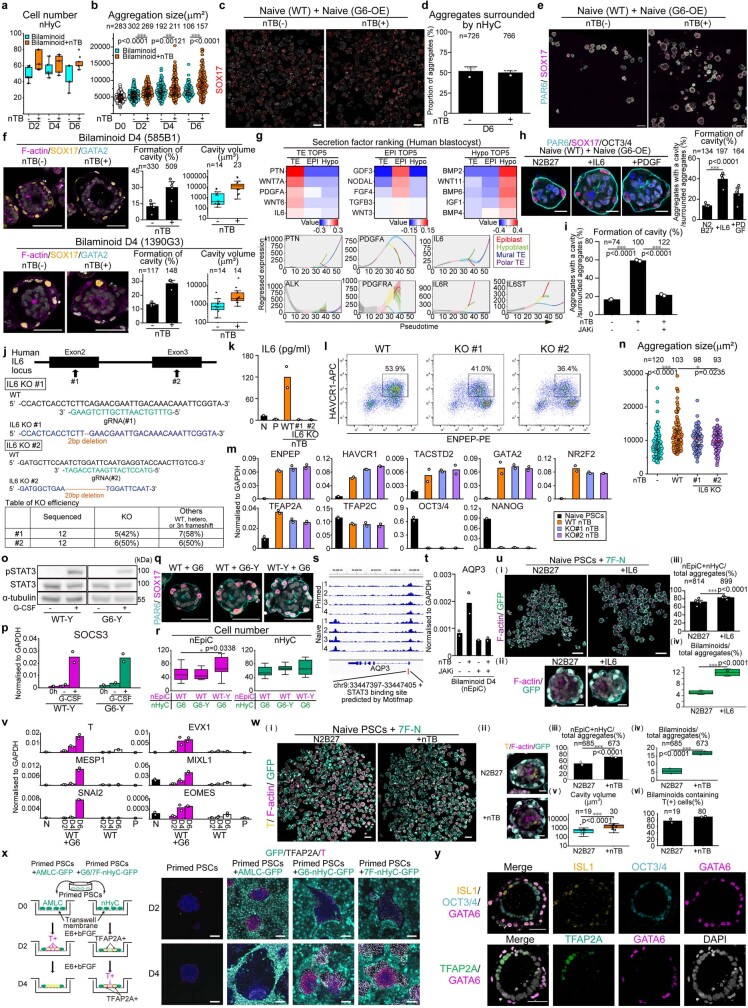
Trophoblast enhances epiblast progression. a. Cell number of nHyC in each aggregate on D2, D4, and D6. Ten aggregates on each day were counted. N = 2. b. Maximum cross-sectional area of bilaminoids and bilaminoids + nTBs from D0 to D6. N = 2. Two-tailed Mann-Whitney’s U test. c. Immunofluorescent images of aggregates with or without nTB on D6. Red, SOX17; White, DAPI. N = 3. d. Proportion of aggregates surrounded by nHyC, generated with or without nTB, on D6. N = 3. e. Immunofluorescent images of aggregates surrounded by nHyC with or without nTB on D6. Aggregates surrounded by nHyC were collected under a stereomicroscope. Blue, PAR6; Purple, SOX17; White, DAPI. N = 3. f. Immunofluorescent images, efficiency of cavity formation, and volume of the amniotic cavity of aggregates on D4 generated by two iPS cell lines, 585B1 and 1390G3. Purple, F-actin; Yellow, SOX17; Blue, GATA2; White, DAPI. N = 5 (585B1), N = 4 (1390G3). g. Heatmap of the top 5 secreted factors of TE, EPI, and Hypo in human embryos^14^ (upper). Pseudotime expression^41^ of secreted factors and receptors from TE-specific factors identified in the upper panel (lower). h. Bilaminoids on D4 after IL6 or PDGF-AA treatment. Cavity formation rate (right). Sky blue, PAR6; Purple, SOX17; White, OCT3/4; Blue, DAPI. N = 5. Two-tailed Fisher’s exact test. i. Cavity formation rate of bilaminoids on D4 under JAK/STAT signalling pathway inhibition. N = 2. Two-tailed Fisher’s exact test. j. Design of the CRISPR targets for *IL6* knockout (KO). Two different Cas9 sgRNAs targeting *IL6* (#1 and #2) were separately transfected to primed hPSCs. CRISPR target sites and DNA sequences of both alleles are shown. The table shows the efficiency of homozygous *IL6* KO. k. Secretion of human IL6. nTBs were generated from naïve hPSCs (WT, IL6KO#1, and IL6KO#2). IL6 secreted by nTBs into the cultured medium was measured by ELISA. N = 2. l. Trophoblast induction on D3 from naïve hPSCs (WT, IL6KO#1, and IL6KO#2). N = 3. m. qPCR analysis in HAVCR1^+^ ENPEP^+^ cells of naïve hPSC (WT, IL6KO#1, and IL6KO#2) 3 days after trophoblast induction. N = 2. n. Maximum cross-sectional area of each aggregate on D4. Bilaminoids were generated without nTB, with nTB (WT) or nTB (IL6KO#1, #2). N = 2. Kruskal-Wallis and Dunn’s multiple comparisons test. *p* value, adjusted. o. Western blot showing STAT3 phosphorylation after G-CSF treatment. GP130/GCSFR chimaeric gene (Y118F), which activates STAT3 signalling in the presence of G-CSF, was introduced into Naïve (WT) or Naïve (G6-OE). Protein was collected 2 h after G-CSF treatment of naïve hPSCs. N = 2. p. SOCS3 expression after G-CSF treatment. RNA was collected 2 h after G-CSF treatment of naïve hPSCs. N = 2. q. Representative immunofluorescent images of aggregates on D4. Aggregates were generated by Naïve (WT) + Naïve (G6-OE), Naïve (WT) + Naïve (G6-OE-Y118F(G6-Y)), or Naïve (WT-Y118F(WT-Y)) + Naïve (G6-OE). N = 3. r. Cell numbers of nEpiC and nHyC in each aggregate on D4. Ten aggregates of each condition were counted. N = 2. One-way ANOVA and Dunnett’s multiple comparisons test. *p* value, adjusted, 12 aggregates (WT + G6), 13 aggregates (WT + G6-Y), 14 aggregates (WT-Y + G6). s. ATAC-seq signal of naïve hPSCs and primed hPSCs at the *AQP3* locus. A STAT3 binding motif was previously predicted in this locus^73^. ATAC-seq data was obtained from Pastor et al.^72^. t. qPCR analysis for nEpiC in bilaminoids on D 4. nEpiC in bilaminoids, with or without nTB, were sorted as GFP^-^ on D4. *AQP3* expression was measured. N = 2. u. Bilaminoids generated by naïve hPSCs and G6- or 7F-PDGFRA^+^ cells (G6-N or 7F-N) after IL6 treatment on D4. (i, ii) Low- and high-magnification immunofluorescent images of aggregates on D4. (iii) The efficiency of bilaminoid formation calculated using the alternative criteria, in which an aggregate containing more than 10 epiblast-like cells surrounded by more than 10 hypoblast-like cells are defined as bilaminoids. N = 6. (iv) The efficiency of bilaminoid formation analysed with the stringent criteria, in which a bilaminoid is defined as an aggregate surrounded completely by nHyC in a single complete amniotic-like cavity. Purple, F-actin; Green, nHyC; White, DAPI. N = 6. Two-tailed Fisher’s exact test (iii, iv). v. qPCR analysis for aggregates on D2, D4, and D6. nEpiC in cell aggregates were sorted as GFP^-^ on D2, D4, and D6. WT + G6, mixed aggregates of Naïve (WT) and Naïve-GFP (G6-OE); WT, aggregates of Naïve (WT) only; N, naïve hPSCs; P, primed hPSCs. N = 2. w. Bilaminoids generated by naïve PSCs and 7F-PDGFRA^+^ cells (7F-N) after nTB treatment on D6. (i, ii) Low- and high-magnification immunofluorescent images of aggregates on D6. (iii) The efficiency of bilaminoid formation calculated using the alternative criteria, in which an aggregate containing more than 10 epiblast-like cells surrounded by more than 10 hypoblast-like cells are defined as bilaminoids. (iv) The efficiency of bilaminoid formation analysed with the stringent criteria, in which a bilaminoid is defined as an aggregate surrounded completely by nHyC in a single complete amniotic-like cavity. (v) The cavity volume of bilaminoids with or without nTB. (vi) T-positive cells in bilaminoids with or without nTB. Yellow, T; Purple, F-actin; Green, nHyC; White, DAPI. N = 2. Two-tailed Fisher’s exact test (iii, iv) and Two-tailed Mann-Whitney’s U test (v). x. Transwell co-culture assay. Primed hPSCs only, primed hPSCs + amniotic ectoderm cells^4^, and primed hPSCs + nHyC (G6 or 7F) were cultured on Transwell plates for 4 days. Green, amniotic ectoderm or nHyC; White, TFAP2A; Purple, T; Blue, DAPI. N = 2. y. Immunofluorescent images for amnion markers of bilaminoids on D6. Yellow, ISL1; Blue, OCT3/4; Green, TFAP2A; Purple, GATA6; White, DAPI. Aggregates were generated by Naïve (WT) + Naïve (G6-OE) together with nTB. N = 4. (n=) at the top shows the number of aggregates analysed for each group. (N=) shows biologically independent experiments. Box plots: (a), (f), (r), u(iv), (w(iv)), (w(v)) centre line, median; box, the 25th and 75th percentiles range; whiskers, 1.5 × IQR. Dot plots: (b), (n) red bars, medians. Bar charts: (d), (f), (h), (u(iii)) mean ± SEM, (i), (k), (m), (p), (t), (v), (w(iii)), (w(vi)) mean. Scale bars: (c), (e), (u(i)), (w(i)), 200 µm; (f), (q), (y), 50 µm; (h), (u(ii)), (w(ii)), (x), 20 µm. Source Data

We next analysed the role of TBs in epiblast development by co-culturing Naive(WT) + Naive(G6-OE) with nTBs that were separately cultured on a Transwell plate (Fig. 4a). nEpiC proliferation was enhanced in the presence of nTBs (Fig. 4b and Extended Data Fig. 7a), resulting in larger bilaminoids (Extended Data Fig. 7b). Although the efficiency of generating aggregates surrounded by nHyCs was similar for bilaminoids with and without nTBs (around 50%; Extended Data Fig. 7c,d), the amniotic cavity formed more efficiently and to a larger size with nTBs (from 20% to 40%; Fig. 4c–e and Extended Data Fig. 7e). This effect was confirmed using two other naive hPSC lines (Extended Data Fig. 7f).

As bilaminoids and nTBs were separately cultured, we hypothesized that TBs promote epiblast proliferation and accelerate pro-amniotic cavity formation through secreted factors. As previously reported, IL-6 and PDGFA are expressed in TBs^41^ (Extended Data Fig. 7g). When IL-6 or PDGFA were added to the culture of bilaminoids, they efficiently enhanced pro-amniotic cavity formation by day 4 (Extended Data Fig. 7h). Furthermore, JAK inhibitor treatment negated the positive effects of nTBs (Extended Data Fig. 7i). We next knocked out *IL6* in naive hPSCs (Extended Data Fig. 7j,k). *IL6*-knockout (KO) naive hPSCs differentiated into trophectoderm (Extended Data Fig. 7l,m), but these cells did not enhance bilaminoid growth and cavitation (Fig. 4f–h and Extended Data Fig. 7n). Finally, to determine whether IL-6 acts on nEpiCs or nHyCs, we activated JAK/STAT3 signalling in nEpiCs or nHyCs using the GP130/GCSFR chimeric receptor (Y118F)^42^. Both cell types activated STAT3 signalling (Extended Data Fig. 7o,p) but bilaminoids formed pro-amniotic cavities more efficiently by day 4 when STAT3 signalling was specifically activated in the nEpiCs (Fig. 4i and Extended Data Fig. 7q,r). We concluded that nTB-secreted IL-6 activates STAT3 signalling in nEpiCs to support proliferation and pro-amniotic-like cavity formation. This positive effect by IL-6 was also observed in the bilaminoids generated by 7F-nHyCs (Extended Data Fig. 7u).

## Mesoderm-like cells emerge in bilaminoids

After forming the pro-amniotic cavity and bilaminar disc, a subset of epiblast cells engages in gastrulation. By day 6, nEpiCs surrounded by nHyCs expressed *TBXT *(*T*) and primitive-streak-related genes (Fig. 4j and Extended Data Fig. 7v). Importantly, without nHyCs, cavities did not form, and mesoderm genes were not induced even in the presence of IL-6 and nTBs (Fig. 4j and Extended Data Fig. 7v). By contrast, the aggregates surrounded by 7F-nHyCs also contained cavities and T^+^ cells at day 6 (Extended Data Fig. 7w). Moreover, nTBs increased the efficiency of bilaminoids generated by 7F-nHyCs and the pro-amniotic cavity volume (Extended Data Fig. 7w). To induce mesoderm, the amniotic ectoderm is essential in human^12^. Co-culturing with G6- or 7F-nHyCs on Transwell plates, we confirmed that primed hPSCs to differentiate into T^+^ mesoderm cells 2 days after amnion-like cells emerged (Extended Data Fig. 7x). Furthermore, we observed GATA3^+^, TFAP2A^+^ or ISL1^+^ cells (amnion markers) in day 6 bilaminoids (Extended Data Fig. 7y). We concluded that nHyCs have a crucial role in regulating the expression of gastrulation-related genes in nEpiCs.

## Single-cell transcriptomics of bilaminoids

**Fig. 5:**
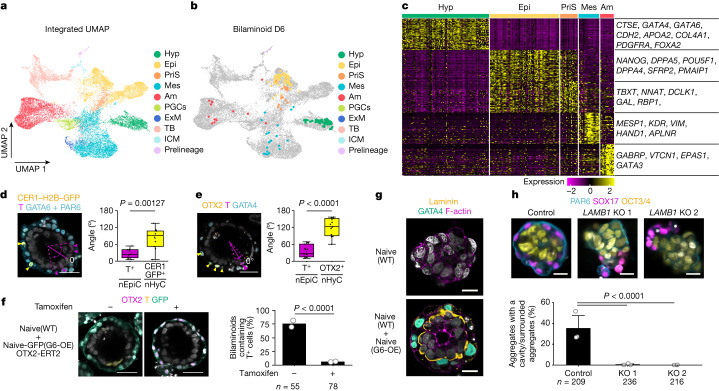
Global gene expression profiles of individual cells in bilaminoids. **a**, UMAP analysis of integrated datasets of bilaminoids, published human embryos^2,3,5,8,16^ and stem-cell-based embryo models^12,14–17^ (Extended Data Fig. 8b–d). PriS, primitive streak; Mes, mesoderm; Am, amnion; ExM, extraembryonic mesoderm. **b**, Cells from day 6 bilaminoids highlighted on the UMAP shown in **a**. **c**, The relative expression values of the top 50 differentially expressed genes of bilaminoids and representative genes. **d**, The anterior–posterior axis of bilaminoids on day 6. Yellow, CER1–H2B–GFP (nuclei); purple, T; blue, GATA6 + PAR6; white, DAPI. Yellow and purple arrowheads indicate CER1–H2B–GFP^+^ and T^+^ nuclei, respectively. Ten aggregates expressing T and H2B–GFP were analysed. *n* = 3 biologically independent experiments. Statistical analysis was performed using two-tailed Welch’s *t*-tests. **e**, Anterior–posterior axis of bilaminoids on day 6. Yellow, OTX2; purple, T; blue, GATA4; white, DAPI. The yellow and purple arrowheads indicate OTX2^+^ and T^+^ nuclei, respectively. A total of 14 aggregates expressing T and OTX2 was analysed. *n* = 3 biologically independent experiments. Statistical analysis was performed using two-tailed Mann–Whitney *U*-tests. **f**, *OTX2* overexpression in nHyCs. Bilaminoids were generated by Naive(WT) + Naive-GFP(G6-OE) cells containing tamoxifen-inducible *OTX2* (*OTX2-ERT2*). Purple, OTX2; yellow, T; green, GATA6; white, DAPI. *n* = 2 biologically independent experiments. Statistical analysis was performed using two-tailed Fisher’s exact tests. **g**, Immunofluorescence images of LAMININ in bilaminoids on day 4. Yellow, laminin; green, GATA4; purple, F-actin; white, DAPI. *n* = 2 biologically independent experiments. **h**, Aggregates generated by Naive(WT) and Naive *LAMB1* KO(G6-OE) (two clones (1 and 2)) cells (Extended Data Fig. 9c). Blue, PAR6; purple, SOX17; yellow, OCT3/4; white, DAPI. *n* = 3 biologically independent experiments. Statistical analysis was performed using two-tailed Fisher’s exact tests. For **f** and **h**, the number of aggregates analysed for each group is shown at the bottom. For the box plots in **d** and **e**, the centre line shows the median; the box limits show the 25th and 75th percentile range, and the whiskers show 1.5 × IQR. Data are mean ± s.e.m. (**h**) and mean (**f**). Scale bars, 50 µm (**d**–**f**) and 20 µm (**g** and **h**). Source Data

**Extended Data Fig. 8:**
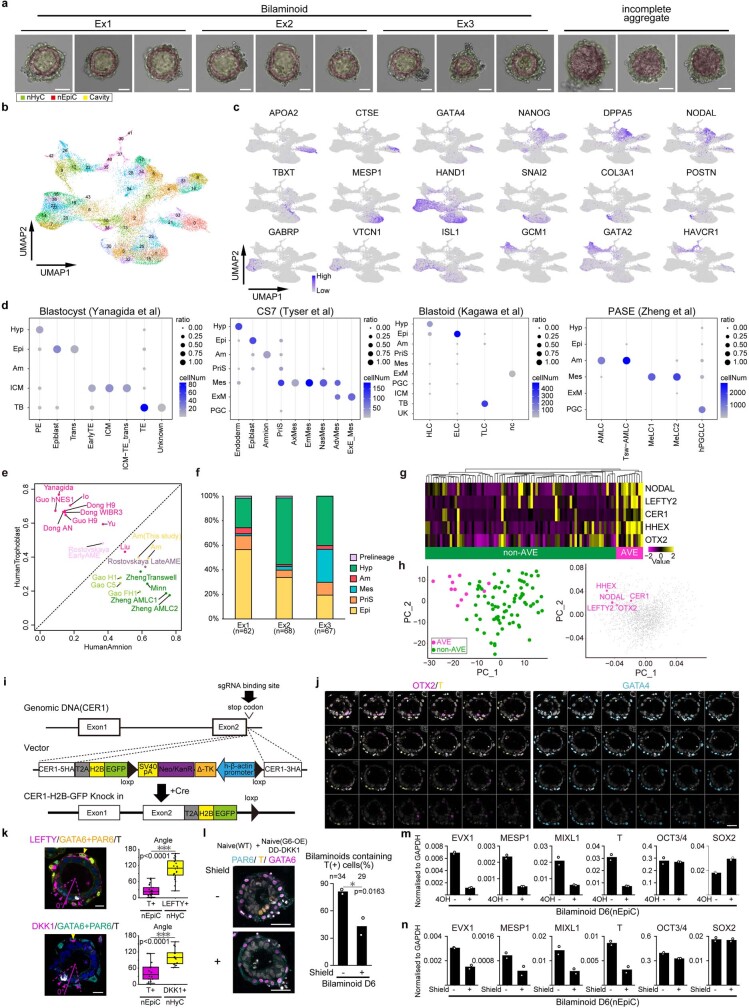
Global gene expression profiles and anterior-posterior axis formation of bilaminoids. a. Representative bright field images of bilaminoids for Smart-seq. Hypoblast, epiblast, and the cavity are colour-coded: nHyC, green; nEpiC, red; Cavity, yellow. N = 3. b. Uniform Manifold Approximation and Projection (UMAP) of integrated data sets of 215 single cells from 23 bilaminoids, 5 published human embryos^2,3,5,8,16^ and 5 stem cell-based embryo models^12,14–17^. Resolution:2.8. c. Expression of lineage marker genes. The gene expression levels are plotted on UMAP and shown as feature plots. d. Dot plot showing our annotations and the reported annotations from human blastocyst data^16^, Carnegie stage 7 (CS7) post-implantation human embryo data^8^, human blastoid data^17^, and amniotic sac embryoid (PASE) model^12^. The sizes and colours of dots indicate the proportion and number of cells annotated to the corresponding cell types, respectively. e. Correlation coefficients of *in vitro* induced cells with trophoblast and amniotic ectoderm of human embryos. The amnion of D6 bilaminoids and amnion cluster of this study highly correlated to amniotic ectoderm. Cell types and ontogenic genes between amniotic ectoderm and trophoblast were previously reported^66^. f. Percentage of each cell type in bilaminoids on D6. Six, eight, and nine bilaminoids were independently collected and analysed by Smart-seq. Hyp, hypoblast; Epi, epiblast; PriS, primitive streak; Mes, mesoderm; Am, amnion. N = 3. g. Unsupervised hierarchical clustering (UHC) of nHyCs of D6 bilaminoids and relative gene expression of anterior visceral endoderm (AVE) marker genes. One cluster of nHyC strongly expresses AVE marker genes. h. PCA analysis of nHyCs of D6 bilaminoids (Left) and loadings (right). AVE marker genes are labelled. i. Design of the CRISPR targets for CER1-H2B-GFP knock-in (KI). The neo cassette was removed by Cre expression. j. Representative Z-series images of an aggregate on D6. A series of confocal Z-sections of the aggregates of Naïve (WT) + Naïve (G6-OE) on D6 stained for OTX2 (Purple), T (Yellow), GATA4 (Blue), and DAPI (White). N = 3. k. Anterior-posterior axis of D6 bilaminoids. Bilaminoids were generated by Naïve (WT) + Naïve (G6-OE). Purple, LEFTY, DKK1; Yellow or Green, GATA6 + PAR6; White, T; Blue, DAPI. Purple arrowheads indicate T^+^-nucleus. Yellow arrowheads indicate LEFTY^+^ or DKK1^+^ cells. Angles between T^+^ nuclei and LEFTY^+^ or DKK1^+^ cells on sections of bilaminoids were measured. The centre of T^+^ nuclei was defined as 0 degrees. LEFTY: 21 aggregates surrounded by nHyC (G6-OE) and expressing LEFTY and T were counted. N = 2. DKK1: 14 aggregates surrounded by nHyC (G6-OE) and expressing DKK1 and T were counted. N = 2. Two-tailed Mann-Whitney’s U test (LEFTY), Two-tailed *t*-test (DKK1). l. Overexpression of DKK1 in Naïve (G6-OE) suppressed T expression in the nEpiC of D6 bilaminoids. DKK1 with Destabilized domain (DD-DKK1) was introduced into Naïve (G6-OE) and was stabilized by Shield1. Bilaminoids were generated by Naïve (WT) + Naïve-GFP (G6-OE) that contain DD-DKK1. Blue, PAR6; Yellow, T; Purple, GATA6; White, DAPI. The proportion of T-expressing bilaminoids was counted under Shield1 (−) or (+) conditions. N = 2. Two-tailed Fisher’s exact test. m. qPCR analysis of D6 bilaminoids. Bilaminoids were generated by Naïve (WT) + Naïve-GFP (G6-OE) that contain tamoxifen-inducible OTX2 (OTX2-ERT2). RNA was collected under tamoxifen (−) or (+) conditions. 4OH (−) or (+), tamoxifen (−) or (+). N = 2. n. qPCR analysis of D6 bilaminoids. Bilaminoids were generated as (l). RNA was collected under Shield1 (−) or (+). N = 2. (n=) at the top shows the number of aggregates analysed for each group. (N=) shows biologically independent experiments. Box plots: (k) centre line, median; box, the 25th and 75th percentiles range; whiskers, 1.5 × IQR. Bar charts: (l), (m), (n) mean, (f) percentage. Scale bars: (a), (j), (l), 50 µm; (k), 20 µm. Source Data

We identified the cell types of bilaminoids using scRNA-seq (197 cells from 23 bilaminoids; Extended Data Fig. 8a and Supplementary Table 4) and benchmarked them against a reference human embryo dataset^2,3,5,8,16^ together with recently published human embryo models^12,14–17^. We generated an integrated uniform manifold approximation and projection (UMAP), as proposed previously^43^, which clustered each cell type of the embryos as hypoblast, epiblast, primitive streak, mesoderm, amnion, primordial germ cells (PGCs), extraembryonic mesoderm, TB and ICM (Fig. 5a and Extended Data Fig. 8b,c). We confirmed that our clusters match with reported annotations of embryos and embryo models (Extended Data Fig. 8d). As TBs and amnion cells share many common genes, we further analysed whether our clustering separated them properly. We observed that the amnion cell clusters correlate with the amnion strongly but not with the trophectoderm (Extended Data Fig. 8e and Supplementary Table 5). Finally, we checked the annotation of our bilaminoids in this integrated UMAP (Fig. 5b and Extended Data Fig. 8f). Hypoblast, epiblast, primitive streak, mesoderm and amnion cells were reproducibly present on day 6, whereas TB cells were not. Each cluster expressed key cell-type-specific marker genes (Fig. 5c and Supplementary Table 6). Notably, we noticed that a subpopulation of nHyCs in bilaminoids classified by UHC expressed anterior visceral endoderm marker genes (Extended Data Fig. 8g). PCA and contributed genes also suggested that there were anterior-visceral-endoderm-like cells in the bilaminoids on day 6 (Extended Data Fig. 8h).

## Anterior–posterior axis formation in bilaminoids

During mouse embryogenesis, a subpopulation of hypoblasts secretes anteriorization factors to guide anterior–posterior axis formation by restricting gastrulation to the posterior epiblasts^44^. To track CER1 expression, one of the anteriorization factors, we generated *CER1-H2B-GFP* knockin naive hPSCs (Extended Data Fig. 8i). We detected CER1–H2B–GFP^+^ cells in a part of the nHyC(G6-OE) bilaminoids on day 6 and T^+^ cells located away from them in nEpiCs (Fig. 5d). Similarly, T^+^ cells did not contact OTX2^+^ cells in nHyCs, which also marks the anterior visceral endoderm (Fig. 5e and Extended Data Fig. 8j). Further immunostaining of the anterior visceral endoderm markers DKK1 and LEFTY confirmed this positional information (Extended Data Fig. 8k). To check whether anterior visceral marker genes were functional, we overexpressed *OTX2* or *DKK1* in nHyCs, which reduced T expression along with other mesoderm genes (Fig. 5f and Extended Data Fig. 8l–n), indicating that nHyCs control anterior–posterior axis formation and patterns epiblast differentiation. We further concluded that a subpopulation of nHyCs inhibits and thereby patterns the expression of gastrulation-related genes in nEpiCs.

## nHyCs support epiblast progression

**Extended Data Fig. 9:**
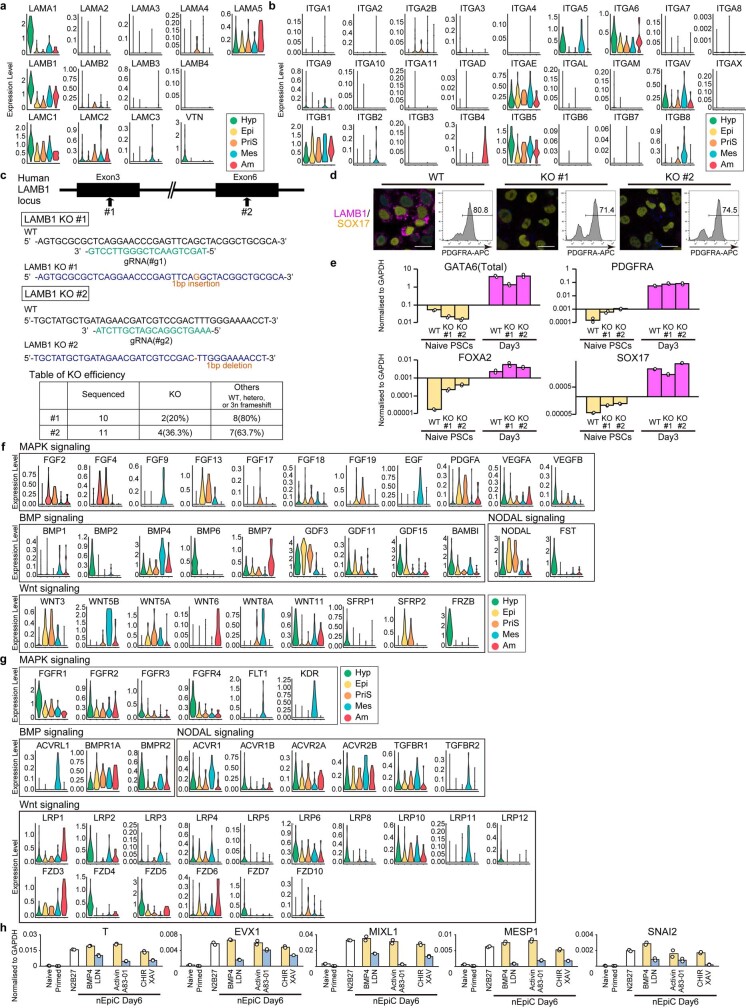
*LAMB1* knockout nHyC and gene expression profiles of bilaminoids. a. Expression of integrins in each cluster (Fig. 5b). Only cells from bilaminoids were extracted and analysed. Expression levels are shown as violin plots. b. Expression of laminins in each cluster. Only cells from bilaminoids were extracted and analysed. Expression levels are shown as violin plots. c. Design of the CRISPR targets for *LAMB1* knockout (KO). Two different sgRNA with CAS9 for *LAMB1* (#1 and #2) were separately transfected to primed hPSCs. CRISPR target sites and DNA sequences of both alleles for the indicated knockout lines are shown. #1 line has a 1 bp insertion, and #2 line has a 1 bp deletion. The table at the bottom shows the efficiency of homozygous *LAMB1* KO. KO was confirmed as biallelic frame-shift nonsense mutations. d. Verification of the loss of LAMB1 expression by immunofluorescence. *LAMB1* KO #1 and #2 primed hPSCs containing DOX-inducible *GATA6* were reset to naïve hPSCs (Naïve LAMB1KO-hPSC(G6-OE)). The loss of LAMB1 was confirmed after DOX induction on D3. Purple, LAMB1; Yellow, SOX17; Blue, DAPI. Expression of PDGFRA on D3 after *GATA6* overexpression. Naïve LAMB1KO-hPSC(G6-OE) were cultured under N2B27 medium as in Extended Data Fig. 1o. PDGFRA expression was measured by flow cytometry on D3. N = 2. e. Gene expression patterns of PDGFRA^+^ cells. Hypoblast marker genes in PDGFRA^+^ cells on D3 were measured by qPCR. N = 2. *Y*-axis shows log scale. f. Expression of secreted factors. Only cells from bilaminoids were extracted and analysed. Expression levels are shown as violin plots. g. Violin plots of receptor expression. Only cells from bilaminoids were extracted and analysed. Expression levels are shown as violin plots. h. Gene expression patterns in nEpiC of bilaminoids cultured under N2B27 medium with BMP, ACTIVIN, or WNT signalling ligands or inhibitors on D6. Bilaminoids were generated by the mixture of Naïve (WT) and Naïve-GFP (G6-OE). A growth factor or inhibitor to BMP, ACTIVIN, or WNT signalling was added from D4 to D6. GFP^-^ nEpiC were sorted on D6. N = 2. (N=) shows biologically independent experiments. Bar charts: (e), (h) mean. Scale bars: (d), 20 µm. Source Data

Next, we analysed the interaction between epiblast and hypoblast using the scRNA-seq data. In mice, GATA factors induce laminins in hypoblasts^25^, and basal lamina formation separates hypoblasts from epiblasts^45^. Our scRNA-seq data show that *LAMA1*, *LAMB1* and *LAMC1* were strongly expressed in nHyCs(G6-OE) (Extended Data Fig. 9a), and laminins formed at the boundary between nHyCs(G6-OE) and nEpiCs in the bilaminoids (Fig. 5g), therefore reflecting a basement membrane between the hypoblast and epiblast cells. Laminin is known to interact through integrin heterodimers on cell surface receptors^46^. We found that the integrin α_6_β_1_, which is required for the formation of rosette structure in mice^47^, is expressed in nEpiCs (Extended Data Fig. 9b), suggesting that, like in mice, laminin in nHyCs may act through integrins for rosette formation in humans. We therefore generated *LAMB1*-KO hPSC lines (Naive *LAMB1*-KO) (Extended Data Fig. 9c). Naive *LAMB1*-KO(G6-OE) cells differentiated into the hypoblast lineage (Extended Data Fig. 9d,e) but did not surround nEpiCs as a single cell layer nor did they support pro-amniotic cavity formation (Fig. 5h). We concluded that, like in mice, laminins secreted by the human hypoblast support epiblast differentiation and morphogenesis. We also noticed that nHyCs expressed *BMP* genes, *NODAL* and *WNT11* (Extended Data Fig. 9f) and nEpiCs expressed receptors related to *BMP*, *FGF* and *WNT* (Extended Data Fig. 9g). To examine how BMP, NODAL and WNT signalling affects mesoderm induction, we added activators and inhibitors from day 4 and found that BMP, WNT or activin inhibition reduces the appearance of gastrulation-related genes in nEpiCs on day 6 (Extended Data Fig. 9h).

## Lineage specification in bilaminoids

**Fig. 6:**
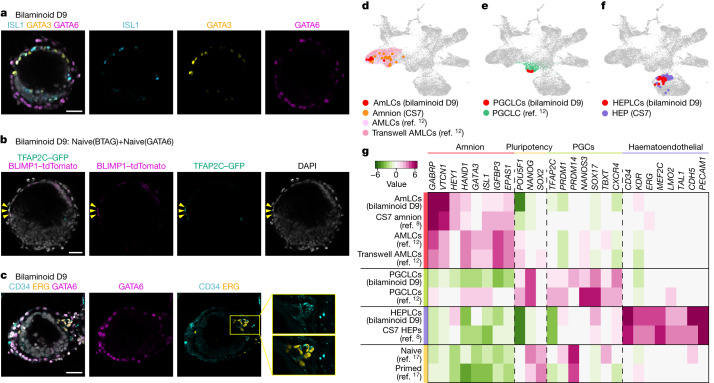
Bilaminoids recapitulate human pregastrulation. **a**, Amnion marker expression in bilaminoids on day 9. Blue, ISL1; yellow, GATA3; purple, GATA6; white, DAPI. *n* = 3 biologically independent experiments. **b**, PGC marker expression in bilaminoids on day 9. Bilaminoids were generated by *BLIMP1-tdTomato* and *TFAP2C-GFP* double-knockin Naive(BTAG) and Naive(G6-OE) cells. Green, TFAP2C–GFP; purple, BLIMP1–tdTomato; white, DAPI. The yellow arrowheads indicate BTAG double-positive cells. *n* = 4 biologically independent experiments. **c**, HEP marker expression in bilaminoids on day 9. Blue, CD34; yellow, ERG; purple, GATA6; white, DAPI. *n* = 3 biologically independent experiments. **d**–**f**, Amnion and amnion-like cells (AmLCs from bilaminoids and AMLCs from the model in Zheng et al.^12^) (**d**), PGC-like cells (PGCLCs) (**e**) and HEP and HEP-like cells (**f**) on UMAP for integrated datasets of bilaminoids on day 9, and published data as in Fig. 5a. **g**, Relative expression values of each tissue-specific marker gene in each cell type. Scale bars, 50 µm (**a**–**c**).

**Extended Data Fig. 10:**
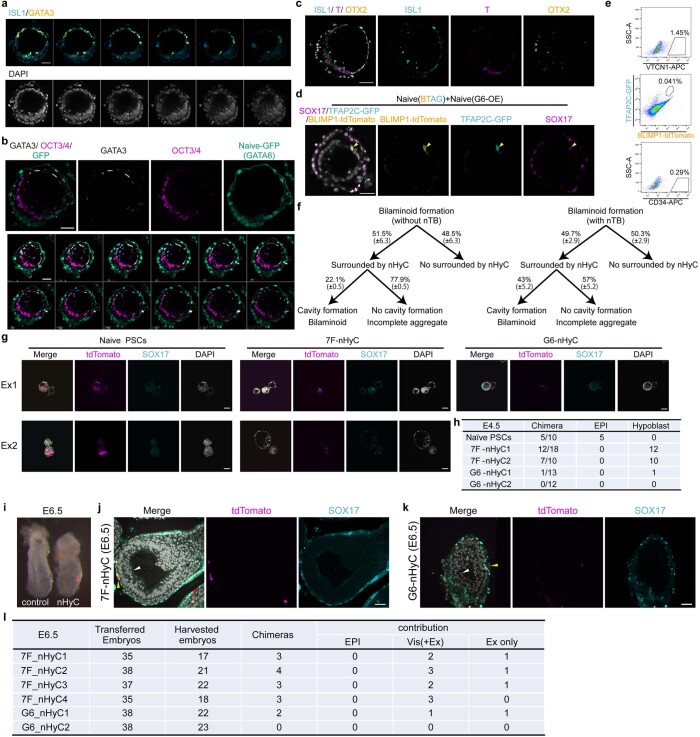
Bilaminoids on D9 and interspecies chimaera assays. a. Z-series images of bilaminoid on D9. A series of confocal Z-sections of a bilaminoid of Naïve (WT) and Naïve (G6-OE) on D9 stained for ISL1 (Blue), GATA3 (Yellow), and DAPI (White). N = 3. b. Z-series images of bilaminoid on D9. White, GATA3; Purple, OCT3/4; Green, Naïve-GFP(G6-OE); Blue, DAPI. N = 3. c. Anterior-posterior axis of bilaminoids on D9. Blue, ISL1; Purple, T; Yellow, OTX2; White, DAPI. N = 3. d. PGC marker gene expression in bilaminoids on D9. Bilaminoids were generated by BLIMP1-tdTomato and TFAP2C-GFP double knock-in Naïve (BTAG) and Naïve (G6-OE). BTAG- and SOX17-triple-positive cells are indicated by yellow arrowheads. Purple, SOX17; Blue, TFAP2C-GFP; Yellow, BLIMP1-tdTomato; White, DAPI. N = 3. e. VTCN1^+^ cells, BLIMP1-tdTomato^+^TFAPA2C-GFP^+^ cells, and CD34^+^ cells in D9 bilaminoids. VTCN1^+^ cells, BLIMP1-tdTomato^+^TFAPA2C-GFP^+^ cells, or CD34^+^ cells in D9 bilaminoids were sorted as single cells by flow cytometry and used for Smart-seq libraries. N = 3. f. The efficiency and workflow to obtain bilaminoids. Bilaminoids were analysed on D6. IL6 improved cavity formation similar to the co-cultures of nTB. g. Embryonic day (E) 4.5 hatched mouse-human chimaera blastocysts. Naïve hPSCs or 7F- or G6-nHyC were injected into mouse morula embryos. After injection, the embryos were cultured *in vitro* for two more days. Human cells are marked by tdTomato (Purple), and hypoblasts are marked by SOX17 (Blue). N = 2. h. The efficiency of mouse-human chimaera blastocysts at E4.5. nHyC never contributed to Epi. i. Interspecies chimaera embryos developed in utero at E6.5. 7F-nHyC (tdTomato)-injected embryos were transplanted into pseudopregnant mice on E3.5 and collected at E6.5. Control indicates non-chimaeric littermate. N = 4(7F), N = 2(G6). j. Confocal images of mouse-human chimaera embryo developed in utero at E6.5. 7F-nHyC-injected embryos were collected at E6.5 and stained for SOX17 (Green) and DAPI (White). nHyC-derived cells marked by tdTomato were detected in the visceral endoderm lesion (yellow arrowhead) and extraembryonic lesion (red arrows). White arrowhead, EPI. N = 4. k. Confocal images of mouse-human chimaera embryo developed in utero (E6.5). G6-nHyC-injected embryos were collected at E6.5 and stained by SOX17 (Green) and DAPI (White). nHyC-derived cells marked by tdTomato were detected in the visceral endoderm lesion (yellow arrowhead). White arrowhead, EPI. N = 2. l. A summary of mouse-human chimaera embryos at E6.5 in utero. 7F- and G6-nHyC-derived cells contributed to visceral endoderm in post-implantation embryos. (N=) shows biologically independent experiments. Scale bars, 50 µm.

Finally, we cultured the bilaminoids until day 9. The amniotic cavity of the bilaminoids enlarged, and nEpiCs partially differentiated into a flattened amniotic epithelium expressing the amnion markers ISL1 and GATA3 (Fig. 6a and Extended Data Fig. 10a–c). Notably, we also observed flattened epithelial cells expressing BLIMP1 and TFAP2C, markers for PGCs (Fig. 6b and Extended Data Fig. 10d), and CD34^+^ERG^+^ cells, markers for haematoendothelial progenitor (HEP) cells (Fig. 6c). We purified VTCN1^+^ cells, BLIMP1^+^TFAP2C^+^ (BTAG) cells and CD34^+^ cells as single cells using flow cytometry (Extended Data Fig. 10e). Integrated UMAP with human embryo cells showed that VTCN1^+^, CD34^+^ and BTAG cells clustered with embryonic amnion cells, PGCs and HEP cells, respectively (Fig. 6d–f). They also expressed embryonic amnion, PGC or HEP marker genes similar to published in vivo and in vitro controls (Fig. 6g). Although detailed characterization of these emerging cell types is necessary, this observation gives an early indication that bilaminoids support the progression of the epiblast from a blastocyst-like (naive state) to a post-implantation-like stage that is permissive for lineage specification (Fig. 6).

## Discussion

Here we highlight the crucial mechanistic roles of the two extraembryonic tissues—hypoblast and TB—to guide the progression and patterning of naive hPSCs into the post-implantation epiblast stage, thereby enabling them to generate subsequent lineages (for example, PGC-like and HEP cells) in a manner mimicking human embryogenesis.

Although naive hPSCs were reported to differentiate into the hypoblast lineage^23^, reanalysing the RNA-seq data revealed that they lack several pre-implantation hypoblast markers, suggesting that they resemble extraembryonic endoderm or mesoderm cells at the post-implantation stage. Thus, our study demonstrates robust and reproducible induction of pre-implantation hypoblast-like cells. In particular, FGF and BMP plus inhibition of WNT and activin A signalling pathways were critical for inducing naive hPSCs to hypoblasts specific to the pre-implantation-stage blastocyst. Our findings extend our understanding of the signalling pathways essential to specifying all three cell types of the blastocyst. Namely, naive epiblast can be maintained with FGF and aPKC inhibition, the trophectoderm with FGF and TGFβ inhibition, and the hypoblast with FGF and BMP4 activation plus TGFβ inhibition (Fig. 2i). These data also reveal that the signalling pathways that are required to induce the hypoblast of blastocysts in humans differ significantly from those in mice using either ACL^34^ or activin A + retinoic acid^30^, akin to the differences in trophectoderm induction.

However, hypoblast induction with the transcription factors GATA6 and GATA4 induces naive hPSCs to hypoblast, similar to in mice. Although transgene copy numbers and insertion sites may be variable because we used the PB system, we reproducibly obtained more than 80% PDGFRA^+^ cells from five independently established DOX-inducible GATA6 H9 hPSCs (Extended Data Fig. 2a). At the same time, our data show that the levels and duration of *GATA6* overexpression are critical.

To recapitulate a more in vivo like scenario and determine the in vivo function and contribution of G6-nHyCs and 7F-nHyCs, we performed mouse–human interspecies chimera assays. Whereas naive hPSCs integrated into the ICM, injected 7F- and G6-nHyCs contacted the ICM and expressed SOX17, similar to the late morulae–early blastocysts of mouse embryos, and never contributed to the epiblast lesion (Extended Data Fig. 10g–h). Furthermore, 7F- and G6-nHyCs contributed to the visceral endoderm and extraembryonic lesions in embryonic day 6.5 embryos, suggesting that both chemically and genetically induced nHyCs are functionally competent to form mouse–human chimera (Extended Data Fig. 10i–l). Notably, 7F-nHyCs contributed to the mouse visceral endoderm more efficiently than G6-nHyCs (Extended Data Fig. 10l). Although we titrated the DOX concentration, high levels of *GATA6* mRNA may have resulted in off-target effects and caused some functional disadvantages. As 7F induction is a non-genetic chemical induction method, 7F may enable naive hPSCs to differentiate into hypoblast under more physiologically relevant conditions compared with *GATA6* overexpression.

The hypoblast-like cells that we generated efficiently and reproducibly assemble into bilaminoids, proceeding to mimic human peri-implantation development, including the formation of the pro-amniotic-like cavity and anterior-posterior patterning of the epiblast. We showed by genetic modulation that this patterning is caused by a DKK1/OTX2 hypoblast-like domain. Although naive hPSCs can differentiate into trophectoderm and TB, we did not detect TB-like cells in bilaminoids until day 6, except in incomplete aggregates without an amniotic cavity (Extended Data Fig. 6p), even though TBs may emerge in later stages. Moreover, as there has been no report about the early stages of in vivo human amnion just after implantation (Carnegie stage 5), we could only estimate the gene expression profiles of the emergent amnion from Carnegie stage 7^8^, in vitro cultures of human embryos^5^ or primed hPSC-derived amnion-like cells^12^.

Notably, the separated co-cultures of additional trophectoderm-like cells enhanced the formation of the pro-amniotic-like cavity and early post-implantation epiblast growth (Extended Data Fig. 10f). This inducive effect by the trophectoderm is regulated in part by secreted molecules IL-6 and PDGF, as shown using both genetic- and chemical-based approaches. A recent report suggested that the in vitro early amnion expresses the AQP3 channel that may initiate amniotic cavity formation^48^. Furthermore, *AQP3* is one of the STAT3-target genes predicted by transcription factor binding motif analysis^49^. Furthermore, assay for transposase-accessible chromatin with sequencing (ATAC–seq) data suggest that this predicted STAT3-binding site in *AQP3* is open in both naive and primed hPSCs (Extended Data Fig. 7s). As our quantitative PCR (qPCR) data showed that *AQP3* was upregulated in epiblast-like cells by co-culture with nTB (Extended Data Fig. 7t), further studies may confirm an IL-6 dependency.

Recently, during revisions of this Article, stem-cell-based post-implantation models using in vitro epiblast- and hypoblast-like cells were reported^50–53^ (Supplementary Table 7). While the developmental window of our model extends from blastocyst to peri-gastrulation by starting with naive hPSCs that reflect day 5 pre-implantation epiblast and hypoblast, other models start from the post-implantation stage. Thus, our model covers a wider developmental time window from pre-implantation and precisely matches the natural developmental sequence and timing. Furthermore, considering that our bilaminoid model does not necessarily require genetic manipulation, it offers a flexible, alternative way for generating peri-implantation embryo models in vitro, with an efficiency that is comparable to the other models using RSeT and extended pluripotent stem cells (EPSCs)^51,52^ (Extended Data Figs. 6o and 7u,w and Supplementary Table 7). Importantly, functional assays with genetic modifications are almost impossible in human embryos but, using bilaminoids, we performed several lineage-specific gene modifications and identified interactions between these lineages. Finally, a limitation of our bilaminoids is that the amnion is covered by hypoblast when it should be in direct contact with the TB. Nevertheless, our study, together with the other human stem cell-based embryo models, will drive scientific discoveries in biomedical science.

## Methods

### Data reporting

The experiments were not randomized. The investigators were not blinded to the group allocation of experimental samples or the outcome assessment. No statistical methods were used to predetermine sample sizes.

### Ethics statement

Our embryo model lacks TBs and does not intend to recapitulate the full conceptus. Thus, our models are considered to be non-integrated embryo models and are not considered to be human embryos according to the ISSCR. Our work fully complies with current ISSCR 2016 and 2021 guidelines and follows the Guidelines on the Utilization of Human Embryonic Stem Cells in Japan. The CiRA Ethics Committee, an internal committee at CiRA, approved our research plan for human ES cell research (CiRA08-08), human iPSC research (CiRA18-21) and recombinant DNA experiments (190438). The WiCell lines H1 and H9 were used under agreements 10-WO-0098 and 10-WO-0099 for a research program entitled “Understanding mechanisms of pluripotency”. Bilaminoid models were generated using H9 ES cells, 551B1 iPSCs and 1390G3 iPSCs. These cell lines were consented for use in this study. Human-to-mouse interspecies chimera research was approved by the Research Ethics Committee of the University of Tokyo, and was conducted after receiving approval from the Ministry of Education, Culture, Sports, Science and Technology (MEXT) Japan after confirmation of compliance by the Specified Embryo Expert Committee. This approval includes the establishment of human iPSCs from peripheral blood samples. Signed informed consent was obtained from the volunteers before human peripheral blood samples were collected to establish iPSCs. The approved iPSC line, PB004, was used for interspecies chimera assays.

### Cell culture

Cells were cultured under 5% O_2_ and 5% CO_2_. Human ES cell lines H1 and H9 (WiCell Research Institute) and human iPSCs (AdiPSCs^18^, 585B1^54^ and 1390G3^55^) were cultured on mouse embryonic fibroblasts (MEFs) (1 × 10^6^ cells per six-well plate).

Primed hPSCs were maintained in DMEM/F12 (08460-95, Nacalai Tesque) containing 20% Knockout Serum Replacement (10828028, Thermo Fisher Scientific), 1% non-essential amino acids (11140-050, Thermo Fisher Scientific), 4 ng ml^−1^ recombinant human basic fibroblast growth factor 2 (bFGF; NIB 47079000, Oriental Yeast) and 0.1 mM 2-mercaptoethanol (M3148, Sigma-Aldrich). Cultures were passaged every 5–7 days as small clumps using dissociation buffer containing 0.025% trypsin (15090-046, Thermo Fisher Scientific), 1 mg ml^−1^ collagenase IV (17104-019, Thermo Fisher Scientific), 20% Knockout Serum Replacement and 1 µM CaCl_2_.

Naive hPSCs were maintained in t2iLGo medium, consisting of a chemically defined medium, N2B27 (NDiff 227, Y40002, Takara Bio) supplemented with 1 µM PD0325901 (PD03; 4192, Tocris), 1 µM CHIR99021 (CH; SML1046, Sigma-Aldrich), 10 ng ml^−1^ recombinant human LIF (hLIF; 300-05, Peprotech) and 3 µM Go6983 (Go; 2285, Tocris) as previously described^18^. The components of the N2B27 medium were DMEM/F12, Neurobasal medium, N2 and B27^56^. Naive hPSCs were passaged every 3–5 days using Accutase (A6964, Sigma-Aldrich).

Resetting primed hPSCs to naive hPSCs by NANOG and KLF2 overexpression was performed as previously described^18^. In brief, PB vectors (2 µg) carrying DOX-inducible *KLF2* or *NANOG* and a PB-M2rtTA expression vector (2 µg) were co-transfected with pBase helper plasmid (4 µg) using the Neon Transfection System (Program 14, Invitrogen). The medium was switched to t2iL plus DOX (1 µM) for resetting. Cells were split every 5–7 days after dissociation with Accutase. After 2 weeks, DOX was withdrawn, and the PKC inhibitor Go6983 (3 µM) was added (t2iLGo). Cells were maintained on MEF feeders throughout.

Chemical conversion to naive hPSCs was performed as previously described^20^. Primed hPSCs (1 × 10^4^ cells per cm^2^) were seeded onto MEF feeder cells under primed hPSC medium with 10 µM Y-27632. The medium was switched the next day to cRM-1 (N2B27, 1 µM PD03, 10 ng ml^−1^ hLIF, and 1 mM valproic acid sodium salt (P4543, Sigma-Aldrich)). On day 3, the medium was replaced with cRM-2 (N2B27, 1 µM PD03, 10 ng ml^−1^ hLIF, 2 µM Go and 2 µM XAV939; X3004, Sigma-Aldrich). Dome-shaped naive colonies were observed around 2 weeks after seeding. Reset cells were passaged and maintained on MEF feeders under t2iLGo. Chemical conversion to naive hPSCs using 5iLA was also performed as described previously^19^. Here, 2 × 10^5^ cells per cm^2^ were seeded on MEF feeder cells under primed hPSC medium with 10 µM Y-27632. The medium was switched the next day to 5iLA medium (N2B27 plus 1 µM PD03, 1 µM CH, 1 µM WH-4-023 (H620061), 0.5 µM SB590885 (2650, R&D Systems), 10 µM Y-27632, 10 ng ml^−1^ hLIF and 20 ng ml^−1^ activin A (338-AC-010, R&D Systems). After conversion to naive hPSCs, the cells were maintained under t2iLGo on MEF feeder cells.

Mouse ES cells were cultured on a gelatine-coated dish in 2iL (N2B27, 1 µM PD03, 3 µM CH and 10 ng ml^−1^ hLIF). Cells were passaged every 2–3 days using Accutase.

Naive hPSCs form tightly packed small colonies and expressed GFP if carrying the EOS-GFP reporter, which consists of an *OCT3/4* distal enhancer and an early transposon promoter^18–20,57^ (Extended Data Fig. 1a). Naive hPSCs expressed the naive-specific genes *KLF17* and *TFCP2L1* (Extended Data Fig. 1b,c) but primed and expanded PSCs did not^58^. All cell lines were routinely checked for mycoplasma contamination (Lonza–MycoAlert), and all samples analysed in this study were not contaminated.

### *GATA6* overexpression

*GATA6*, *GATA4* and *SOX17* were cloned into a DOX-inducible PB vector coupled to a rtTA expression construct (KW110)^59^. PB-GATA6 vector (2 µg), PB-GATA4 vector (2 µg) or PB-SOX17 vector (2 µg), and pBase helper plasmid (2 µg) were transfected into naive or primed hPSCs using the Neon Transfection System (Program 20 for naive hPSCs; Program 14 for primed hPSCs). Then, 2 days later, G418 was added (200 µg ml^−1^) for about 2 weeks. Naive or primed hPSCs with inducible *GATA6*, *GATA4* or *SOX17* were maintained in naive or primed medium. For transgene induction, MEF feeder cells were removed by incubation on a gelatine-coated dish after dissociation to single cells. Then, 1 × 10^5^ cells per cm^2^ were seeded into a dish coated with fibronectin (FC010, Millipore) or iMatrix-511 silk (Laminin511-E8) (892021, Matrixome). The serum medium consisted of GMEM (G5154, Sigma-Aldrich), FBS (10437028, Thermo Fisher Scientific), 2 mM l-glutamine (25030081 Thermo Fisher Scientific), 1 mM sodium pyruvate (11360-070, Thermo Fisher Scientific), NEAA and 0.1 mM 2-ME. Hypoblast induction by serum medium is shown in Fig. 1b and Extended Data Fig. 1d–h. Except for these experiments, all other analyses were performed in serum-free conditions. As a serum-free basal medium, we used the N2B27 medium (NDiff 227; Y40002, Takara Bio). The components of the N2B27 medium were DMEM/F12, Neurobasal, N2 and B27^56^. BSA is included in N2 and B27. For nHyC induction, 25 ng ml^−1^ recombinant human FGF4 (FGF4; 100-31) and 1 µg ml^−1^ heparin sodium (081-00131, Wako) were added to the basal medium. The medium was changed every day.

### Hypoblast specification using chemical components

In brief, 5 × 10^4^ per cm^2^ naive hPSCs were seeded onto laminin511-E8 in the N2B27 medium. Six factors, 25 ng ml^−1^ FGF4 (+1 µg ml^−1^ heparin sodium), 10 ng ml^−1^ recombinant human BMP4 (BMP4; 314-BP, R&D), 10 ng ml^−1^ recombinant human PDGF-AA (Peprotech, 100-13A), 1 µM XAV939, 3 µM A83-01 (2939, Tocris) and 0.1 µM retinoic acid (R2625, Sigma-Aldrich), were added on day 0. On day 2, the medium was switched to seven factors (six factors and 10 ng ml^−1^ recombinant human IL-6) (IL-6; 47066000, Oriental Yeast). In some experiments, 500 ng ml^−1^ recombinant human BMP2 (BMP2; 47304000, Oriental Yeast) or 50 ng ml^−1^ recombinant human BMP6 (BMP6; 120-06, Peprotech) was used instead of BMP4. N2B27 medium without vitamin A was made in house.

### Hypoblast induction from mouse ES cells

Two previously reported protocols were used for hypoblast induction from mouse ES cells^30,34^. Mouse ES cells were maintained under 2iL conditions. In the first protocol, 5 × 10^4^per cm^2^ mouse ES cells were seeded onto gelatine under RPMI 1640 (12633012, Thermo Fisher Scientific) with 2 mM l-glutamine, B27 minus insulin (A1895601, Gibco), 20 ng ml^−1^ activin A, 3 µM CHIR and 10 ng ml^−1^ hLIF^34^. In the second protocol, 5 × 10^4^ per cm^2^ mouse ES cells were seeded onto gelatine under 10 nM retinoic acid and 20 ng ml^−1^ activin A^30^. The medium was changed every day in both conditions.

### Marmoset embryo cultures

All animal experiments were approved by the Animal Experiment Committee at CiRA and Kyoto University (Approval number 16-75-6) and the Institutional Animal Care and Use Committee of the Central Institute for Experimental Animals (CIEA: 17029A and 18031A). Naturally fertilized embryos were collected from the uterus by non-invasive flushing^60^. Embryos (morulae or blastocyst) were cultured under Sequential Blast (Origio, 83050010). When embryos reached the blastocyst stage, the zona pellucidae were removed using acidic Tyrode’s solution (Sigma-Aldrich), and the embryos were processed for immunosurgery using a custom rabbit polyclonal anti-marmoset antibody. ICM were seeded on laminin511-E8 under N2B27 plus 7F, 4F (FGF4, BMP4, A83, XAV) or control (PD03, LDN, A83, XAV) for 3 days, fixed and analysed using anti-SOX17 antibodies.

### Generation of bilaminoids

Ten naive hPSCs (Naive(WT)) and 40 naive hPSCs or GFP-expressing naive hPSCs expressing *GATA6* under DOX treatment (Naive(G6-OE) or naive-GFP(G6-OE)) were seeded in each well of a microwell array^36^ or Elplasia plate (4441, Corning) under t2iLGo plus 10 μM Y27632 without Matrigel or Geltrex. After 24–36 h of aggregation (day 0), the medium was switched to N2B27 with 0.1 μM DOX. On day 2, DOX was withdrawn. Bilaminoids were cultured under N2B27 until day 10. To identify the signalling pathways involved, 10 ng ml^−1^ BMP4, 300 nM LDN193189 (LDN, SML0559, Sigma-Aldrich), 3 µM A83-01, 10 ng ml^−1^ activin, 1 µM XAV and 1 µM CHIR were added from day 4 to day 6. In the experiments noted in the text, 10 ng ml^−1^ IL-6, 1 µM JAK inhibitor 1 (JAKi, 420099, Sigma-Aldrich) or 10 ng ml^−1^ PDGF-AA was added from day 0 to day 4. The medium was changed every day. To collect PGCLCs, bilaminoids were cultured under N2B27 + 200 ng ml^−1^ BMP4 from day 5 to day 9.

### Co-culture with bilaminoid and nTB

Bilaminoid and nTB were co-cultured using a cell culture insert (Transwell). nTB was induced from naive hPSCs on the Transwell. Bilaminoids were generated by culturing a mixture of 10 naive hPSCs (Naive(WT)) and 40 naive hPSCs or GFP-expressing naive hPSCs expressing *GATA6* under DOX treatment (Naive(G6-OE) or Naive-GFP(G6-OE)) in each well of an Elplasia plate under t2iLGo plus 10 µM Y27632. After 24–36 h of aggregation (day 0), nTB on the Transwell was placed on the Elplasia plate under N2B27 with 0.1 µM DOX. On day 2, the DOX was withdrawn. Co-cultures continued until day 4.

### Aggregates generated by hPSCs and sorted cells

A mixture of 100 naive or primed hPSCs and 100 sorted cells expressing GFP (naive 7F-, 4F-, G6-PDFRA^+^ cells, primed G6-PDGFRA^+^ cells, PDGFRA^+^ RACL cells, and CXCR4^+^CDH1^+^ definitive endoderm cells) were seeded in each well of an Elplasia plate under N2B27 plus 10 μM Y27632. The medium was changed every other day. Aggregates were evaluated on day 4.

### Generation of *LAMB1*-KO lines

To KO the *LAMB1* gene, two gRNAs targeting exon 3 (gRNA 1)^61^ and exon 6 (gRNA 2) of human *LAMB1* were designed and inserted into pSpCas9(BB)-2A-mCherry (Extended Data Fig. 9c): gRNA 1, 5′-GTCCTGGGCTCAAGTCGAT-3′; and gRNA 2, 5′-ATCTTGCTAGCAGGCTGAAA-3′. pSpCas9/gRNA plasmid (5 μg) was electroporated into primed H9 human ES cells (Neon Program 14). Then, 2 days later, mCherry^+^ cells were sorted by flow cytometry and seeded at a low density. About 10 colonies were picked 7–8 days after seeding, and genomic DNA was extracted. DNA was amplified and sequenced using the following primers: gRNA 1, Fw 5′-CCCCCGCTTGTTCGTTTTTTTCGG-3′, Rv 5′-TCACCTGCAAGTGGCTGACGATACAG-3′; and gRNA 2, Fw 5′-TCCGTGTCCTTCTCCTTTCG-3′, Rv 5′-CAGGAAATGTGTGGCGGATG-3′. The generated *LAMB1*-KO primed hPSCs were reset to naive hPSCs.

### Generation of *CER1*-knockin lines

*CER1-H2B-GFP* reporter cells were generated from primed H9 human ES cells by replacing the endogenous stop codon of the *CER1* gene with a T2A-H2B-GFP-LoxP-SV40-NeoR-LoxP cassette using CRISPR–Cas9 homology-directed repair (Extended Data Fig. 8i). H2B–GFP accumulates in the nucleus. gRNA targeting the stop codon of human *CER1* was designed and inserted into pX330-U6-Chimaeric_BB-CBh-hSpCas9: gRNA, 5′-TCCCAGGATTCCTTTATCCCAGG-3′. For the donor vector, approximately 1,000 bp upstream and downstream of the CRISP–Cas9 cleavage site was prepared by long PCR, fused with a T2A-H2B-GFP-LoxP-SV40-NeoR-LoxP cassette and cloned into a TOPO vector. pSpCas9/gRNA and the donor vector (1 μg each) were electroporated into primed H9 human ES cells (Neon Program 14). Then, 2 days later, G418 was added (200 µg ml^−1^) for about 2 weeks. The cells were collected and seeded on MEFs at a low density. Colonies were picked 7–8 days after seeding, and genomic DNA was extracted. DNA was amplified by PCR and sequenced. The *SV40-NeoR* gene was deleted from the *CER1-H2B-GFP* line by the transient introduction of a *cre*-expressing vector. The generated *CER1-H2B-GFP-*primed hPSCs were reset to naive hPSCs.

### The measure of the anterior–posterior axis of bilaminoids

Angles between T^+^ nuclei and CER1–H2B–GFP, OTX2, LEFTY or DKK1 nuclei on sections of bilaminoids were analysed. The centre of the T^+^ nuclei was defined as 0°. Angles were averaged for each aggregate.

### Generation of *IL6*-KO lines

To KO the *IL6* gene, two sgRNAs that targeting exon 2 (sgRNA 1) and exon 3 (sgRNA 2) of human *IL6* were designed and inserted into pSpCas9(BB)-2A-mCherry (Extended Data Fig. 7j): sgRNA 1, 5′-GAAGTCTTGCTTAACTGTTTG-3′; and gRNA 2, 5′-TAGACCTAAGTTACTCCATG-3′. pSpCas9/sgRNA plasmid (5 μg) was electroporated into primed H9 human ES cells (Neon Program 14). Then, 2 days later, mCherry^+^ cells were sorted by flow cytometry and seeded at a low density. Colonies were picked 7–8 days after seeding, and genomic DNA was extracted. DNA was amplified and sequenced using the following primers: sgRNA 1, Fw 5′-AGCCCACCGGGAACGAAAGAGAAGCT-3′, Rv 5′-GGCAGAACCAGAATTCGAGTGTGGGCTC-3′; and sgRNA 2, Fw 5′-GAACACAGGAGGGGAGATTGGGAGCCCA-3′, Rv 5′-GGGGATCCTTCTCTGATTGTCCCCCTTG-3′. The generated *IL6-*KO primed hPSCs were reset to naive hPSCs.

### Measurement of IL-6

Naive hPSCs were plated (1.5 × 10^5^ cells per cm^2^) on iMatrix-coated Transwell plates and differentiated into nTB as described above (day 0). On day 3, the nTB induction medium was replaced with NDiff 227. As controls, hPSCs were plated (1.5 × 10^5^ cells per cm^2^) on iMatrix-coated Transwell plates under each medium (naive hPSCs, t2iLGo; primed hPSCs, AK02N). On day 3, the hPSC medium was replaced with NDiff 227. The cell culture supernatants were collected on day 5 and centrifuged to remove debris. The levels of IL-6 were quantified using an IL-6 ELISA kit (Abcam, ab178013) according to the manufacturer’s protocol. The absorbance at 450 nm was measured using a plate reader (TECAN, Infinite 200 PRO). Each sample was analysed in duplicates.

### Generation of naive hPSCs overexpressing *OTX2*, *DKK1* and *GP130/GCSFR*

For *OTX2* overexpression, *OTX2* fused to *ERT2* was inserted into the PB vector (PB-OTX2-ERT2). The PB-OTX2-ERT2 vector and pBase helper plasmid were transfected into naive hPSCs expressing GATA6 under DOX treatment (Naive(G6-OE)). To generate bilaminoids, *OTX2-ERT2* was activated by treatment with 100 nM 4-hydroxytamoxifen (tamoxifen) from day 4 to day 6.

For *DKK1* overexpression, *DKK1* fused to destabilizing domain (*DD*) was cloned into the PB vector (PB-DD-DKK1). The PB-DD-DKK1 vector and pBase helper plasmid were transfected into naive hPSCs expressing GATA6 under DOX treatment (Naive(G6-OE)). To generate bilaminoids, *DD-DKK1* was activated by treatment with 500 nM Shield1 (Takara, 632189) from day 4 to day 6.

To activate JAK/STAT3 signalling, *GP130*/*GCSFR* chimeric receptor (Y118F) cDNA was inserted into the PB vector (PB-Y118F). The PB-Y118F vector and pBase helper plasmid were transfected into naive hPSCs (Naive(WT) or Naive(G6-OE)). To generate bilaminoids, STAT3 signalling was activated the treatment with G-CSF from day 0 to day 4.

### RACL induction from naive hPSCs

Naive hPSCs (H9) were differentiated under RACL conditions as described previously^23,62^. The cells were plated (5 × 10^4^ per cm^2^) onto MEF feeder cells and cultured under RACL medium, composed of RPMI 1640 medium with GlutaMAX (61870036, Thermo Fisher Scientific), B27 minus insulin (A1895601, Gibco), 100 ng ml^−1^ activin A, 3 μM CHIR, and 10 ng ml^−1^ LIF, for 7 days. The medium was changed every other day. On day 7, the cells were dissociated by Accutase, and PDGFRA^+^ cells were sorted. Anti-feeder antibody was used to remove the MEF feeder cells.

### Definitive endoderm induction

Primed hPSCs were differentiated into definitive endoderm as described previously^63^. Primed hPSCs were seeded on an uncoated bacterial dish to form EBs under StemFit AK02N (AK02N, Ajinomoto) plus 10 μM Y27632. After 2 days, the EBs were washed and cultured under N2B27 with 200 ng ml^−1^ activin A and 3 µM CHIR. The next day, the medium was replaced with N2B27 and 200 ng ml^−1^ activin A and cultured for 2 more days. The EBs were dissociated using Accutase, and CXCR4^+^CDH1^+^ definitive endoderm cells were sorted and used for the experiments.

### TB induction from naive hPSCs

Naive hPSC-derived TB-like cells (nTBs) were induced as described previously^21,64^. H9 naive hPSCs (5 × 10^4^ cells per cm^2^) were plated onto laminin511-E8 (0.15 µg cm^−2^ iMatrix511 silk) under NDiff 227, 2 µM A83-01, 2 µM PD03, 10 ng ml^−1^ BMP4 and 10 µM Y27632. The next day, the medium was changed to NDiff 227, 2 µM A83-01, 2 µM PD03, and 1 µg ml^−1^ JAK inhibitor I (JAKi, 420099, Sigma-Aldrich). On day 3, the cells were dissociated by Accutase, and HAVCR1^+^ENPEP^+^ (refs. ^21,22,65,66^) nTBs were sorted and recultured for further experiments.

### Transwell assay

The Transwell assay was performed as previously described^12^ on Transwell 12-well plates with porous polyester membrane inserts (0.4 µm pore size; Corning). The membrane inserts were coated with 1% Geltrex diluted in DMEM/F12 for 1 h. For amnion-like cell induction, primed hPSCs were seeded on membrane inserts at a density of 3 × 10^4^ cells per cm^2^ under mTeSR plus 10 μM Y27632. Then, 18 h after cell seeding, the medium was switched to E6 supplemented with bFGF (20 ng ml^−1^) and BMP4 (50 ng ml^−1^) and cultured for 48 h. For G6-nHyCs and 7F-nHyCs, day 3 PDGFRA^+^ cells were sorted and recultured on membrane inserts at a density of 9 × 10^4^ cells per cm^2^ overnight. Primed hPSCs were collected as small clumps and seeded onto the membrane inserts under E6 medium supplemented with bFGF (20 ng ml^−1^). The cells were cultured for another 48 or 96 h before analysis. The medium was changed every other day.

### Flow cytometry and cell sorting

Cells were dissociated into single cells by Accutase or trypsin, washed and blocked in HBSS (14185052, Thermo Fisher Scientific) with 1% BSA (A2153, Sigma-Aldrich) on ice for 30 min. Staining was performed on ice with the following: biotinylated PDGFRA antibodies (BAF322, R&D), CEACAM1 + CEACAM5 antibodies (Ab91213, Abcam) and directly conjugated antibodies in HBSS with 1% BSA for 30 min. After washing, Streptavidin-APC (405207, BioLegend) was used as the secondary antibody for PDGFRA–biotin. Alexa Fluor 488 was used for the CEACAM1 antibody. Flow cytometry and cell sorting were performed on the BD LSR Fortessa (BD) or FACS Aria II (BD) system. Data were analysed using FlowJo v.10.7.2. A list of the antibodies used is provided in Supplementary Table 7.

### qPCR with reverse transcription

Total RNA was extracted using the RNeasy Kit (74106, Qiagen). Total RNA (0.5 µg) was reverse-transcribed into cDNA with an oligo-dT primer using SuperScriptIV (18090050, Thermo Fisher Scientific). qPCR was performed using QuantStudio3 (Thermo Fisher Scientific) and QuantStudio12K (Thermo Fisher Scientific) with TaqMan Fast Universal Master Mix (4364103, Thermo Fisher Scientific) and TaqMan probe or PowerUP SYBR Green Master Mix (A25743, Thermo Fisher Scientific) according to the manufacturer’s instructions. The results were analysed using QuantStudio Design & Analysis v.1.4.1 (Thermo Fisher Scientific).

### Immunostaining

Cells were fixed in 4% paraformaldehyde (09154-85, Nacalai Tesque) for 10 min at room temperature. After fixation, the cells were washed with PBS, permeabilized in PBS plus 0.5% Triton X-100 for 1 h, and blocked in PBS plus 1% BSA and 0.05% Tween-20 (PBS-BT) for 2 h. Primary antibodies were diluted in PBS-BT and incubated at 4 °C overnight. After washing, secondary antibodies were diluted at 1:2,000 and incubated at room temperature for 2 h or at 4 °C overnight. Nuclei were stained with DAPI. Fluorescent images were obtained using the confocal laser scanning microscope TCS SP8 (Leica) or LSM710 (Zeiss). Cavity volume (Fig. 4e,h) was quantified from confocal *z*-stack images using Imaris software v.10.0.0 (Bitplane). PAR6 and F-actin images were used to quantify cavity volume with the Surfaces program.

### Western blot analysis

For western blot analysis, 1 × 10^6^ cells were lysed with RIPA buffer (08714-04, Nakalai Tesque). SDS sample buffer was added, and the mixture was incubated at 93 °C for 3 min. The extracted proteins were separated on Bollt 4–12%, Bis-Tris, 1.0 mm, Mini Protein Gel (NW04120BOX, Thermo Fisher Scientific) and blotted onto an Immobilon-P PVDF Membrane (IPVH00010, Merck) using a Mini PROTEAN Tetra Cell (Bio-Rad). The transferred membranes were incubated with the following primary antibodies: α-tubulin (ab7291, Abcam), pSMAD1/5/9 (9511, Cell Signaling Technology), pSMAD2 (3108, Cell Signaling Technology), pMAPK (4376, Cell Signaling Technology), pSTAT3 (9131, Cell Signaling Technology) and STAT3 (564533, BD Bioscience). The primary antibodies were detected with anti-rabbit IgG, HRP-linked antibodies (7074, Cell Signaling Technology) and anti-mouse IgG, HRP-linked antibodies (7076, Cell Signaling Technology), followed by detection using ECL Prime Western Blotting Detection Reagent (RPN2236, Amersham). Chemiluminescence images were acquired using the ImageQuant LAS 4000 (GE Healthcare) and Ambersham ImageQuant 800 (Cytiva) systems. Uncropped western blot images are shown in Supplementary Figs. 2 and 3.

### RNA-seq analysis

For RNA-seq, samples were collected after removing MEFs by gelatine treatment. RNA was purified using the miRNeasy Mini Kit (217004, Qiagen), and 200 ng RNA and the TruSeq Stranded mRNA LT Sample Prep Kit (RS-122-2101, Illumina) were used for library construction. RNA-seq libraries were sequenced using the NextSeq 500 High Output v2 Kit (75 Cycles, FC-404-2005) (Illumina). The sequenced reads were trimmed to remove low-quality bases and adaptor sequences using cutadapt (v.1.15)^67^. The trimmed reads were mapped to the human reference genome (hg38) using TopHat2^68^ with GENCODE v.27^69^. Uniquely mapped reads (MAPQ ≥ 20) were used for further analyses. Each gene expression level was calculated as reads per kilobase per million mapped reads (FPKM) using cufflinks (v.2.2.1)^70^. Genes expressed at low levels (defined as genes with FPKM < 5: UHC, PCA, FPKM < 1: correlation coefficients) across all samples in each dataset were excluded from subsequent analyses. Expression values were normalized to the median or mean of all datasets or a specific condition. Heat-map preparation, correlation analyses, hierarchical clustering analyses and PCA were performed using R (v.3.3.2). The correlation analysis in Fig. 2d examined differentially expressed genes in the epiblast and hypoblast of human embryos^7^ (Supplementary Table 3). The ontogenic gene set between TB and amnion determined previously^66^ was used for correlation analysis in Extended Data Fig. 8e. We analysed the following previously published RNA-seq datasets available at the Gene Expression Omnibus (GEO): GSE138012 (ref. ^23^), GSE52658 (ref. ^71^) and GSE75748 (ref. ^35^).

### scRNA-seq analysis (Smart-seq)

Bilaminoids (D6) were manually picked. Before sampling bilaminoids, aggregates surrounded by nHyCs were identified by stereomicroscope and amniotic cavity formation was additionally confirmed by microscopy using the Celldicoverer 7 (Zeiss) system (Extended Data Fig. 8a). The choice of the right bilaminoids that contain an amniotic cavity is critical. Each bilaminoid was transferred in a drop of Accutase and incubated at 37 °C for 15–20 min, then dissociated into single cells by repeated pipetting using glass capillaries. Each single cell was transferred into individual PCR tubes and immediately frozen in Smart-seq HT lysis buffer.

To collect amnion-like cells (AmLCs), PGCLCs and HEP-like cells (HEPLCs), bilaminoids on day 9 were dissociated by Accutase. VTCN1^+^ cells (AMLCs), TFAP2C-GFP^+^BLIMP1-tdTomato^+^ cells (PGCLCs) or CD34^+^ (HEPLCs) were sorted as single cells and immediately frozen in Smart-seq HT lysis buffer.

The libraries for scRNA-seq were prepared using the SMART-seq HT kit (Z4436N, Takara) according to the manufacturer’s instructions. The libraries were then sequenced on the NovaSeq 6000 or NextSeq 500 (Illumina) system with paired-end sequencing.

Single-cell data analyses were performed according to the methods described previously^43^ and associated scripts (from https://github.com/zhaocheng3326/CheckBlastoids_scripts). In brief, human embryonic datasets, cells from the PASE model and blastoids datasets were downloaded as described previously^43^. The downloaded datasets and our Smart-seq HT data were preprocessed and quantified for gene expression as described in the above scripts based on each single-cell method. We used the Cell Ranger pipeline (v.3.1.0, 10x Genomics) for all human 10x Genomics single-cell datasets and STAR aligner (v.2.5.1b) and RSEM (v.1.3.1) tool for Smart-Seq datasets. To minimize bias on gene expression data, downloaded raw sequencing data were mapped to the same human reference genome (refdata-cellranger-GRCh38-3.0.0 downloaded from the 10x Genomics website) and quantified for gene expression in the same computational environment. According to the above CheckBlastoids scripts with the gene expression matrices, we performed quality control, normalization, cell annotation, integrated analyses, clustering and visualization using the R Seurat package (v.4.0.4).

### Data comparison with bulk RNA-seq and published scRNA-seq

To minimize data processing discrepancies (Fig. 2h), the raw fastq files of published SMARTer v2 scRNA-seq data^5^, published bulk RNAseq^21,23,35^ and our bulk RNA-seq data were mapped and quantified into count data using STAR (v.2.7.8a; --soloType SmartSeq) with the same reference genome used in the Cell Ranger pipeline described above. On the basis of the published scRNA-seq data^5^, cells with nfeature > 6,000, nCount between 50,000 and 1,800,000, and low mitochondrial gene expression (<15%) were used for further analysis. We reannotated 173 cells and identified them as pre-Epi, post-Epi, PSA-Epi, intermediate post-Epi, intermediate PSA-Epi, hypoblast, pre-trophectoderm and post-implantation CT (Supplementary Table 3). The count matrix of SMARTer v.2 scRNA-seq data and our bulk RNA-seq were imported into R (v.3.5.1) using DeSeq2 v.1.22.2, and the expression levels were calculated as transcripts per million (TPM). Low-expression genes (TPM < 5 in all samples) were excluded, and log-scaled TPM values were used to perform the PCA analysis using R (v.3.5.1).

### ATAC–seq analysis

ATAC–seq data of naive and primed hPSCs were obtained from the GEO (GSE101074)^72^. ATAC–seq signals of naive hPSCs and primed hPSCs at the *AQP3* locus were visualized by Integrative Genomics Viewer (IGV). The predicted STAT3-binding sites were previously reported^73^.

### Interspecies chimera formation

The human iPSC line PB004, following approval by the ethics committee at the University of Tokyo and by MEXT (Ministry of Education, Culture, Sports, Science, and Technology) Japan, was used for interspecies chimera experiments. Ten cells of naive hPSCs or nHyC induced by 7F or GATA6 and sorted by PDGFRA on day 3 were microinjected into mouse morula embryos. Then, 2 days after the injection, 7F-nHyCs and naive hPSCs were confirmed to have contributed to late morulae-early blastocysts of mouse embryos.

BDF1xB6 mouse embryos were collected in M2 medium (M7167, Sigma-Aldrich) at the eight-cell or morula stage, transferred into KSOM medium (MR-121, Sigma-Aldrich), and cultured for several hours. A piezo-driven micro-manipulator (Primetech) was used to drill into the zona pellucida under a microscope, and 10 naive hPSCs or nHyCs were introduced into the subzonal space of each embryo. After the injection, the embryos underwent follow-up culture in N2B27 medium until the blastomere stage. They were then transferred into the uteri of pseudopregnant recipient ICR mice for in vivo chimera assays. For in vitro chimera assays, chimeric embryos were cultured under N2B27 medium for 2 days.

### Statistical analysis and reproducibility

Statistical tests were performed using GraphPad Prism v.9.4.1 and v.10.0.3. Errors and error bars represent the s.e.m. from a minimum of three independent experiments. In all of the experiments, the number of biologically independent experiments is indicated in the caption. *n* values in the figure panels represent the number of aggregates analysed. All of the experiments were performed independently at least twice. Data were tested for normality using the Shapiro–Wilk test. Normally distributed data were analysed using parametric tests (unpaired *t*-test or analysis of variance), and non-normally distributed data were analysed using nonparametric tests (Mann–Whitney *U*-tests or Kruskal–Wallis test) as indicated in figure legends. Regarding the efficiency of generating aggregates, significant differences among conditions were evaluated using Fisher’s exact test based on the total number of aggregates analysed.

In Fig. 1b, representative images of ten biologically independent experiments are shown (*n* = 10). In Fig. 1c, typical results of three biologically independent experiments are shown (*n* = 3). In Fig. 2b, representative images of 44 biologically independent experiments are shown (*n* = 44). In Fig. 2c, representative images of two biologically independent experiments are shown (*n* = 2). In Fig. 2e, typical results of three biologically independent experiments are shown (*n* = 3). In Fig. 2f,g, representative images of two biologically independent experiments are shown (*n* = 2). In Fig. 3b, representative images of three biologically independent experiments are shown (*n* = 3). In Fig. 3c, bar charts show the mean value of two biologically independent experiments (*n* = 2). In Fig. 3d, representative images of two biologically independent experiments are shown (*n* = 2). All F-actin-accumulated aggregates co-expressed PODXL or aPKC. About 10% of aggregates contained a cavity. In Fig. 4b, cell number was measured from two biologically independent experiments (*n* = 2). A total of ten aggregates on each day was counted. In Fig. 4c, representative images of two biologically independent experiments are shown (*n* = 2). In Fig. 4d, the cavity formation rate was measured from two biological replicates (*n* = 2). In Fig. 4e, the cavity volume was measured from three biological replicates (*n* = 3). In Fig. 4f, representative images of four biologically independent experiments are shown (*n* = 4). In Fig. 4g, the cavity formation rate was measured from four biological replicates (*n* = 4). In Fig. 4h, the cavity volume was measured from four biological replicates (*n* = 4). In Fig. 4i, the cavity formation rate was measured from three biological replicates (*n* = 3). In Fig. 4j, representative images of five biologically independent experiments are shown (*n* = 5). In Fig. 5d, angles were measured from three biologically independent experiments (*n* = 3). A total of ten aggregates expressing *T* and *CER1-H2B-GFP* were analysed. Angles were averaged for each aggregate. In Fig. 5e, a total of 14 aggregates expressing *T* and *OTX2* were analysed (*n* = 3). Angles were averaged for each aggregate. In Fig. 5f, the proportion of *T*-expressing bilaminoids was measured from two biological replicates (*n* = 2). In Fig. 5g, 24 aggregates were analysed in naive(WT) cells only (*n* = 2). Most aggregates (32 out of 36) surrounded by nHyCs(G6-OE) formed basement membranes between nHyCs and nEpiCs (*n* = 2). In Fig. 5h, the cavity formation rate was measured from three biological replicates (*n* = 3). In Fig. 6a, representative images of three biologically independent experiments are shown (*n* = 3). Approximately 68% of day 9 bilaminoids had flattened epithelial cells expressing ISL1 and GATA3. In Fig. 6b, representative images of four biologically independent experiments (*n* = 4). Approximately 41.6% of day 9 bilaminoids had flattened epithelial cells expressing BLIMP1–tdTomato and TFAP2C–GFP. In Fig. 6c, representative images of three biologically independent experiments are shown (*n* = 3). Approximately 42.8% of D9 bilaminoids had CD34 and ERG double-positive cells.

In Extended Data Fig. 1a, representative images of two biologically independent experiments are shown (*n* = 2). In Extended Data Fig. 1b, bar charts show the mean value of two biologically independent experiments (*n* = 2). In Extended Data Fig. 1c, representative images of two biologically independent experiments are shown (*n* = 2). In Extended Data Fig. 1d, bar charts show the mean value of two biologically independent experiments (*n* = 2). In Extended Data Fig. 1e, representative images of three biologically independent experiments are shown (*n* = 3). In Extended Data Fig. 1f, typical results of two biologically independent experiments are shown (*n* = 2). In Extended Data Fig. 1g, bar charts show the mean value of two biologically independent experiments (*n* = 2). In Extended Data Fig. 1h, bar charts show the mean value of two biologically independent experiments (*n* = 2). In Extended Data Fig. 1i, typical results of two biologically independent experiments are shown (*n* = 2). In Extended Data Fig. 1j, bar charts show the mean value of two biologically independent experiments (*n* = 2). In Extended Data Fig. 1k, typical results of three biologically independent experiments are shown (*n* = 3). In Extended Data Fig. 1l, bar charts show the mean value of two biologically independent experiments (*n* = 2). In Extended Data Fig. 1m, typical results of three biologically independent experiments are shown (*n* = 3). In Extended Data Fig. 1n, bar charts show the mean value of three biologically independent experiments (*n* = 3). In Extended Data Fig. 2a, 5 independent cell lines were established, and bar charts show the mean value of *n* = 13 (electrophoration-1), *n* = 3 (electrophoration-2), *n* = 5 (electrophoration-3), *n* = 28 (electrophoration-4), *n* = 22 (electrophoration-5). In Extended Data Fig. 2b, bar charts show the mean value of two biologically independent experiments (*n* = 2). In Extended Data Fig. 2c, typical results of two biologically independent experiments are shown (*n* = 2). In Extended Data Fig. 2d, three independent cell lines were established, and bar charts show the mean value of three *n* = 6 (electrophoration-1), *n* = 2 (electrophoration-2), *n* = 2 (electrophoration-3) biologically independent experiments. In Extended Data Fig. 2e, typical results of two biologically independent experiments are shown (*n* = 2). In Extended Data Fig. 3b, typical results of two biologically independent experiments are shown (*n* = 2). In Extended Data Fig. 3c, typical results of two biologically independent experiments are shown (*n* = 2). In Extended Data Fig. 3d, representative images of two biologically independent experiments are shown (*n* = 2). In Extended Data Fig. 3e, typical results of two biologically independent experiments are shown (*n* = 2). In Extended Data Fig. 3f, bar charts show the mean value of two biologically independent experiments (*n* = 2). In Extended Data Fig. 3g, representative images of three biologically independent experiments (*n* = 3). In Extended Data Fig. 4b, bar charts show the mean value of two biologically independent experiments (*n* = 2). In Extended Data Fig. 4c, typical results of two biologically independent experiments are shown (*n* = 2). In Extended Data Fig. 4d, typical results of two biologically independent experiments are shown (*n* = 2). In Extended Data Fig. 4g, typical results of two biologically independent experiments are shown (*n* = 2). In Extended Data Fig. 4h, typical results of two biologically independent experiments are shown (*n* = 2). In Extended Data Fig. 4i, typical results of two biologically independent experiments are shown (*n* = 3). In Extended Data Fig. 4k, typical results of two biologically independent experiments are shown (*n* = 2). In Extended Data Fig. 4l, bar charts show the mean value of two biologically independent experiments (*n* = 2). In Extended Data Fig. 4m, typical results of two biologically independent experiments are shown (*n* = 2). In Extended Data Fig. 5a, typical results of two biologically independent experiments are shown (*n* = 2). In Extended Data Fig. 5b, bar charts show the mean value of two biologically independent experiments (*n* = 2). In Extended Data Fig. 5c, typical results of two biologically independent experiments are shown (*n* = 2). In Extended Data Fig. 5d, bar charts show the mean value of two biologically independent experiments (*n* = 2). In Extended Data Fig. 6a, representative images of three biologically independent experiments are shown (*n* = 3). In Extended Data Fig. 6b, long/short axes were measured from four biologically independent experiments (*n* = 4). In Extended Data Fig. 6d, the proportion of aggregates was measured from two biologically independent experiments (*n* = 2). In Extended Data Fig. 6e, representative images of three biologically independent experiments are shown (*n* = 3). In Extended Data Fig. 6f, the relative distance from the centre of aggregates was measured from two biologically independent experiments (*n* = 2). In Extended Data Fig. 6g, the distribution of Naive-GFP(G6-OE) in bilaminoids was measured from two biologically independent experiments (*n* = 2). In Extended Data Fig. 6h, a total of ten aggregates on each day was counted (*n* = 2). In Extended Data Fig. 6i, aggregate size was measured from two biologically independent experiments (*n* = 2). The number of total aggregates analysed for each group is shown at the top. In Extended Data Fig. 6j, bar charts show the mean value of two biologically independent experiments (*n* = 2). In Extended Data Fig. 6l, bar charts show the mean value of two biologically independent experiments (*n* = 2). In Extended Data Fig. 6m, representative images of two biologically independent experiments are shown (*n* = 2). In Extended Data Fig. 6n, the proportion of aggregates was measured from *n* = 6 (N + G6N), *n* = 5 (N + 7F), *n* = 4 (N + 4F), *n* = 5 (N + G6P), *n* = 4 (N + RACL), *n* = 4 (N + DE), *n* = 5 (P + G6N), *n* = 3 (P + 7F), *n* = 3 (P + 4F), *n* = 4 (P + G6P), *n* = 3 (P + RACL), *n* = 4 (P + DE), *n* = 6 (N(WT) + N(G6-OE)) biologically independent experiments. The number of aggregates analysed for each group is shown at the top. In Extended Data Fig. 6o, the efficiency of bilaminoid formation was measured from *n* = 6 (N + G6N), *n* = 5 (N + 7F), *n* = 6 (N(WT) + N(G6-OE)) biologically independent experiments. The number of aggregates analysed for each group is shown at the top. In Extended Data Fig. 6p, the efficiency of bilaminoid formation was measured from *n* = 6 (N + G6N), *n* = 5 (N + 7F), *n* = 6 (N(WT) + N(G6-OE)) biologically independent experiments. The number of aggregates analysed for each group is shown at the top. In Extended Data Fig. 6q, representative images of three biologically independent experiments are shown (*n* = 3). In Extended Data Fig. 6r, typical results of three biologically independent experiments are shown (*n* = 3). In Extended Data Fig. 6s, representative images of *n* = 3 (585B1) and *n* = 4 (1390G3) biologically independent experiments are shown. In Extended Data Fig. 6t, typical results of three biologically independent experiments are shown (*n* = 3). In Extended Data Fig. 7a, the cell number was measured from two biologically independent experiments (*n* = 2). A total of ten aggregates on each day was counted. In Extended Data Fig. 7b, the aggregate size was measured from two biologically independent experiments (*n* = 2). The number of total aggregates analysed for each group is shown at the top. In Extended Data Fig. 7c, representative images of three biologically independent experiments are shown (*n* = 3). In Extended Data Fig. 7d, the proportion of aggregates surrounded by nHyCs was measured from three biologically independent experiments (*n* = 3). The number of total aggregates analysed for each group is shown at the top. In Extended Data Fig. 7e, representative images of three biologically independent experiments are shown (*n* = 3). In Extended Data Fig. 7f, the efficiency of cavity formation and volume of the amniotic cavity of aggregates were measured from *n* = 5 (585B1) and *n* = 4 (1390G3) biologically independent experiments. The number of total aggregates analysed for each group is shown at the top. In Extended Data Fig. 7h, the cavity formation rate was measured from five biological replicates (*n* = 5). The number of total aggregates analysed for each group is shown at the top. In Extended Data Fig. 7i, the cavity formation rate was measured from two biological replicates (*n* = 2). The number of total aggregates analysed for each group is shown at the top. In Extended Data Fig. 7k, bar charts show the mean value of two biologically independent experiments (*n* = 2). In Extended Data Fig. 7l, typical results of three biologically independent experiments are shown (*n* = 3). In Extended Data Fig. 7m, bar charts show the mean value of two biologically independent experiments (*n* = 2). In Extended Data Fig. 7n, the aggregate size was measured from two biologically independent experiments (*n* = 2). The number of total aggregates analysed for each group is shown at the top. In Extended Data Fig. 7o, typical results of two biologically independent experiments are shown (*n* = 2). In Extended Data Fig. 7p, the bar charts show the mean value of two biologically independent experiments (*n* = 2). In Extended Data Fig. 7q, representative images of three biologically independent experiments are shown (*n* = 3). In Extended Data Fig. 7r, cell number was measured from two biologically independent experiments (*n* = 2). A total of 12 aggregates (WT + G6), 13 aggregates (WT + G6-Y) and 14 aggregates (WT-Y + G6) was counted. In Extended Data Fig. 7t, bar charts show the mean value of two biologically independent experiments (*n* = 2). In Extended Data Fig. 7u, the efficiency of bilaminoid formation was measured from six biologically independent experiments (*n* = 6). The number of aggregates analysed for each group is shown at the top. In Extended Data Fig. 7v, bar charts show the mean value of two biologically independent experiments (*n* = 2). In Extended Data Fig. 7w, the efficiency of cavity formation and volume of the amniotic cavity of aggregates were measured from two biologically independent experiments (*n* = 2). The number of aggregates analysed for each group is shown at the top. In Extended Data Fig. 7x, representative images of two biologically independent experiments are shown (*n* = 2). In Extended Data Fig. 7y, representative images of four biologically independent experiments are shown (*n* = 4). In Extended Data Fig. 8a, representative images of three biologically independent experiments are shown (*n* = 3). In Extended Data Fig. 8j, representative series of *z* sections images of three biologically independent experiments are shown (*n* = 3). In Extended Data Fig. 8k, angles were measured from *n* = 2 (LEFTY) and *n* = 2 (DKK1) biologically independent experiments. A total of 21 aggregates surrounded by nHyCs(G6-OE) and expressing LEFTY and T was counted. A total of 14 aggregates surrounded by nHyCs(G6-OE) and expressing DKK1 and T was counted. In Extended Data Fig. 8l, the proportion of T-expressing bilaminoids was counted from two biologically independent experiments (*n* = 2). The number of total aggregates analysed for each group is shown at the top. In Extended Data Fig. 8m, bar charts show the mean value of two biologically independent experiments (*n* = 2). In Extended Data Fig. 8n, bar charts show the mean value of two biologically independent experiments (*n* = 2). In Extended Data Fig. 9d, typical results of two biologically independent experiments are shown (*n* = 2). In Extended Data Fig. 9e, the bar charts show the mean value of two biologically independent experiments (*n* = 2). In Extended Data Fig. 9h, the bar charts show the mean value of two biologically independent experiments (*n* = 2). In Extended Data Fig. 10a, representative series of *z* sections images of three biologically independent experiments (*n* = 3). A total of 68% of day 9 bilaminoids had flattened epithelial cells expressing ISL1 and GATA3. In Extended Data Fig. 10b, representative images of three biologically independent experiments are shown (*n* = 3). In Extended Data Fig. 10c, representative images of three biologically independent experiments are shown (*n* = 3). In Extended Data Fig. 10d, representative images of three biologically independent experiments are shown (*n* = 3). In Extended Data Fig. 10e, typical results of three biologically independent experiments are shown (*n* = 3). In Extended Data Fig. 10g, representative images of two biologically independent experiments are shown (*n* = 2). In Extended Data Fig. 10j, representative images of four biologically independent experiments are shown (*n* = 4). In Extended Data Fig. 10k, representative images of *n* = 4 (7F) and *n* = 2 (G6) independent experiments are shown.

### Reporting summary

Further information on research design is available in the Nature Portfolio Reporting Summary linked to this article.

## Online content

Any methods, additional references, Nature Portfolio reporting summaries, source data, extended data, supplementary information, acknowledgements, peer review information; details of author contributions and competing interests; and statements of data and code availability are available at 10.1038/s41586-023-06871-2.

### Supplementary information

Supplementary Information Supplementary Figs. 1–3 and legends for Supplementary Tables 1–8.

Reporting Summary

Supplementary Table 1

Supplementary Table 2

Supplementary Table 3

Supplementary Table 4

Supplementary Table 5

Supplementary Table 6

Supplementary Table 7

Supplementary Table 8

### Source data

Source Data Fig. 2

Source Data Fig. 3

Source Data Fig. 4

Source Data Fig. 5

Source Data Extended Data Fig. 1

Source Data Extended Data Fig. 2

Source Data Extended Data Fig. 3

Source Data Extended Data Fig. 4

Source Data Extended Data Fig. 5

Source Data Extended Data Fig. 6

Source Data Extended Data Fig. 7

Source Data Extended Data Fig. 8

Source Data Extended Data Fig. 9

## Data Availability

All newly generated RNA-seq were deposited at the GEO under accession number GSE131747. Publicly available data used in this study were obtained from the following sources: GSE138012 (primitive endoderm)^23^; GSE52658 (ref. ^71^) and GSE75748 (ref. ^35^) (definitive endoderm); GSE144994 (naive PSC derived trophectoderm)^21^; GSE136447 (ref. ^5^), E-MTAB-3929 (ref. ^2^), GSE66507 (ref. ^3^), E-MTAB-9388 (ref. ^8^) and GSE171820 (ref. ^16^) (human embryo); GSE171820 (ref. ^16^), GSE134571 (ref. ^12^), GSE156596 (ref. ^14^), GSE150578 (ref. ^15^) and GSE177689 (ref. ^17^) (human embryo model); and GSE101074 (ATAC–seq data)^72^. Any other data and information are available on request. Full scan images for Extended Data Figs. 3b and 7o are provided in Supplementary Figs. 2 and 3. Source data are provided with this paper.

## Code availability

No custom code or algorithms were developed for the data analysis in this study.

## References

[1] Yan L (2013). Single-cell RNA-seq profiling of human preimplantation embryos and embryonic stem cells. Nat. Struct. Mol. Biol..

[2] Petropoulos S (2016). Single-cell RNA-seq reveals lineage and X chromosome dynamics in human preimplantation embryos. Cell.

[3] Blakeley P (2015). Defining the three cell lineages of the human blastocyst by single-cell RNA-seq. Development.

[4] Zhou F (2019). Reconstituting the transcriptome and DNA methylome landscapes of human implantation. Nature.

[5] Xiang L (2020). A developmental landscape of 3D-cultured human pre-gastrulation embryos. Nature.

[6] Deglincerti A (2016). Self-organization of the in vitro attached human embryo. Nature.

[7] Stirparo GG (2018). Integrated analysis of single-cell embryo data yields a unified transcriptome signature for the human pre-implantation epiblast. Development.

[8] Tyser, R. C. V. et al. Single-cell transcriptomic characterization of a gastrulating human embryo. *Nature*10.1038/s41586-021-04158-y (2021). 10.1038/s41586-021-04158-y PMC7615353 34789876

[9] Warmflash A, Sorre B, Etoc F, Siggia ED, Brivanlou AH (2014). A method to recapitulate early embryonic spatial patterning in human embryonic stem cells. Nat. Methods.

[10] Shahbazi MN (2017). Pluripotent state transitions coordinate morphogenesis in mouse and human embryos. Nature.

[11] Martyn I, Kanno TY, Ruzo A, Siggia ED, Brivanlou AH (2018). Self-organization of a human organizer by combined Wnt and Nodal signalling. Nature.

[12] Zheng Y (2019). Controlled modelling of human epiblast and amnion development using stem cells. Nature.

[13] Moris N (2020). An in vitro model of early anteroposterior organization during human development. Nature.

[14] Liu, X. et al. Modelling human blastocysts by reprogramming fibroblasts into iBlastoids. *Nature*10.1038/s41586-021-03372-y (2021). 10.1038/s41586-021-03372-y 33731926

[15] Yu L (2021). Blastocyst-like structures generated from human pluripotent stem cells. Nature.

[16] Yanagida, A. et al. Naive stem cell blastocyst model captures human embryo lineage segregation. *Cell Stem Cell*10.1016/j.stem.2021.04.031 (2021). 10.1016/j.stem.2021.04.031 PMC8189436 33957081

[17] Kagawa, H. et al. Human blastoids model blastocyst development and implantation. *Nature*10.1038/s41586-021-04267-8 (2021). 10.1038/s41586-021-04267-8 PMC8791832 34856602

[18] Takashima Y (2014). Resetting transcription factor control circuitry toward ground-state pluripotency in human. Cell.

[19] Theunissen TW (2014). Systematic identification of culture conditions for induction and maintenance of naive human pluripotency. Cell Stem Cell.

[20] Guo G (2017). Epigenetic resetting of human pluripotency. Development.

[21] Io S (2021). Capturing human trophoblast development with naive pluripotent stem cells in vitro. Cell Stem Cell.

[22] Guo G (2021). Human naive epiblast cells possess unrestricted lineage potential. Cell Stem Cell.

[23] Linneberg-Agerholm, M. et al. Naive human pluripotent stem cells respond to Wnt, Nodal and LIF signalling to produce expandable naive extra-embryonic endoderm. *Development*10.1242/dev.180620 (2019). 10.1242/dev.180620 31740534

[24] Schrode N, Saiz N, Di Talia S, Hadjantonakis AK (2014). GATA6 levels modulate primitive endoderm cell fate choice and timing in the mouse blastocyst. Dev. Cell.

[25] Fujikura J (2002). Differentiation of embryonic stem cells is induced by GATA factors. Genes Dev..

[26] McDonald AC, Biechele S, Rossant J, Stanford WL (2014). Sox17-mediated XEN cell conversion identifies dynamic networks controlling cell-fate decisions in embryo-derived stem cells. Cell Rep..

[27] Kataoka H (1997). Expressions of *PDGF receptor alpha*, *c-Kit* and *Flk1* genes clustering in mouse chromosome 5 define distinct subsets of nascent mesodermal cells. Dev. Growth Differ..

[28] Murry CE, Keller G (2008). Differentiation of embryonic stem cells to clinically relevant populations: lessons from embryonic development. Cell.

[29] Nakamura T (2016). A developmental coordinate of pluripotency among mice, monkeys and humans. Nature.

[30] Cho LT (2012). Conversion from mouse embryonic to extra-embryonic endoderm stem cells reveals distinct differentiation capacities of pluripotent stem cell states. Development.

[31] Artus J, Panthier JJ, Hadjantonakis AK (2010). A role for PDGF signaling in expansion of the extra-embryonic endoderm lineage of the mouse blastocyst. Development.

[32] Vrij, E. J. et al. A pendulum of induction between the epiblast and extra-embryonic endoderm supports post-implantation progression. *Development*10.1242/dev.192310 (2022). 10.1242/dev.192310 PMC9534490 35993866

[33] Artus J, Piliszek A, Hadjantonakis AK (2011). The primitive endoderm lineage of the mouse blastocyst: sequential transcription factor activation and regulation of differentiation by Sox17. Dev. Biol..

[34] Anderson, K. G. V. et al. Insulin fine-tunes self-renewal pathways governing naive pluripotency and extra-embryonic endoderm. *Nat. Cell Biol.*10.1038/ncb3617 (2017). 10.1038/ncb3617 28945231

[35] Chu LF (2016). Single-cell RNA-seq reveals novel regulators of human embryonic stem cell differentiation to definitive endoderm. Genome Biol..

[36] Rivron NC (2018). Blastocyst-like structures generated solely from stem cells. Nature.

[37] Shahbazi MN (2016). Self-organization of the human embryo in the absence of maternal tissues. Nat. Cell Biol..

[38] Roode M (2012). Human hypoblast formation is not dependent on FGF signalling. Dev. Biol..

[39] Riedl J (2008). Lifeact: a versatile marker to visualize F-actin. Nat. Methods.

[40] Di Stefano B (2018). Reduced MEK inhibition preserves genomic stability in naive human embryonic stem cells. Nat. Methods.

[41] Meistermann, D. et al. Integrated pseudotime analysis of human pre-implantation embryo single-cell transcriptomes reveals the dynamics of lineage specification. *Cell Stem Cell*10.1016/j.stem.2021.04.027 (2021). 10.1016/j.stem.2021.04.027 34004179

[42] Niwa H, Burdon T, Chambers I, Smith A (1998). Self-renewal of pluripotent embryonic stem cells is mediated via activation of STAT3. Genes Dev..

[43] Zhao, C. et al. Reprogrammed iBlastoids contain amnion-like cells but not trophectoderm. Preprint at *bioRxiv*10.1101/2021.05.07.442980 (2021).

[44] Varlet I, Collignon J, Robertson EJ (1997). *Nodal* expression in the primitive endoderm is required for specification of the anterior axis during mouse gastrulation. Development.

[45] Chazaud C, Yamanaka Y, Pawson T, Rossant J (2006). Early lineage segregation between epiblast and primitive endoderm in mouse blastocysts through the Grb2-MAPK pathway. Dev. Cell.

[46] Colognato H, Yurchenco PD (2000). Form and function: the laminin family of heterotrimers. Dev. Dyn..

[47] Bedzhov I, Zernicka-Goetz M (2014). Self-organizing properties of mouse pluripotent cells initiate morphogenesis upon implantation. Cell.

[48] Rostovskaya M, Andrews S, Reik W, Rugg-Gunn PJ (2022). Amniogenesis occurs in two independent waves in primates. Cell Stem Cell.

[49] Rouillard, A. D. et al. The harmonizome: a collection of processed datasets gathered to serve and mine knowledge about genes and proteins. *Database*10.1093/database/baw100 (2016). 10.1093/database/baw100 PMC4930834 27374120

[50] Weatherbee, B. A. T. et al. Pluripotent stem cell-derived model of the post-implantation human embryo. *Nature*10.1038/s41586-023-06368-y (2023). 10.1038/s41586-023-06368-y PMC10584688 37369347

[51] Pedroza, M. et al. Self-patterning of human stem cells into post-implantation lineages. *Nature*10.1038/s41586-023-06354-4 (2023). 10.1038/s41586-023-06354-4 PMC10584676 37369348

[52] Liu, L. et al. Modeling post-implantation stages of human development into early organogenesis with stem-cell-derived peri-gastruloids. *Cell*10.1016/j.cell.2023.07.018 (2023). 10.1016/j.cell.2023.07.018 37478861

[53] Oldak, B. et al. Complete human day 14 post-implantation embryo models from naive ES cells. *Nature* **622**, 562–573 (2023). 10.1038/s41586-023-06604-5 PMC10584686 37673118

[54] Sasaki K (2015). Robust in vitro induction of human germ cell fate from pluripotent stem cells. Cell Stem Cell.

[55] Yamashiro C (2018). Generation of human oogonia from induced pluripotent stem cells in vitro. Science.

[56] Ying Q-L, Stavridis M, Griffiths D, Li M, Smith A (2003). Conversion of embryonic stem cells into neuroectodermal precursors in adherent monoculture. Nat. Biotechnol..

[57] Hotta A (2009). Isolation of human iPS cells using EOS lentiviral vectors to select for pluripotency. Nat. Methods.

[58] Yu L (2021). Derivation of intermediate pluripotent stem cells amenable to primordial germ cell specification. Cell Stem Cell.

[59] Kim SI (2016). Inducible transgene expression in human iPS cells using versatile all-in-one piggyBac transposons. Methods Mol. Biol..

[60] Thomson JA, Kalishman J, Hearn JP (1994). Nonsurgical uterine stage preimplantation embryo collection from the common marmoset. J. Med. Primatol..

[61] Sun T (2019). Blockade of a laminin-411-notch axis with CRISPR/Cas9 or a nanobioconjugate inhibits glioblastoma growth through tumor-microenvironment cross-talk. Cancer Res..

[62] Pham TXA (2022). Modeling human extraembryonic mesoderm cells using naive pluripotent stem cells. Cell Stem Cell.

[63] Pagliuca FW (2014). Generation of functional human pancreatic beta cells in vitro. Cell.

[64] Io S, Iemura Y, Takashima Y (2021). Optimized protocol for naive human pluripotent stem cell-derived trophoblast induction. STAR Protoc..

[65] Kumar B (2022). Polycomb repressive complex 2 shields naive human pluripotent cells from trophectoderm differentiation. Nat. Cell Biol..

[66] Zheng Y (2022). Single-cell analysis of embryoids reveals lineage diversification roadmaps of early human development. Cell Stem Cell.

[67] Martin, M. Cutadapt removes adapter sequences from high-throughput sequencing reads. *EMBnet J.*10.14806/ej.17.1.200 (2011).

[68] Kim D (2013). TopHat2: accurate alignment of transcriptomes in the presence of insertions, deletions and gene fusions. Genome Biol..

[69] Frankish A (2019). GENCODE reference annotation for the human and mouse genomes. Nucleic Acids Res..

[70] Trapnell C (2010). Transcript assembly and quantification by RNA-seq reveals unannotated transcripts and isoform switching during cell differentiation. Nat. Biotechnol..

[71] Loh KM (2014). Efficient endoderm induction from human pluripotent stem cells by logically directing signals controlling lineage bifurcations. Cell Stem Cell.

[72] Pastor WA (2018). TFAP2C regulates transcription in human naive pluripotency by opening enhancers. Nat. Cell Biol..

[73] Daily K, Patel VR, Rigor P, Xie X, Baldi P (2011). MotifMap: integrative genome-wide maps of regulatory motif sites for model species. BMC Bioinform..

