# Publisher Correction: Hypoblast from human pluripotent stem cells regulates epiblast development

**DOI:** 10.1038/s41586-024-07166-w

**Published:** 2024-02-12

**Authors:** Takumi Okubo, Nicolas Rivron, Mio Kabata, Hideki Masaki, Keiko Kishimoto, Katsunori Semi, May Nakajima-Koyama, Haruko Kunitomi, Belinda Kaswandy, Hideyuki Sato, Hiromitsu Nakauchi, Knut Woltjen, Mitinori Saitou, Erika Sasaki, Takuya Yamamoto, Yasuhiro Takashima

**Affiliations:** 1 Center for iPS Cell Research and Application, Kyoto University, Kyoto, Japan; 2 Institute of Molecular Biotechnology of the Austrian Academy of Sciences (IMBA), Vienna BioCenter (VBC), Vienna, Austria; 3 Institute of Medical Science, University of Tokyo, Tokyo, Japan; 4 Advanced Research Institute, Tokyo Medical and Dental University, Tokyo, Japan; 5 Central Institute for Experimental Animals, Kawasaki, Japan; 6 Institute for Stem Cell Biology and Regenerative Medicine, Stanford University School of Medicine, Stanford, CA USA; 7 Institute for the Advanced Study of Human Biology (WPI-ASHBi), Kyoto University, Kyoto, Japan; 8 Department of Anatomy and Cell Biology, Graduate School of Medicine, Kyoto University, Kyoto, Japan; 9 Medical-risk Avoidance Based on iPS Cells Team, RIKEN Center for Advanced Intelligence Project (AIP), Kyoto, Japan

**Keywords:** Embryonic germ cells, Embryonic induction

Correction to: *Nature* 10.1038/s41586-023-06871-2 Published online 5 December 2023

In the version of the article initially published, in Fig. 2i, “FGFi/TGF-βi/(BMP)” previously read “FGFi/TGF-αi/(BMP)”. This has been corrected in the HTML and PDF versions of the article.

