# Supplementary material for: Hypoblast from human pluripotent stem cells regulates epiblast development
Source: Nature. 2023 Dec 5;626(7998):357–66. doi: 10.1038/s41586-023-06871-2 (PMC10849967; doi:10.1038/s41586-023-06871-2)
Supplement: Supplementary file 1 — Supplementary Figs. 1–3 and legends for Supplementary Tables 1–8. [file 41586_2023_6871_MOESM1_ESM.pdf]

nature portfolio

https://doi.org/10.1038/s41586-023-06871-2

Supplementary information

Hypoblast from human pluripotent stem cells regulates epiblast development

In the format provided by the authors and unedited

Supplementary Information (SI) Guide

I. **Supplementary Table Legends**

II. **Supplementary Figure 1-3 and Legends**

Supplementary Table 1

The expression matrix and sample information of Bulk RNA-seq data

The gene expression matrix and sample information of this and published studies are listed.

Supplementary Table 2

PC1, PC2, and PC3 loadings of PCA (Fig. 1e)

Supplementary Table 3

Gene sets related to Fig. 2d, 2h, and Extended Data Fig. 3i and 5g

Differentially expressed genes (DEGs) between epiblast and hypoblast assigned by Stirparo et al. (Fig. 2d), re-annotation of human cultured embryo data (Xiang et al., 2020) (Fig. 2h), 202 transcriptional factors extracted from DEGs identified by Stirparo et al. (Extended Data Fig. 3i), gene sets assigned by Nakamura et al. (2016) (Extended Data Fig. 5g).

Supplementary Table 4

Single-cell RNA-seq data of bilaminoids on D6 and D9

The expression matrix of single-cell RNA-seq data of bilaminoids on D6 and D9 and cell annotation and clustering of each cell on D6 are shown.

Supplementary Table 5

Correlation coefficients between trophoblast and amnion

Ontogenic gene sets between trophoblast and amnion were determined by Zheng et al. (2022).

Supplementary Table 6

DEGs between hypoblast, epiblast, primitive streak, mesoderm, and amnion

The top 50 genes were identified using the Seurat FindMarkers function (test.use = wilcox). The *p* values were adjusted for FDR with the Benjamini–Hochberg method.

Supplementary Table 7

A summary of our bilaminoid model and recent reported models

Recent stem cell-based post-implantation models using *in vitro* epiblast-like and hypoblast-like cells[50, 51, 52, 53] and our model are summarized. The developmental window of our model is from blastocyst to peri-gastrulation by starting with naïve hPSCs that reflect day 5 epiblast [7] of the blastocyst and form hypoblast-like cells within two days,

thereby reflecting a late blastocyst-stage hypoblast. Since PSCs cultured in RSeT medium and expanded pluripotent stem cells (EPSCs) show gene expression patterns resembling the post-implantation stages rather than pre-implantation[22,58], the three models [50, 51, 52] using these PSCs skipped the blastocyst stage and model development following implantation. The other model, SEM, established under HENSM condition [53], also starts from the early post-implantation stage because extraembryonic mesoderm (ExMC) [62] that does not exist in the blastocyst cell components is mixed to generate this model. Therefore, the developmental window modelled in our study is wider and distinguishes our work substantially from the other works.

A critical feature of our model is that it precisely matches natural developmental sequence and timing. Upon co-culture of naïve PSCs reflecting the day 5 epiblast, bilaminoids formed within two days and showed characteristic signs of the pre-gastrulation (day 12) embryo after 6 days, faithfully matching in vivo development: Carnegie stage (CS)3 at D0, CS4 at D2, CS5a at D4, CS5c at D6, and CS6a at D9. The relatively closer reconstruction of the sequence and timing allowed us to study IL6 signaling between the trophoblast and epiblast, the lamina deposition by the hypoblast that dictated polarization of the epiblast, and the functional effect of OTX2 and DKK1 on the expression of T that has yet to be recognized by other models.

The efficiency of generating our bilaminoid model using naïve PSCs is comparable to other models using RSeT and EPSCs [51, 52]. However, since the precise evaluation criteria differ between studies, further comparison will be required. Indeed, if we applied the stringent criteria of bilaminoids based on each aggregate’s capacity to have its epiblast-like cells completely surrounded by nHyC and to form a single complete amniotic-like cavity, the efficiency of bilaminoids generated by the mixture of naïve PSCs and sorted G6-nHyC or sorted 7F-nHyC are 16.1 and 12.1%, respectively.

Since our model was cultured in serum-free conditions, it is likely to be useful for observing and manipulating developmental mechanisms, such as signaling pathways and transcriptional networks. Functional assays with genetic modifications are almost impossible in human embryos. In bilaminoids, several lineage-specific gene modifications were performed and novel interactions between lineages were discovered.

Supplementary Table 8

RT-qPCR primers and antibodies used in this study

Supplementary Figure 1

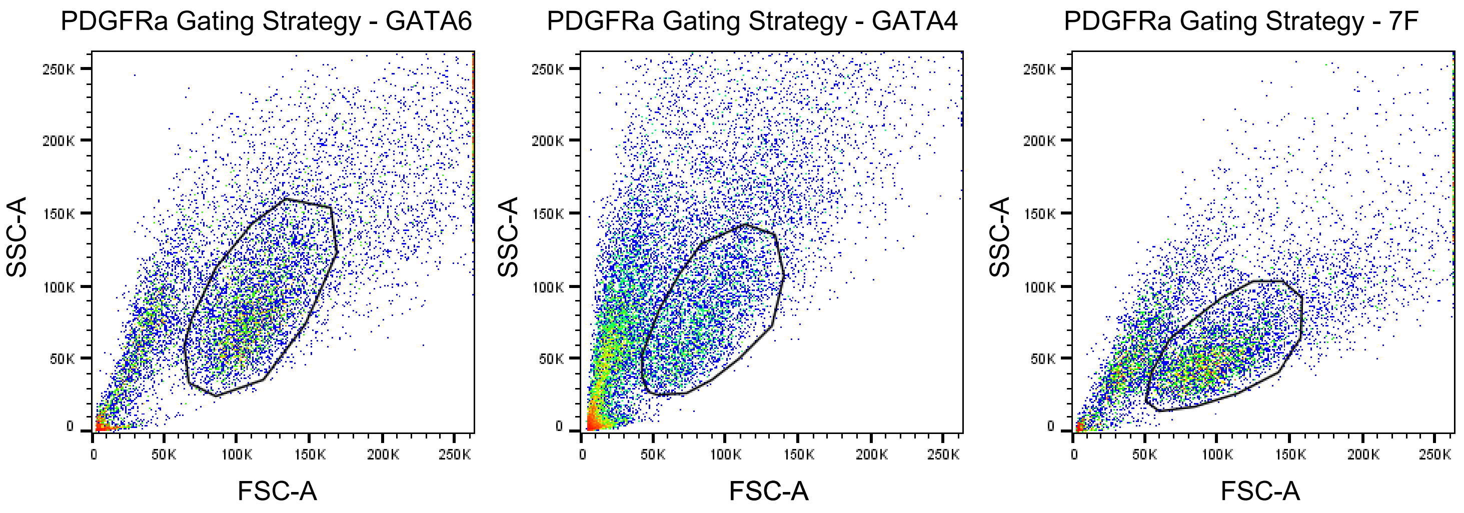

Supplementary Figure 1

Gating strategy of Flow Cytometry. Gating strategy for FSC and SSC used in this manuscript are shown.

Supplementary Figure 2

Extended Date Fig.3b

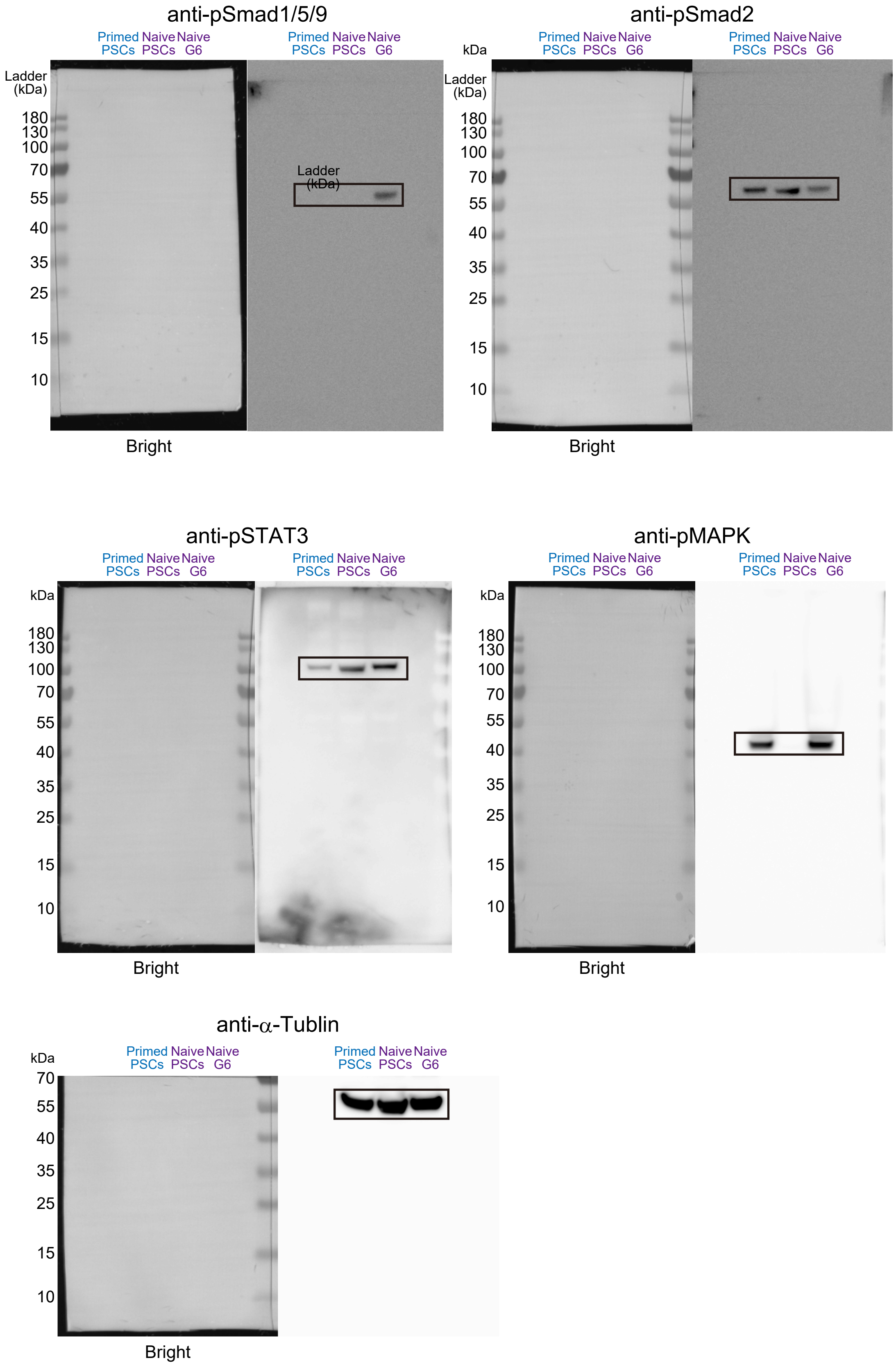

Supplementary Figure 2

Uncropped western blot images of Extended Data Fig. 3b

Supplementary Figure 3

Extended Date Fig.7o

anti-pSTAT3

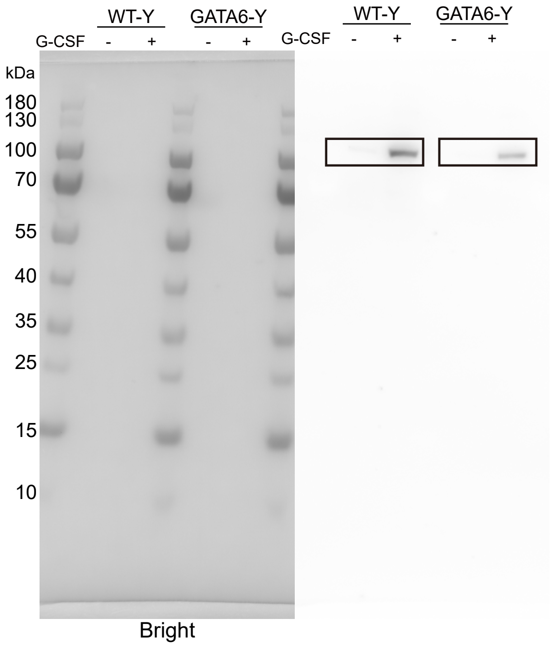

anti-STAT3

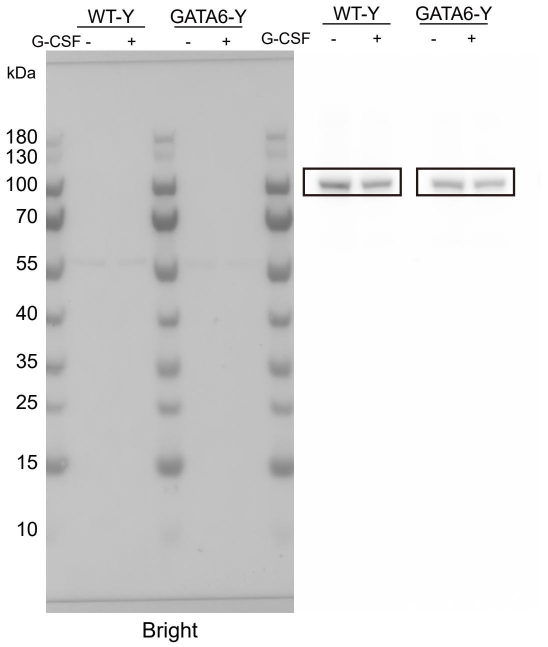

anti-α-Tublin

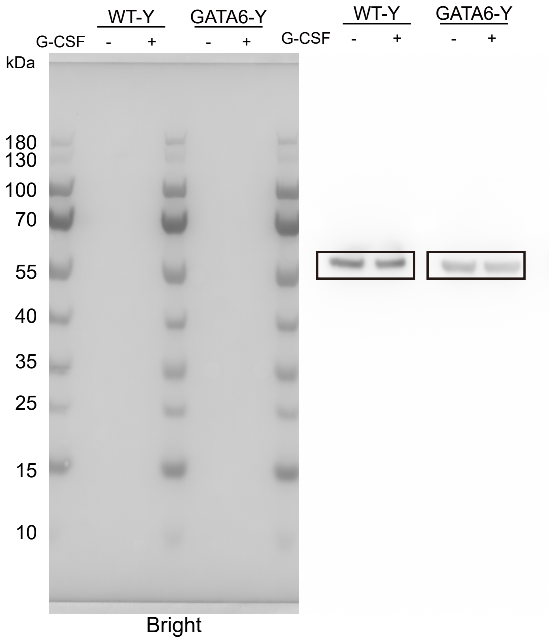

Supplementary Figure 3

Uncropped western blot images of Extended Data Fig. 7o
