# Supplementary material for: Hypoblast from human pluripotent stem cells regulates epiblast development
Source: Nature. 2023 Dec 5;626(7998):357–66. doi: 10.1038/s41586-023-06871-2 (PMC10849967; doi:10.1038/s41586-023-06871-2)
Supplement: Supplementary file 2 — Reporting Summary [file 41586_2023_6871_MOESM2_ESM.pdf]

nature research

Corresponding author(s): Yasuhiro Takashima and Takuya Yamamoto

Last updated by author(s): Oct 20, 2023

Reporting Summary

Nature Research wishes to improve the reproducibility of the work that we publish. This form provides structure for consistency and transparency in reporting. For further information on Nature Research policies, see our Editorial Policies and the Editorial Policy Checklist.

Statistics

For all statistical analyses, confirm that the following items are present in the figure legend, table legend, main text, or Methods section.

n/a	Confirmed	
☐	☒	The exact sample size (n) for each experimental group/condition, given as a discrete number and unit of measurement
☐	☒	A statement on whether measurements were taken from distinct samples or whether the same sample was measured repeatedly
☐	☒	The statistical test(s) used AND whether they are one- or two-sided<br>*Only common tests should be described solely by name; describe more complex techniques in the Methods section.*
☐	☒	A description of all covariates tested
☐	☒	A description of any assumptions or corrections, such as tests of normality and adjustment for multiple comparisons
☐	☒	A full description of the statistical parameters including central tendency (e.g. means) or other basic estimates (e.g. regression coefficient) AND variation (e.g. standard deviation) or associated estimates of uncertainty (e.g. confidence intervals)
☐	☒	For null hypothesis testing, the test statistic (e.g. F, t, r) with confidence intervals, effect sizes, degrees of freedom and P value noted<br>*Give P values as exact values whenever suitable.*
☒	☐	For Bayesian analysis, information on the choice of priors and Markov chain Monte Carlo settings
☐	☒	For hierarchical and complex designs, identification of the appropriate level for tests and full reporting of outcomes
☒	☐	Estimates of effect sizes (e.g. Cohen's d, Pearson's r), indicating how they were calculated

Our web collection on statistics for biologists contains articles on many of the points above.

Software and code

Policy information about availability of computer code

Data collection	Novaseq 6000, HiSeq 4000, NextSeq 500, Nextseq 2000 High Output v2 Kit (75 Cycles, FC-404-2005) (Illumina); TCS SP8 (Leica, Wetzlar, Germany); LSM710, LSM900, Celldiscover7 (Zeiss, Oberkochen, Germany); QuantStudio3, QuantStudio12K (Thermo Fisher Scientific); Infinite200PRO (TECAN); ImageQuant LAS 4000, Amersham™ImageQuant 800 (Cytiva); LSR Fortessa, FACS Aria II (BD)
Data analysis	Data were analyzed using: GraphPad Prism (v9.4.1, v10.0.3), FlowJo (v10.7.2), Imaris (v10.0.0), QuantStudio Design & Analysis(v1.4.1), cutadapt (v1.15), TopHat2, GENCODE (v27), DeSeq2(v1.22.2), cufflinks (v2.2.1), R (v3.3.2), R (v3.5.1), Cell Ranger pipeline (v3.1.0), STAR aligner (v2.5.1b), RSEM (v1.3.1), R Seurat package (v.4.0.4), STAR (v2.5.1b), STAR (v2.7.8a).

For manuscripts utilizing custom algorithms or software that are central to the research but not yet described in published literature, software must be made available to editors and reviewers. We strongly encourage code deposition in a community repository (e.g. GitHub). See the Nature Research guidelines for submitting code & software for further information.

Data

Policy information about availability of data

All manuscripts must include a data availability statement. This statement should provide the following information, where applicable:

- Accession codes, unique identifiers, or web links for publicly available datasets
- A list of figures that have associated raw data
- A description of any restrictions on data availability

All newly generated RNA-seq were deposited in the Gene Expression Omnibus under accession number GSE131747.
Public data
Primitive endoderm GSE138012 (Linnerberg et al., Development 2019).
Definitive endoderm GSE52658 (Loh et al., Cell Stem Cell 2014), GSE75748 (Chu et al., Genome Biology 2016).

Naive PSC derived trophectoderm GSE144994 (Io et al., Cell Stem Cell 2021).
Human embryo GSE136447 (Xiang et al., Nature 2020).
Human embryo E-MTAB-3929 (Petropoulos et al., Cell 2016).
Human embryo GSE66507 (Blakeley et al., Development 2015).
Human embryo E-MTAB-9388 (Tyser et al., Nature 2021).
Human embryo and human embryo model GSE171820 (Yanagida et al., Cell stem cell 2021).
Human embryo model GSE134571 (Zheng et al., Nature 2019).
Human embryo model GSE156596 (Liu et al., Nature 2021).
Human embryo model GSE150578 (Yu et al., Nature 2021).
Human embryo model GSE177689 (Kagawa et al., Nature 2021).
ATAC-seq data, GSE101074 (Pastor et al., Nature Cell Biology 2018)

Field-specific reporting

Please select the one below that is the best fit for your research. If you are not sure, read the appropriate sections before making your selection.

☒ Life sciences ☐ Behavioural & social sciences ☐ Ecological, evolutionary & environmental sciences

For a reference copy of the document with all sections, see nature.com/documents/nr-reporting-summary-flat.pdf

Life sciences study design

All studies must disclose on these points even when the disclosure is negative.

Sample size	No statistical methods were used to predetermine sample size. Sample sizes were determined based on similar studies performed on stem cell-based model (ex. Zheng et al, Nature, 2019; Moris et al, Nature, 2021; Kagawa et at Nature 2022)
Data exclusions	No data were excluded.
Replication	All experiments were conducted with at least two biologically independent experiments. Exact number of experiments and samples are indicated in figures, figure legends and the section of Statistic and Reproducibility in the Methods.
Randomization	Samples were allocated based on the corresponding test conditions.
Blinding	Investigators were not blinded to group allocation during data collection and analysis, as these were nonsubjective.

Behavioural & social sciences study design

All studies must disclose on these points even when the disclosure is negative.

Study description	*Briefly describe the study type including whether data are quantitative, qualitative, or mixed-methods (e.g. qualitative cross-sectional, quantitative experimental, mixed-methods case study).*
Research sample	*State the research sample (e.g. Harvard university undergraduates, villagers in rural India) and provide relevant demographic information (e.g. age, sex) and indicate whether the sample is representative. Provide a rationale for the study sample chosen. For studies involving existing datasets, please describe the dataset and source.*
Sampling strategy	*Describe the sampling procedure (e.g. random, snowball, stratified, convenience). Describe the statistical methods that were used to predetermine sample size OR if no sample-size calculation was performed, describe how sample sizes were chosen and provide a rationale for why these sample sizes are sufficient. For qualitative data, please indicate whether data saturation was considered, and what criteria were used to decide that no further sampling was needed.*
Data collection	*Provide details about the data collection procedure, including the instruments or devices used to record the data (e.g. pen and paper, computer, eye tracker, video or audio equipment) whether anyone was present besides the participant(s) and the researcher, and whether the researcher was blind to experimental condition and/or the study hypothesis during data collection.*
Timing	*Indicate the start and stop dates of data collection. If there is a gap between collection periods, state the dates for each sample cohort.*
Data exclusions	*If no data were excluded from the analyses, state so OR if data were excluded, provide the exact number of exclusions and the rationale behind them, indicating whether exclusion criteria were pre-established.*
Non-participation	*State how many participants dropped out/declined participation and the reason(s) given OR provide response rate OR state that no participants dropped out/declined participation.*
Randomization	*If participants were not allocated into experimental groups, state so OR describe how participants were allocated to groups, and if allocation was not random, describe how covariates were controlled.*

Ecological, evolutionary & environmental sciences study design

All studies must disclose on these points even when the disclosure is negative.

Study description	*Briefly describe the study. For quantitative data include treatment factors and interactions, design structure (e.g. factorial, nested, hierarchical), nature and number of experimental units and replicates.*
Research sample	*Describe the research sample (e.g. a group of tagged Passer domesticus, all Stenocereus thurberi within Organ Pipe Cactus National Monument), and provide a rationale for the sample choice. When relevant, describe the organism taxa, source, sex, age range and any manipulations. State what population the sample is meant to represent when applicable. For studies involving existing datasets, describe the data and its source.*
Sampling strategy	*Note the sampling procedure. Describe the statistical methods that were used to predetermine sample size OR if no sample-size calculation was performed, describe how sample sizes were chosen and provide a rationale for why these sample sizes are sufficient.*
Data collection	*Describe the data collection procedure, including who recorded the data and how.*
Timing and spatial scale	*Indicate the start and stop dates of data collection, noting the frequency and periodicity of sampling and providing a rationale for these choices. If there is a gap between collection periods, state the dates for each sample cohort. Specify the spatial scale from which the data are taken*
Data exclusions	*If no data were excluded from the analyses, state so OR if data were excluded, describe the exclusions and the rationale behind them, indicating whether exclusion criteria were pre-established.*
Reproducibility	*Describe the measures taken to verify the reproducibility of experimental findings. For each experiment, note whether any attempts to repeat the experiment failed OR state that all attempts to repeat the experiment were successful.*
Randomization	*Describe how samples/organisms/participants were allocated into groups. If allocation was not random, describe how covariates were controlled. If this is not relevant to your study, explain why.*
Blinding	*Describe the extent of blinding used during data acquisition and analysis. If blinding was not possible, describe why OR explain why blinding was not relevant to your study.*

Did the study involve field work? ☐ Yes ☐ No

Field work, collection and transport

Field conditions	*Describe the study conditions for field work, providing relevant parameters (e.g. temperature, rainfall).*
Location	*State the location of the sampling or experiment, providing relevant parameters (e.g. latitude and longitude, elevation, water depth).*
Access & import/export	*Describe the efforts you have made to access habitats and to collect and import/export your samples in a responsible manner and in compliance with local, national and international laws, noting any permits that were obtained (give the name of the issuing authority, the date of issue, and any identifying information).*
Disturbance	*Describe any disturbance caused by the study and how it was minimized.*

Reporting for specific materials, systems and methods

We require information from authors about some types of materials, experimental systems and methods used in many studies. Here, indicate whether each material, system or method listed is relevant to your study. If you are not sure if a list item applies to your research, read the appropriate section before selecting a response.

Materials & experimental systems

n/a	Involved in the study	
☐	☒	Antibodies
☐	☒	Eukaryotic cell lines
☒	☐	Palaeontology and archaeology
☐	☒	Animals and other organisms
☒	☐	Human research participants
☒	☐	Clinical data
☒	☐	Dual use research of concern

Methods

n/a	Involved in the study	
☒	☐	ChIP-seq
☐	☒	Flow cytometry
☒	☐	MRI-based neuroimaging

Antibodies

Antibodies used

All antibodies used in this study are commercial and described in Supplymentary table 7.

Rabbit monoclonal anti-GATA6 (clone D61E4) Cell Signaling Technology Cat# 5851
Mouse monoclonal anti-GATA6 (clone 222228) R&D systems Cat# MAB1700
Goar polyclonal anti-GATA6 R&D systems Cat# AF1700
Goat polyclonal anti-GATA4 Santa Cruz Biotechnology Cat# sc-1237
Rat monoclonal anti-GATA4 (clone eBioEvan) Thermo Fisher Scientific Cat# 14-9980-82
Goat polyclonal anti-SOX17 R&D systems Cat# AF1924
Rabbit monoclonal anti-FOXA2 (clone D56D6) Cell Signaling Technology Cat# 8186
Mouse monoclonal anti-NANOG eBioscience (clone hNanog.2) Cat# 14-5769-82
Mouse monoclonal anti-NANOG eBioscience (clone hNanog.2) Cat# 14-5768-80
Mouse monoclonal anti-Oct4 Santa Cruz Biotechnology (clone C-10) Cat# sc-5279
Goat polyclonal anti-Oct4 Santa Cruz Biotechnology Cat# sc-8628
Rabbit monoclonal anti-Oct4 (clone C30A3) Cell Signaling Technology Cat# 2840
Rat monoclonal anti-GFP (clone GF090R) Nacalai Tesque Cat# 04404-84
Rabbit polyclonal anti-DsRed Takara Cat# 632496
Rabbit polyclonal anti-PARD6B (PAR6) Santa Cruz Biotechnology Cat# sc-67393
Mouse monoclonal anti-PARD6B(PAR6) (clone B-10) Santa Cruz Biotechnology Cat# sc-166405
Mouse monoclonal anti- PODXL (clone 222328) R&D systems Cat# MAB1658
Mouse monoclonal anti- PKCζ (aPKC) (clone H-1) Santa Cruz Biotechnology Cat# sc-17781
Goat polyclonal anti-Brachyury (T) R&D systems Cat# AF2085
Rabbit monoclonal anti-Brachyury (T) (clone D2Z3J) Cell Signaling Technology Cat# 81694
Mouse monoclonal anti-OTX2 (clone D-8) Santa Cruz Biotechnology Cat# sc-514195
Goat polyclonal anti-Lefty R&D systems Cat# AF746
Rabbit monoclonal anti-DKK1 (clone D5V6L) Cell Signaling Technology Cat# 48367
Rabbit polyclonal anti-Laminin Abcam Cat# ab11575
Rabbit polyclonal anti-Laminin beta-1 Thermo Fisher Scientific Cat# PA5-27271
Rabbit polyclonal anti-KLF17 Atlas Antibodies Cat# HPA024629
Goat polyclonal anti-GATA3 R&D systems Cat# AF2605
Mouse monoclonal anti-AP2alpha (TFAP2A) (clone 3B5) Santa Cruz Biotechnology Cat# sc-12726
Mouse monoclonal anti-ISL1&ISL2 (clone 39.4D5) DSHB Cat# 39.4D5
Goat polyclonal anti-Islet-1(ISL1) R&D systems Cat# AF1837
Rat monoclonal anti-CD34 abcam (clone MEC 14.7) Cat# ab8158
Rabbit monoclonal anti-ERG (clone A7L1G) Cell Signaling Technology Cat# 97249
Rabbit polyclonal anti-GATA2 NOVUS Cat# NBP1-82581
Mouse monoclonal anti-a-tubulin (clone DM1A) Abcam Cat# ab7291
Rabbit polyclonal anti- pSMAD1/5/9 Cell Signaling Technology Cat# 9511
Rabbit monoclonal anti- pSMAD2 (clone 138D4) Cell Signaling Technology Cat# 3108
Rabbit polyclonal anti- pSTAT3 Cell Signaling Technology Cat# 9131
Mouse monoclonal anti-STAT3 (clone 84/Stat3) BD Cat# 610189
Rabbit monoclonal anti-pMAPK (clone 20G11) Cell Signaling Technology Cat# 4376
Biotin goat polyclonal anti-PDGFRA R&D systems Cat# BAF322
Biotin human monoclonal anti-TIM-1 (HAVCR1) (clone REA384) Miltenyi Biotec Cat# 130-106-023
Mouse monoclonal anti-CEACAM1+CEACAM5 (clone 4/3/17) Abcam Cat# ab 91213
PE mouse monoclonal anti-ANPEP (clone WM15) BioLegend Cat# 301703
APC mouse monoclonal anti-CD34 (clone 4H11) Thermo Fisher Scientific Cat# 17-0349-41
APC mouse monoclonal anti-B7-H4 (VTCN1) (clone MIH43) BioLegend Cat# 358108
PE mouse monoclonal anti-CD249(ENPEP) (clone 2D3/APA) BD Cat# 564533
APC rat monoclonal anti-CD140a(PDGFRA) (clone APA5) Thermo Fisher Scientific Cat# 17-1401-81
PE rat monoclonal anti-Feeder cells (clone mEF-SK4) Miltenyi Biotec Cat# 130-120-166
Alexa Flour 555 Phalloidin Thermo Fisher Scientific Cat# A34055
Alexa Flour Plus 647 Phalloidin Thermo Fisher Scientific Cat# A30107
Alexa Flour 488 Donkey anti-Mouse Thermo Fisher Scientific Cat# A-21202
Alexa Flour 488 Donkey anti-Rabbit Thermo Fisher Scientific Cat# A-21206
Alexa Flour 488 Donkey anti-Goat Thermo Fisher Scientific Cat# A-11055
Alexa Flour 488 Donkey anti-Rat Thermo Fisher Scientific Cat# A-21208
Alexa Flour 555 Donkey anti-Mouse Thermo Fisher Scientific Cat# A-31570
Alexa Flour 555 Donkey anti-Rabbit Thermo Fisher Scientific Cat# A32794
Alexa Flour 555 Donkey anti-Goat Thermo Fisher Scientific Cat# A21432
Alexa Flour 555 Goat anti-Mouse Thermo Fisher Scientific Cat# A21424
Alexa Flour 555 Goat anti-Rabbit Thermo Fisher Scientific Cat# A21429
Alexa Flour 647 Donkey anti-Mouse Thermo Fisher Scientific Cat# A32787
Alexa Flour 647 Donkey anti-Rabbit Thermo Fisher Scientific Cat# A32794
Alexa Flour 647 Donkey anti-Goat Thermo Fisher Scientific Cat# A32816
Streptavidin-APC Biolegend Cat# 405207
Goat polyclonal anti-rabbit IgG, HRP-linked antibody Cell Signaling Technology Cat# 7074
Horse polyclonal anti-mouse IgG, HRP-linked antibody Cell Signaling Technology Cat# 7076

Validation

Validation statement of antibodies used in this study are available on the manufacturers' websites.

GATA6 (5851): https://www.cellsignal.jp/products/primary-antibodies/gata-6-d61e4-xp-rabbit-mab/5851
GATA6 (MAB1700): https://www.rndsystems.com/products/human-gata-6-antibody-222228_mab1700

GATA6 (AF1700): https://www.rndsystems.com/products/human-gata-6-antibody_af1700
GATA4 (sc-1237): https://www.scbt.com/ja/p/gata-4-antibody-c-20
GATA4 (14-9980-82): https://www.thermofisher.com/antibody/product/Gata-4-Antibody-clone-eBioEvan-Monoclonal/14-9980-82
SOX17 (AF1924): https://www.rndsystems.com/products/human-sox17-antibody_af1924
FOXA2 (8186): https://www.cellsignal.jp/products/primary-antibodies/foxa2-hnf3b-d56d6-xp-rabbit-mab/8186
NANOG (14-5769-82): https://www.thermofisher.com/antibody/product/Nanog-Antibody-clone-hNanog-2-Monoclonal/14-5768-82
NANOG (14-5768-80): https://www.thermofisher.com/antibody/product/Nanog-Antibody-clone-hNanog-2-Monoclonal/14-5768-80
Oct4 (sc-5279): https://www.scbt.com/ja/p/oct-3-4-antibody-c-10
Oct4 (sc-8628): https://www.scbt.com/ja/p/oct-3-4-antibody-n-19
Oct4 (2840): https://www.cellsignal.jp/products/primary-antibodies/oct-4a-c30a3-rabbit-mab/2840
GFP (04404-84): https://www.nacalai.co.jp/ss/ec2/ec-srchdetl.cfm?HP=1&l=JP&lc=1&syohin=0440484&syubetsu=3&catalog=&SiireC=&MakerC=&yoro=&mv=1
DsRed (632496): https://catalog.takara-bio.co.jp/com/manual_info.php?unitid=U100004743
PARD6B (sc-67393): https://www.scbt.com/ja/p/pard6b-antibody-m-64
PARD6B (sc-166405): https://www.scbt.com/ja/p/pard6b-antibody-b-10
PODXL (MAB1658): https://www.rndsystems.com/products/human-podocalyxin-antibody-222328_mab1658
PKCζ (sc-17781): https://www.scbt.com/ja/p/pkc-zeta-antibody-h-1
Brachyury (AF2085): https://www.rndsystems.com/products/human-mouse-brachyury-antibody_af2085
Brachyury (81694): https://www.cellsignal.jp/products/primary-antibodies/brachyury-d2z3j-rabbit-mab/81694
OTX2 (sc-514195): https://www.scbt.com/ja/p/otx2-antibody-d-8
Lefty (AF746): https://www.rndsystems.com/products/human-mouse-lefty-antibody_af746
DKK1 (48367): https://www.cellsignal.jp/products/primary-antibodies/dkk1-d5v6l-rabbit-mab/48367
Laminin (ab11575): https://www.abcam.co.jp/laminin-antibody-ab11575.html
Laminin beta-1 (PA5-27271): https://www.thermofisher.com/antibody/product/Laminin-beta-1-Antibody-Polyclonal/PA5-27271
KLF17 (HPA024629): https://www.sigmaaldrich.com/catalog/product/sigma/hpa024629?lang=ja&region=JP
GATA3 (AF2605): https://www.rndsystems.com/products/human-gata-3-antibody_af2605
TFAP2A (sc-12726): https://www.scbt.com/ja/p/ap-2alpha-antibody-3b5
ISL1&ISL2 (39.4D5): https://dshb.biology.uiowa.edu/39-4D5
ISL1 (AF1837): https://www.rndsystems.com/products/human-islet-1-antibody_af1837
CD34 (ab8158): https://www.abcam.co.jp/products/primary-antibodies/cd34-antibody-mec-147-ab8158.html
ERG (97249): https://www.cellsignal.jp/products/primary-antibodies/erg-a7l1g-rabbit-mab/97249
GATA2(NBP1-82581): https://www.novusbio.com/products/gata-2-antibody_nbp1-82581
a-tubulin (ab7291): https://www.abcam.co.jp/alpha-tubulin-antibody-dm1a-loading-control-ab7291.html
pSMAD1/5/9 (9511): https://www.cellsignal.jp/products/primary-antibodies/phospho-smad1-ser463-465-smad5-ser463-465-smad9-ser465-467-antibody/9511
pSMAD2 (3108): https://www.cellsignal.jp//products/primary-antibodies/phospho-smad2-ser465-467-138d4-rabbit-mab/3108
pSTAT3 (9131): https://www.cellsignal.jp//products/primary-antibodies/phospho-stat3-tyr705-antibody/9131
STAT3 (610189): https://www.bdbiosciences.com/ja-jp/products/reagents/western-blotting-and-molecular-reagents/purified-mouse-anti-stat3.610189
pMAPK (4376): https://www.cellsignal.jp//products/primary-antibodies/phospho-p44-42-mapk-erk1-2-thr202-tyr204-20g11-rabbit-mab/4376
PDGFRA (BAF322): https://www.rndsystems.com/products/human-pdgf-ralpha-biotinylated-antibody_baf322
HAVCR1 (130-106-023): https://www.miltenyibiotec.com/JP-en/products/tim-1-antibody-anti-human-reafinity-rea384.html#conjugate=biotin:size=100-tests-in-1-ml
CEACAM1+CEACAM5 (ab91213): https://www.abcam.co.jp/ceacam1-ceacam5-antibody-4317-ab91213.html
ANPEP (301703): https://www.biolegend.com/ja-jp/products/pe-anti-human-cd13-antibody-875?GroupID=BLG10247
CD34 (17-0349-41): https://www.thermofisher.com/antibody/product/CD34-Antibody-clone-4H11-Monoclonal/17-0349-42
PDGFRA (17-1401-81): https://www.thermofisher.com/antibody/product/CD140a-PDGFRA-Antibody-clone-APA5-Monoclonal/17-1401-81
VTCN1 (358108): https://www.biolegend.com/ja-jp/explore-new-products/apc-anti-human-b7-h4-antibody-8919?GroupID=BLG11552
ENPEP (564533): https://www.bdbiosciences.com/ja-jp/products/reagents/flow-cytometry-reagents/research-reagents/single-color-antibodies-ruo/pe-mouse-anti-human-cd249.564533
Feeder cells (130-120-166): https://www.miltenyibiotec.com/ES-en/products/feeder-cells-antibody-anti-mouse-mef-sk4.html#gref
Phalloidin AF555(A34055): https://www.thermofisher.com/order/catalog/product/A34055#/A34055
Phalloidin AF647(A30107): https://www.thermofisher.com/order/catalog/product/jp/ja/A30107
Donkey anti-Mouse IgG(H+L) AF488 (A-21202): https://www.thermofisher.com/antibody/product/Donkey-anti-Mouse-IgG-H-L-Highly-Cross-Adsorbed-Secondary-Antibody-Polyclonal/A-21202
Donkey anti-Rabbit IgG(H+L) AF488 (A-21206): https://www.thermofisher.com/antibody/product/Donkey-anti-Rabbit-IgG-H-L-Highly-Cross-Adsorbed-Secondary-Antibody-Polyclonal/A-21206
Donkey anti-Goat IgG(H+L) AF488 (A-11055): https://www.thermofisher.com/antibody/product/Donkey-anti-Goat-IgG-H-L-Cross-Adsorbed-Secondary-Antibody-Polyclonal/A-11055
Donkey anti-Rat IgG(H+L) AF488 (A-21208): https://www.thermofisher.com/antibody/product/Donkey-anti-Rat-IgG-H-L-Highly-Cross-Adsorbed-Secondary-Antibody-Polyclonal/A-21208
Donkey anti-Mouse IgG(H+L) AF555 (A-31570): https://www.thermofisher.com/antibody/product/Donkey-anti-Mouse-IgG-H-L-Highly-Cross-Adsorbed-Secondary-Antibody-Polyclonal/A-31570
Donkey anti-Rabbit IgG(H+L) AF555 (A32794): https://www.thermofisher.com/antibody/product/Donkey-anti-Rabbit-IgG-H-L-Highly-Cross-Adsorbed-Secondary-Antibody-Polyclonal/A32794
Donkey anti-Goat IgG(H+L) AF555 (A21432): https://www.thermofisher.com/antibody/product/Donkey-anti-Goat-IgG-H-L-Cross-Adsorbed-Secondary-Antibody-Polyclonal/A-21432
Goat anti-Mouse IgG(H+L) AF555 (A21424): https://www.thermofisher.com/antibody/product/Goat-anti-Mouse-IgG-H-L-Highly-Cross-Adsorbed-Secondary-Antibody-Polyclonal/A-21424
Goat anti-Rabbit IgG(H+L) AF555 (A21429): https://www.thermofisher.com/antibody/product/Goat-anti-Rabbit-IgG-H-L-Highly-Cross-Adsorbed-Secondary-Antibody-Polyclonal/A-21429
Donkey anti-Mouse IgG(H+L) AF647 (A32787): https://www.thermofisher.com/antibody/product/Donkey-anti-Mouse-IgG-H-L-Highly-Cross-Adsorbed-Secondary-Antibody-Polyclonal/A32787

Donkey anti-Rabbit IgG(H+L) AF647 (A32794): https://www.thermofisher.com/antibody/product/Donkey-anti-Rabbit-IgG-H-L-Highly-Cross-Adsorbed-Secondary-Antibody-Polyclonal/A32794
Donkey anti-Goat IgG(H+L) AF647 (A32816): https://www.thermofisher.com/antibody/product/Donkey-anti-Goat-IgG-H-L-Highly-Cross-Adsorbed-Secondary-Antibody-Polyclonal/A32816
Streptavidin-APC (405207): https://www.biolegend.com/ja-jp/products/apc-streptavidin-1470?GroupID=GROUP23
rabbit IgG, HRP-linked (7074): https://www.cellsignal.jp/products/secondary-antibodies/anti-rabbit-igg-hrp-linked-antibody/7074
mouse IgG, HRP-linked (7076): https://www.cellsignal.jp/products/secondary-antibodies/anti-mouse-igg-hrp-linked-antibody/7076

Eukaryotic cell lines

Policy information about cell lines

Cell line source(s): Human ESC lines H1 and H9 (WiCell Research Institute, Madison, WI, USA), human iPSCs generated from adult adipose-derived stem cells (AdiPSCs) (Takashima et al., 2014), human iPSCs 585B1 (Sasaki et al., 2015), 1390G3 (Yamashiro et al., 2018) and PB004, mouse ES cells (Kallkan et al. 2017) were cultured. PB004 iPS cell line is an approved iPS cell line for the interspecies chimera experiment by Japanese government.

Authentication: All cell lines have been authenticated by original sources and also authenticated in-house by observation of colony morphology, RT-qPCRs, immunostaining, RNA-seq and/or in vitro differentiation.

Mycoplasma contamination: We constantly check the contamination of mycoplasma. All cell lines are negative for mycoplasma test.

Commonly misidentified lines (See ICLAC register): No commonly misidentified cell lines were used.

Palaeontology and Archaeology

Specimen provenance: *Provide provenance information for specimens and describe permits that were obtained for the work (including the name of the issuing authority, the date of issue, and any identifying information).*

Specimen deposition: *Indicate where the specimens have been deposited to permit free access by other researchers.*

Dating methods: *If new dates are provided, describe how they were obtained (e.g. collection, storage, sample pretreatment and measurement), where they were obtained (i.e. lab name), the calibration program and the protocol for quality assurance OR state that no new dates are provided.*

☐ Tick this box to confirm that the raw and calibrated dates are available in the paper or in Supplementary Information.

Ethics oversight: *Identify the organization(s) that approved or provided guidance on the study protocol, OR state that no ethical approval or guidance was required and explain why not.*

Note that full information on the approval of the study protocol must also be provided in the manuscript.

Animals and other organisms

Policy information about studies involving animals; ARRIVE guidelines recommended for reporting animal research

Laboratory animals: Common marmoset(Callithrix jacchus) embryos: Naturally fertilized embryos were collected from the uterus by non-invasive flushing. Individual number of embryo' parents (female/male): 14725/15058, 14051/14239, 14551/14752, 14014/13584, 14551/14752, 13745/14002, 13221/12888, 14694/14320, 15143/14730, 13835/YX002. We did not confirm sex of common marmoset embryos. BDF1xB6 mouse embryos were collected at eight-cell and morula stage. Recipient female ICR mice were purchased from SLC Japan(Shizuoka, Japan). We did not confirm sex of mouse embryos.

Wild animals: The study did not involve wild animals.

Field-collected samples: The study did not involve samples collected from the field.

Ethics oversight: All animal experiments were approved by the Animal Experiment Committee at CiRA and Kyoto University (Approval number 16-75-6) and the Institutional Animal Care and Use Committee of the Central Institute for Experimental Animals (CIEA: Approval number 17029A and 18031A). Interspecies chimera formation experiment using human iPSC line PB004 were approved by the ethics committee at the University of Tokyo and by the Japanese government.

Note that full information on the approval of the study protocol must also be provided in the manuscript.

Human research participants

Policy information about studies involving human research participants

Population characteristics: *Describe the covariate-relevant population characteristics of the human research participants (e.g. age, gender, genotypic information, past and current diagnosis and treatment categories). If you filled out the behavioural & social sciences study design questions and have nothing to add here, write "See above."*

Recruitment	*Describe how participants were recruited. Outline any potential self-selection bias or other biases that may be present and how these are likely to impact results.*
Ethics oversight	*Identify the organization(s) that approved the study protocol.*

Note that full information on the approval of the study protocol must also be provided in the manuscript.

Clinical data

Policy information about clinical studies

All manuscripts should comply with the ICMJE guidelines for publication of clinical research and a completed CONSORT checklist must be included with all submissions.

Clinical trial registration	*Provide the trial registration number from ClinicalTrials.gov or an equivalent agency.*
Study protocol	*Note where the full trial protocol can be accessed OR if not available, explain why.*
Data collection	*Describe the settings and locales of data collection, noting the time periods of recruitment and data collection.*
Outcomes	*Describe how you pre-defined primary and secondary outcome measures and how you assessed these measures.*

Dual use research of concern

Policy information about dual use research of concern

Hazards

Could the accidental, deliberate or reckless misuse of agents or technologies generated in the work, or the application of information presented in the manuscript, pose a threat to:

No	Yes	
☐	☐	Public health
☐	☐	National security
☐	☐	Crops and/or livestock
☐	☐	Ecosystems
☐	☐	Any other significant area

Experiments of concern

Does the work involve any of these experiments of concern:

No	Yes	
☐	☐	Demonstrate how to render a vaccine ineffective
☐	☐	Confer resistance to therapeutically useful antibiotics or antiviral agents
☐	☐	Enhance the virulence of a pathogen or render a nonpathogen virulent
☐	☐	Increase transmissibility of a pathogen
☐	☐	Alter the host range of a pathogen
☐	☐	Enable evasion of diagnostic/detection modalities
☐	☐	Enable the weaponization of a biological agent or toxin
☐	☐	Any other potentially harmful combination of experiments and agents

ChIP-seq

Data deposition

☐ Confirm that both raw and final processed data have been deposited in a public database such as GEO.

☐ Confirm that you have deposited or provided access to graph files (e.g. BED files) for the called peaks.

Data access links<br>*May remain private before publication.*	*For "Initial submission" or "Revised version" documents, provide reviewer access links. For your "Final submission" document, provide a link to the deposited data.*
Files in database submission	*Provide a list of all files available in the database submission.*
Genome browser session<br>(e.g. UCSC)	*Provide a link to an anonymized genome browser session for "Initial submission" and "Revised version" documents only, to enable peer review. Write "no longer applicable" for "Final submission" documents.*

Methodology

Replicates	*Describe the experimental replicates, specifying number, type and replicate agreement.*
Sequencing depth	*Describe the sequencing depth for each experiment, providing the total number of reads, uniquely mapped reads, length of reads and whether they were paired- or single-end.*
Antibodies	*Describe the antibodies used for the ChIP-seq experiments; as applicable, provide supplier name, catalog number, clone name, and lot number.*
Peak calling parameters	*Specify the command line program and parameters used for read mapping and peak calling, including the ChIP, control and index files used.*
Data quality	*Describe the methods used to ensure data quality in full detail, including how many peaks are at FDR 5% and above 5-fold enrichment.*
Software	*Describe the software used to collect and analyze the ChIP-seq data. For custom code that has been deposited into a community repository, provide accession details.*

Flow Cytometry

Plots

Confirm that:

☒ The axis labels state the marker and fluorochrome used (e.g. CD4-FITC).

☒ The axis scales are clearly visible. Include numbers along axes only for bottom left plot of group (a 'group' is an analysis of identical markers).

☒ All plots are contour plots with outliers or pseudocolor plots.

☒ A numerical value for number of cells or percentage (with statistics) is provided.

Methodology

Sample preparation	Cells were dissociated into single cells by Accutase or trypsin, washed, and blocked in HBSS (Cat. 14185052, Thermo Fisher Scientific) with 1% BSA (Cat. A2153, Sigma-Aldrich) on ice for 30 min. Staining was performed on ice.
Instrument	BD LSR Fortessa (BD) or FACS Aria II (BD)
Software	Data were analysed using FlowJo.
Cell population abundance	Cell sorting was performed and the sorted populations were evaluated by qPCR.
Gating strategy	Cell population was gated by FSC/SSC and doublet cells were removed. Then dead cells were removed by DAPI and the remaining cells were analysed.<br>Gating strategies are included in the Supplementary Figure 1.

☒ Tick this box to confirm that a figure exemplifying the gating strategy is provided in the Supplementary Information.

Magnetic resonance imaging

Experimental design

Design type	*Indicate task or resting state; event-related or block design.*
Design specifications	*Specify the number of blocks, trials or experimental units per session and/or subject, and specify the length of each trial or block (if trials are blocked) and interval between trials.*
Behavioral performance measures	*State number and/or type of variables recorded (e.g. correct button press, response time) and what statistics were used to establish that the subjects were performing the task as expected (e.g. mean, range, and/or standard deviation across subjects).*

Acquisition

Imaging type(s) — *Specify: functional, structural, diffusion, perfusion.*

Field strength — *Specify in Tesla*

Sequence & imaging parameters — *Specify the pulse sequence type (gradient echo, spin echo, etc.), imaging type (EPI, spiral, etc.), field of view, matrix size, slice thickness, orientation and TE/TR/flip angle.*

Area of acquisition — *State whether a whole brain scan was used OR define the area of acquisition, describing how the region was determined.*

Diffusion MRI ☐ Used ☐ Not used

Preprocessing

Preprocessing software — *Provide detail on software version and revision number and on specific parameters (model/functions, brain extraction, segmentation, smoothing kernel size, etc.).*

Normalization — *If data were normalized/standardized, describe the approach(es): specify linear or non-linear and define image types used for transformation OR indicate that data were not normalized and explain rationale for lack of normalization.*

Normalization template — *Describe the template used for normalization/transformation, specifying subject space or group standardized space (e.g. original Talairach, MNI305, ICBM152) OR indicate that the data were not normalized.*

Noise and artifact removal — *Describe your procedure(s) for artifact and structured noise removal, specifying motion parameters, tissue signals and physiological signals (heart rate, respiration).*

Volume censoring — *Define your software and/or method and criteria for volume censoring, and state the extent of such censoring.*

Statistical modeling & inference

Model type and settings — *Specify type (mass univariate, multivariate, RSA, predictive, etc.) and describe essential details of the model at the first and second levels (e.g. fixed, random or mixed effects; drift or auto-correlation).*

Effect(s) tested — *Define precise effect in terms of the task or stimulus conditions instead of psychological concepts and indicate whether ANOVA or factorial designs were used.*

Specify type of analysis: ☐ Whole brain ☐ ROI-based ☐ Both

Statistic type for inference
(See Eklund et al. 2016) — *Specify voxel-wise or cluster-wise and report all relevant parameters for cluster-wise methods.*

Correction — *Describe the type of correction and how it is obtained for multiple comparisons (e.g. FWE, FDR, permutation or Monte Carlo).*

Models & analysis

n/a	Involved in the study
☐	☐ Functional and/or effective connectivity
☐	☐ Graph analysis
☐	☐ Multivariate modeling or predictive analysis

Functional and/or effective connectivity — *Report the measures of dependence used and the model details (e.g. Pearson correlation, partial correlation, mutual information).*

Graph analysis — *Report the dependent variable and connectivity measure, specifying weighted graph or binarized graph, subject- or group-level, and the global and/or node summaries used (e.g. clustering coefficient, efficiency, etc.).*

Multivariate modeling and predictive analysis — *Specify independent variables, features extraction and dimension reduction, model, training and evaluation metrics.*
